# Multimodal prehabilitation for major surgery in elderly patients to lower complications: protocol of a randomised, prospective, multicentre, multidisciplinary trial (PREHABIL Trial)

**DOI:** 10.1136/bmjopen-2022-070253

**Published:** 2023-01-03

**Authors:** Christian M Beilstein, Gabija Krutkyte, Thomas Vetsch, Prisca Eser, Matthias Wilhelm, Zeno Stanga, Lia Bally, Martin Verra, Markus Huber, Patrick Y Wuethrich, Dominique Engel

**Affiliations:** 1 Department of Anaesthesiology and Pain Medicine, Inselspital University Hospital, University of Bern, Bern, Switzerland; 2 Division of Diabetes, Endocrinology, Nutritional Medicine and Metabolism, Inselspital University Hospital Bern, University of Bern, Bern, Switzerland; 3 Medical Division Rehabilitation & Sports Medicine, Inselspital University Hospital, University of Bern, Bern, Switzerland; 4 Department of Diabetes, Endocrinology, Clinical Nutrition and Metabolism, Inselspital University Hospital, Bern, Switzerland; 5 Institute of Physiotherapy, Inselspital University Hospital, University of Bern, Bern, Switzerland

**Keywords:** surgery, clinical trials, geriatric medicine

## Abstract

**Introduction:**

The global volume of surgery is growing and the population ageing, and economic pressure is rising. Major surgery is associated with relevant morbidity and mortality. Postoperative reduction in physiological and functional capacity is especially marked in the elderly, multimorbid patient with low fitness level, sarcopenia and malnutrition. Interventions aiming to optimise the patient prior to surgery (prehabilitation) may reduce postoperative complications and consequently reduce health costs.

**Methods and analysis:**

This is a multicentre, multidisciplinary, prospective, 2-arm parallel-group, randomised, controlled trial with blinded outcome assessment. Primary outcome is the Comprehensive Complications Index at 30 days. Within 3 years, we aim to include 2×233 patients with a proven fitness deficit undergoing major surgery to be randomised using a computer-generated random numbers and a minimisation technique. The study intervention consists of a structured, multimodal, multidisciplinary prehabilitation programme over 2–4 weeks addressing deficits in physical fitness and nutrition, diabetes control, correction of anaemia and smoking cessation versus standard of care.

**Ethics and dissemination:**

The PREHABIL trial has been approved by the responsible ethics committee (Kantonale Ethikkomission Bern, project ID 2020-01690). All participants provide written informed consent prior to participation. Participant recruitment began in February 2022 (10 and 8 patients analysed at time of submission), with anticipated completion in 2025. Publication of the results in peer-reviewed scientific journals are expected in late 2025.

**Trial registration number:**

NCT04461301.

**STRENGTHS AND LIMITATIONS OF THIS STUDY**

- This study focuses on frail, elderly patients undergoing major surgery with a proven low functional capacity using cardiopulmonary exercise testing.
- It implements a structured, multidisciplinary, multimodal prehabilitation intervention addressing low functional capacity, malnutrition, anaemia and smoking.
- The primary endpoint is the Comprehensive Complication Index at 30 days.
- Patient-reported outcomes such as Quality of Recovery are implemented as secondary outcomes.
- The study is not blinded due to the nature of the intervention, however, primary outcome is assessed by a blinded adjudication committee.

## Introduction

### Background

The global volume of surgery has grown by about a third, from 234 million procedures in 2008 to more than 312 million in 2012, with most surgeries being performed in developed countries.^1 2^ Mortality rates are estimated to be between 1% and 4% in Europe.^3^ A minority of patients at high risk seem responsible for the majority of complications and mortality.^4^ It is, therefore, of key importance to identify patients at risk preoperatively for several reasons: it allows informed decision-making, it offers the opportunity for preoperative optimisation and it identifies patients with increased intraoperative and postoperative monitoring needs.

### Risk prediction before major surgery

Perioperative risk is largely determined by three domains: urgency of surgery, surgery-specific risk factors such as invasiveness, expected blood loss or fluid shifts, and patient-specific risks such as age, comorbidities and fitness. Whereas the first domain is usually non-modifiable, the latter two are modifiable to some extent, offering the opportunity to adjust the surgical procedure or optimise the patient’s condition prior to surgery.

Several preoperative or perioperative risk scores and prediction models have been proposed and validated, such as the Portsmouth Physiological and Operative Severity Score for the enumeration of Mortality and morbidity (P-)POSSUM, Surgical Risk Score, the American College of Surgeons National Surgical Quality Improvement Programme or the Preoperative Score to Predict Postoperative Mortality for non-cardiac surgery (NCS) or the European System for Cardiac Operative Risk (EuroScore) for cardiac surgery.^5–9^ In addition, organ-specific risk scores such as Revised Cardiac Risk Index for cardiac complications and many others have been published and widely used.^10 11^

Traditionally, patients’ reported health state, medical history and clinical assessment were used to classify the patients’ individual risk, for example, using the American Society of Anesthesiologists^12^ score. Accordingly, level of fitness was reported by the patients themselves. Based on the 2022 European Society of Cardiology/European Society of Anaesthesiology (ESC/ESA) guidelines, for example, further investigations such as (cardiac) stress testing are indicated for patients with low functional capacity (less than four metabolic equivalents of task) before high-risk elective NCS and high likelihood or proven coronary artery disease or before intermediate-risk NCS when ischaemia is of concern in patients with clinical risk factors and poor functional capacity.^13^

Patient-reported measures of functional capacity remain notoriously unreliable. Better performance was seen when using the Duke Activity Status Index (DASI) questionnaire, which correlated well with objective, cardiopulmonary exercise testing (CPET)-derived parameters such as peak oxygen consumption (VO2_peak_) and was able to predict cardiovascular events in a recent trial.^14^ While CPET remains the gold standard for global assessment of functional capacity, it is laborious for the patient and costly. Therefore, the Canadian Cardiovascular Society recommends against performing perioperative CPET to enhance cardiac risk estimation.^15^ Nevertheless, an anaerobic threshold (AT) level of <11 mL/kg/min has been confirmed as a reliable predictor for increased perioperative risk.^16 17^ This also applies to the elderly patient.^18^ In addition, the AT has the better discriminatory power to identify patients with reduced functional capacity comparted to the 6 min walking test (6MWT), which is recommended for departments lacking CPET equipment.^19^ Very recent evidence found that an increased VE/VCO2 slope might be an even more sophisticated prognostic marker to predict morbidity in high-risk patients having major cancer surgery or patients undergoing lobectomy for lung cancer.^20 21^

With increasing age, the physiological resilience to external stressors such as surgery diminishes, while the number of comorbidities rises and muscle mass decreases. In addition, elderly people are often malnourished, have a weakened immune system and impaired wound healing. All these factors contribute to a reduction in functional capacity.^22^ Despite the inconsistency of the definition of frailty, it can be assessed using a variety of tools such as the clinical frailty scale or more objective Fried frailty criteria including gait speed and handgrip strength, which are useful predictors of all-cause mortality and cardiovascular disease and death in elderly surgical and non-surgical patients.^23–25^ Similarly, standardised screening and assessments exist for the diagnosis of malnutrition and sarcopenia, which demonstrated significant overlap with frailty.^26–28^ In summary, the elderly, frail, malnourished and sarcopenic patient is especially at risk for perioperative complications, however, many options for medical, physiological and psychological optimisation exist.^29^

### Interventions to reduce perioperative risk

Organ specific, medically mandated interventions (as brought up by the preoperative assessment, such as treatment of relevant aortic or coronary stenosis) have to be completed prior to major surgery, irrespective of any study participation. However, despite clear guidelines on patient blood management, for example, relevant anaemia is not always corrected. In fact, less than half of Swiss hospitals had implemented a patient blood management programme in the year 2022.^30^

To empower the patient physically and psychologically to withstand the stress of surgery and to hasten recovery, the focus is widening from the intraoperative and postoperative to the perioperative period as a continuum. In addition, patients are believed to be more receptive for beneficial changes in behaviour prior to surgery than during the phase of recovery. Whereas rehabilitation is covering the postoperative period, the term prehabilitation has emerged for interventions occurring weeks before surgery.

As of today, prehabilitation is a complex approach with a combination of possible interventions (e.g., exercise, nutrition, psychosocial, and medical optimisation), and there is no precise definition of the type of intervention, the optimal dose nor duration required. This has led to widely differing interpretations and protocols of interventional trials with unimodal (addressing only one domain such as physical fitness, whereby also mixed-exercise programmes including aerobic, resistance and inspiratory muscles training were implemented) or multimodal (addressing additional domains such as anaemia, malnutrition, smoking behaviour and psychological support) interventions.

Many conflicting results for clinical effectiveness of prehabilitation interventions have been reported.^31–34^ The potential for cost reduction seems plausible, but good evidence from randomised controlled trials is lacking so far.^35–38^

Potential reasons for these equivocal results are several fold, with the first and most important probably being inappropriate patient selection. From eight randomised trials addressing prehabilitation before major intra-abdominal cancer surgery, only two limited the study population to ‘high-risk’ patients, based on age, American Society of Anaesthesiologists or Dukes classification, however, none of them used objectively measured physical fitness as an inclusion criteria.^39^ Selecting patients with a relatively low risk for perioperative complications will inevitably lead to a dilution of the possible positive effects and produce negative results regardless of the intervention or surgeries studied. The small or inexistent benefit to ‘low-risk’ patients does not warrant the time-demanding and resource-demanding interventions. In addition, the time required for the intervention has been found to make recruitment of large cohorts difficult.^40 41^

Second, poor results may have been due to implementing unimodal rather than individually tailored multimodal interventions that cover all domains of patients’ health state prior to surgery, such as physical fitness, malnutrition, diabetes control, anaemia, smoking and psychological well-being.

Insufficient exercise capacity is believed to be the most important predictor for postoperative complications,^42^ and therefore, mandates careful assessment with appropriate patient selection. While physical fitness interventions (even high-intensity/interval training) have been proven to be safe and feasible and have led to a measurably increased fitness levels (e.g., 6MWT), they failed to produce outcome benefits, possibly due to inappropriate patient selection, unimodality (e.g., lack of considerations of other factors such as malnutrition) and adherence.^43–46^

Further, up to one-third of surgical patients are malnourished, and malnutrition has been proven to be associated with increased morbidity, mortality and cost.^47 48^ For malnourished patients, oral nutritional supplementation, in particular adequate protein supplementation, was shown to be effective in improving outcomes.^49–51^ Malnutrition is often associated with sarcopenia and frailty, making a combined intervention of exercise training and nutritional support promising.^22 45 52^ However, so far, nutritional assessment and intervention have been poorly standardised, making conclusions regarding its efficacy and interstudy comparisons difficult.^53^

A further frequent metabolic comorbidity is diabetes affecting approximately 30%–40% of people undergoing elective surgery. Surgical outcomes are worse in people with diabetes with an up to three-fold higher risk of postoperative complications including poor healing, wound complications and renal dysfunction. Although inadequate glucose control was associated with poor surgical outcomes,^54^ no systematic strategies to improve diabetes management prior to surgery are currently recommended and only few prehabilitation programmes included diabetes optimisation as part of a multimodal concept.

Anaemia-related perioperative blood transfusions and smoking have both been found to be related to worse outcomes and increased costs.^55–57^

The negative influence of preoperative anxiety or depression on postoperative outcomes such as quality of life has gained more attention.^58 59^ Even without an proper psychosocial intervention, physical exercise can reduce psychological distress in addition to improving cardiovascular function and ultimately improve wound healing.^60^

Another factor contributing to negative results of existing multimodal prehabilitation programmes might be attributable to the simple fact that patients were randomised into the intervention group, but were not taking actively part in the programme (lack of patient adherence).^61^ Some studies fail to demonstrate this effect by not measuring adherence rates at all^12^

So the necessity of implementing a multimodal intervention seems obvious given the important contribution of anaemia, smoking, nutritional status, physical fitness and psychological well-being. Nevertheless, even for multimodal prehabilitation, the level of evidence remains low and conflicting, with inappropriate patient selection appearing to be the main reason.^32 33^

In conclusion, no trials to date have limited the study population to ‘high-risk’ patients most prone to benefit from prehabilitation. Further, only few studies have implemented a multimodal approach targeting correction of anaemia and malnutrition, smoking cessation and improving physical fitness. There is a need for trials with stringent selection of the patients who will benefit most from preoperative optimisation and implementation of personalised comorbidity mitigation and risk factor management offered in the form of individually tailored multimodal prehabilitation.^62^

### Outcome measures after major surgery

The most widely accepted postoperative hard outcome measure is mortality at 30 days.^6^ Nevertheless, extending the follow-up to at least 90 days or ideally 1 year has been proposed.^63^ To grade postoperative complications, the Clavien-Dindo classification has been established and recommended by the ESA-European Society of Intensive Care Medicine joint taskforce on perioperative outcome measures.^63–65^ There is a huge variety of possible postoperative complications, and powering studies for individual complications is nearly impossible. To overcome this difficulty, the Comprehensive Complication Index (CCI) has been developed.^66^ It is a continuous scale to measure surgical morbidity (ranging from 0=no complications to 100=death) and can be used to reduce sample size in randomised controlled trials as it provides a more sensitive endpoint.^67–69^

### Calculation of cost savings

The CCI can be used to calculate costs, as postoperative morbidity correlates highly with healthcare costs. Staiger *et al* found a 14% increase to the baseline cost for each 10-point increase in CCI.^70^ Therefore, a CCI reduction can serve as a surrogate marker for cost savings.

### Study hypothesis

We hypothesise that the implementation of a multimodal, multidisciplinary prehabilitation programme over 2–4 weeks, consisting of physical exercise, nutrition, anaemia correction and smoking cessation, will result in lower CCI within 30 days after major surgery in elderly patients.

### Feasibility: preliminary data

After ethics approval and logistic preparations, we were able to include 18 patients from March 2022 to October 2022 (table 1).

**Table 1:** Preliminary results

	Control group (n=8)	Intervention group (n=10)
Demographics/comorbidities		
Age (years)	78.25 (SD 5.4)	78.20 (SD 5.5)
Female sex	2 (25%)	1 (10%)
ASA score=III (n)	2 (25%)	2 (20%)
ASA score ≥IV (n)	6 (75%)	8 (80%)
Fried Frailty Scale (n)	1.9 (SD 1.1)	2.1 (SD 1.2)
Smoking (y/n)	1 (12.5%)	0 (0%)
Anaemia (y/n)	2 (25%)	4 (40%)
Nutritional deficit (NRS-2002≥3) (y/n)	2 (25%)	1 (10%)
Ischaemic heart disease or chronic heart failure (y/n)	4 (50%)	6 (60%)
Diabetes (y/n)	3 (37.5%)	3 (30%)
Obesity (BMI ≥30 kg/m^2^) (y/n)	1 (12.5%)	1 (10%)
Surgical procedure		
Cardiac	2	3
Orthopaedic	0	1
Thoracic	0	1
Urologic	3	4
Vascular	2	1
Visceral	1	0
Use of ECC (y/n)	2 (25%)	3 (30%)
P-POSSUM (baseline)	43.3 (SD 6.5)	45.2 (SD 8.2)
Days to surgery (days)	29.8 (SD 19.7)	28.5 (SD 9.3)
Laboratory parameters (baseline)		
Haemoglobin (g/L)	136.8 (SD 16.3)	126.9 (SD 13.8)
NT-proBNP (pg/mL)	3677.4 (SD 5022.3)	886.8 (SD 881.5)
Albumin (g/L)	33.3 (SD 3.9)	36.0 (SD 2.3)
Prealbumin (g/L)	0.22 (SD 0.04)	0.25 (SD 0.06)
HbA1c (%)	6.0 (SD 0.7)	6.4 (SD 1.4)
Creatinine (mmol/L)	147.9 (SD 51.5)	131.0 (SD 53.4)
Fitness		
MIP (cmH20)	51.3 (SD 28.0)	46.9 (SD 16.1)
AT (mL/min/kg)	9.8 (SD 1.0)	9.1 (SD 1.3)
VO2peak (mL/min/kg)	12.7 (SD 1.4)	11.8 (SD 2.8)
Questionnaires		
STAI (n)	10.6 (SD 2.4)	13 (SD3.9)
DASI (n)	4.1 (SD 0.8)	4.6 (SD 1.4)

ASA, American Society of Anesthesiologists; AT, anaerobic threshold; BMI, body mass index; DASI, Duke Activity Status Index; ECC, extracorporeal circulation; HbA1c, glycated haemoglobin; MIP, maximum inspiratory pressure; NRS-2002, Nutritional Risk Screening 2002; NT-proBNP, N-terminal-pro hormone brain-derived natriuretic peptide; STAI, state-trait anxiety inventory.

## Methods

### Study design

This is a national multicentre, multidisciplinary, prospective, two-arm parallel-group, randomised, controlled superiority trial with blinded outcome assessment. We aim to include 466 patients within 3 years. The present protocol complies with the Standard Protocol Items: Recommendations for Interventional Trials (SPIRIT) 2013 guidelines, defining standard protocol items for clinical trials.

### Participants and enrolment

**Figure 1:**
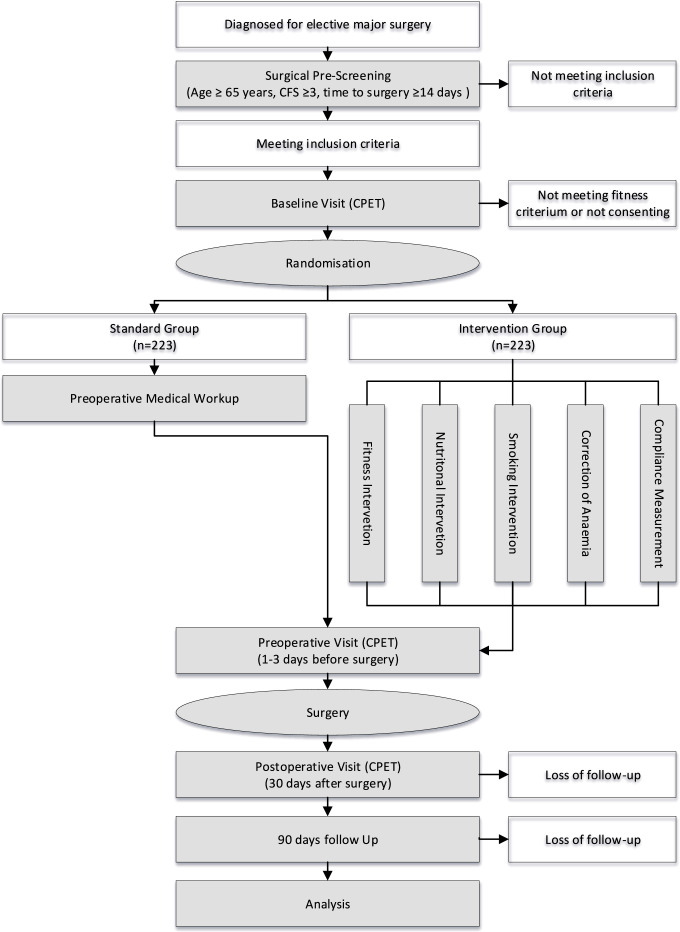
Study flow chart. CFS, Clinical Frailty Scale; CPET, cardiopulmonary exercise testing.

Surgeons scheduling the operation will prescreen the patients for frailty based on the clinical frailty scale.^71^ This tool is easy to implement in clinical practice, especially when compared with other, more sophisticated scales.^72^ If the patient achieves at least four points on the clinical frailty scale (living with very mild frailty) and there is sufficient time for prehabilitation (at least 2 weeks), informed consent will be sought, followed by a screening CPET (baseline visit, see figure 1).

### Inclusion criteria

Participants are eligible if aged 65 years or over, have planned major visceral, urologic, cardiac, vascular, orthopaedic or thoracic surgery, are classified as ASA III or higher by the attending anaesthetist, and have a proven deficit in functional capacity measured during the screening CPET defined as follows:

- VO2 at AT <11 mL/kg/min or peak VO2 <14 mL/kg/min on cycle ergometer.
- VO2 at AT <13 mL/kg/min or peak VO2 <16.5 mL/kg/min on treadmill.
- VO2 at AT <9 mL/kg/min or peak VO2 <11.3 mL/kg/min with arm crank.
- VE/VCO2 slope ≥33 (independent of modality).

Patients with VO2 above thresholds during the screening CPET will nevertheless be consented, in order to collect their CCI at 30 days. CCI at 30 days will be compared descriptively between patients qualifying and not qualifying for this study for qualitative clinical assessment outside the purpose of this study.

### Exclusion criteria

Patients are excluded if scheduled for emergency surgery, if unable to exercise for physiological or psychological reasons, if having cognitive disabilities making adherence to interventions impossible or if requiring dialysis due to logistic reasons.

### Preoperative assessments

All patients are assessed twice during the preoperative phase, once at inclusion (baseline) and once the day before surgery (preoperative). Measurements conducted during these visits include CPET, grip strength and maximal inspiratory pressure for the assessment of physical fitness. Bioelectrical impedance analysis^13^ measurements and validated nutritional screening and assessment tools (Nutrition Risk Screening 2002 (NRS-2002) and the Subjective Global Assessment [SGA]) will be performed to quantify participants’ body composition and nutritional status. Blood samples are taken to screen for anaemia (haemoglobin, reticulocyte index, renal function, iron status using transferrin saturation), nutritional and diabetic status (albumin, prealbumin, glucose, glycated haemoglobin (HbA1c)), micronutrient deficits (erythrocyte folate, holotranscobalamin, 25-hydroxy-vitamin D) and cardiac function (N-terminal-pro hormone brain-derived natriuretic peptide (NT-proBNP), high-sensitivity (hs) TroponinT). For smokers, exhaled carbon monoxide (CO) is measured (Smokerlyzer Bedfont Scientific, Kent, UK) and smoking habits evaluated by questionnaire including the Fagerström test. All patients are asked to fill in the short form of the State-Trait Anxiety Inventory,^70^ the DASI and (on the day prior to operation) the Quality of Recovery (QoR-)15 questionnaire. An activity tracker (AX3, Axivity, Newcastle, UK) is provided at the screening visit and continuously worn until the preoperative visit, when it is collected from the patient.

### Group allocation (minimisation)

After completion of the screening visit (CPET), patients who fulfil the inclusion criteria are randomised using computer-generated random numbers, into either intervention or control group. For group allocation, a probabilistic minimisation procedure is used to achieve balanced groups in regard to parameters known to be relevant for outcome after major surgery. We have included the following parameters in the minimisation algorithm (SecuTrial, V.6.1.1.10): type of surgery (visceral, urologic, cardiovascular, orthopaedic, thoracic), smoking, presence of anaemia (haemoglobin below 120 g/L in females, <130 g/L in males), nutritional deficit (NRS ≥3), age group (65–74, 75–84, 85+), ischaemic heart disease or heart failure, known diabetes, sex, obesity (body mass index (BMI) >30 kg/m^2^) and use of extracorporeal circulation.

### Patient and public involvement

Patients or the public were not involved in the design, or conduct, or reporting, or dissemination plans of our research.

### Study interventions

#### Control group

For the control group, there is a medical work up by the study team of the results obtained. In case of abnormalities found mandating medical interventions prior to surgery based on clinical judgement, these will be initiated either by the research team or the general practitioner (GP) at the patients' discretion. Regardless of existing deficits, the patient’s GP will be informed about the patient’s study participation and will receive the results of the screening assessment. All study patients wear the accelerometer between screening and preoperative visit.

#### Intervention group

For patients of the intervention group, we will implement an individually tailored, multimodal prehabilitation programme consisting of physical and respiratory training, correction of nutritional deficits and anaemia and smoking cessation counselling based on the identified deficits.

##### Physical activity

All patients perform a tailored exercise intervention based on their individual physical fitness level. The intervention includes 1–2 sessions (60 min each) with a licensed physical therapist trained in exercise prescription and 5 years of experience in the field of exercise prescription. The physical therapist will assess the patient’s physical abilities and discuss the possibilities of incorporating at least 30 min of exercise at least five times per week at home. Based on the planned surgical intervention (e.g., hip, back), the preferences and barriers of each individual patient, the physical therapist composes a tailored personal training programme. This physical training programme is composed of endurance training (e.g., walking at moderate intensity, climbing stairs), resistance training (e.g., upper limb strengthening using a resistance band, Theraband, Akron, Ohio, USA) and inspiratory muscle training (Threshold IMT, Philips Healthcare, Horgen, Switzerland). The patient receives the personal training programme either via smartphone application or as a hard copy. The non-supervised home-based exercise training sessions will be supported by illustrative materials (smartphone application with pictures and videos or brochure with pictures). Each patient receives weekly phone calls by the physical therapist to record adherence, as a motivational strategy, to discuss any boundaries to adhering to the programme and to progress the exercises if possible. An exercise intervention might look like the following programme: Respiratory: Inspiratory muscle training (IMT) at 40% of measured maximum inspiratory pressure (MIP), 60 repetitions daily (30 morning, 30 evening); Endurance: outdoor walking at moderate intensity, 30 min 5 days/week; strength: squats with bedside support, 3×10 repetitions, as deep as pain free, 5 days/week.

##### Nutritional support and management of anaemia

**Figure 2:**
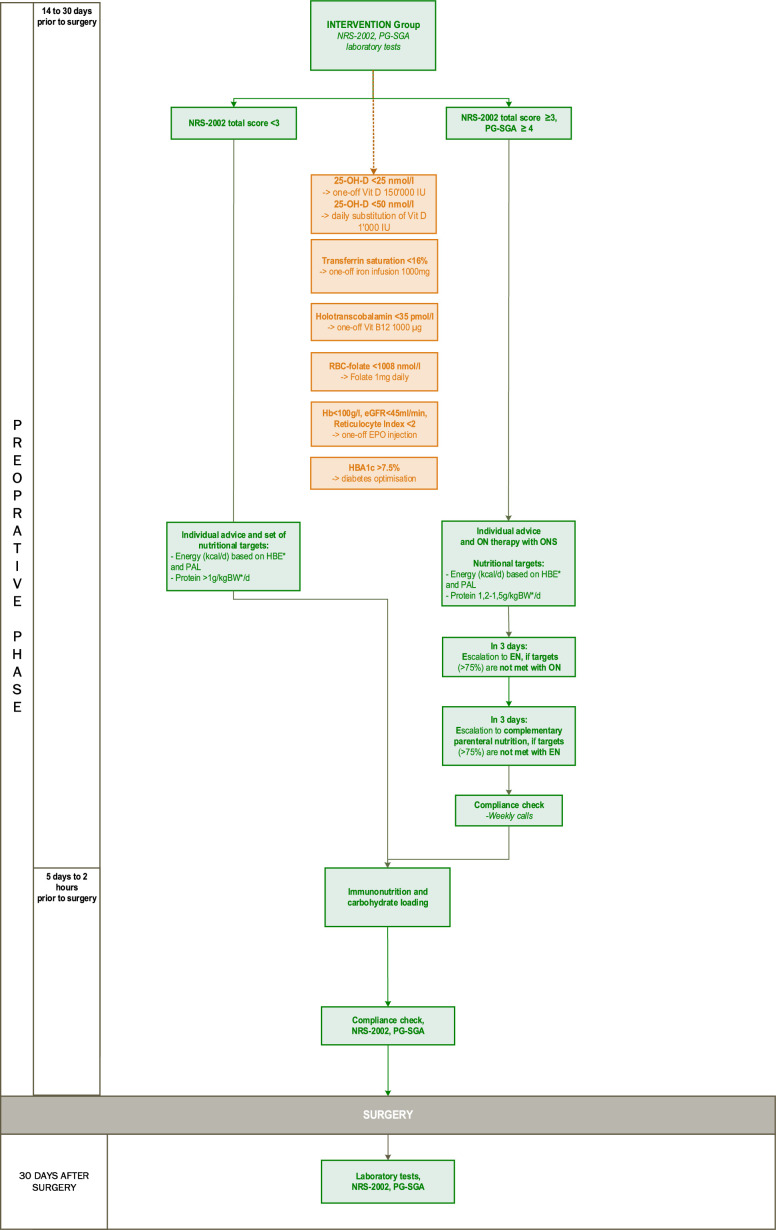
Nutrition and diabetes intervention workflow. NRS-2002, Nutrition Risk Screening 2002; PG-SGA, The Scored Patient Generated Subjective Global Assessment; 25-OH-D, 25-hydroxy-vitamin D; RBC-Folate, Erythrocyte Folate; Hb, Haemoglobin; eGFR, estimated Glomerular Filtration Rate; EPO, erythropoietin; HbA1c, glycated haemoglobin; HBE, Harris-Benedict Equation; PAL, Physical Activity Level; BW, bodyweight; ON, Oral Nutrition; ONS, Oral Nutritional Supplements; EN, Enteral Nutrition. * Usage of adjusted bodyweight, if BMI>25.

We aim to optimise the nutritional status to minimise the surgery-related catabolic stress. Nutritional risk will be assessed at baseline, preoperatively and postoperatively during study visits using the NRS-2002 total score.^73^ This validated screening tool attributes scores of different levels of nutritional status to patients based on weight loss, BMI and food intake, taking into account disease severity (degree of stress metabolism) and age. NRS-2002 total score is obtained by adding impaired nutritional status score (0–3 points), severity of disease score (0–3 points) and age (1 point if age ≥70 years). Patients are classified as being at high nutritional risk or malnourished (score ≥3) or at low risk (score <3), according to the total score obtained (see figure 2).

After screening with NRS-2002, nutritional status will be further quantified according to the patient generated SGA (PG-SGA) score, which defines the need for nutritional intervention: no intervention needed (score 0–1), needing education by a professional (score 2–3), requiring intervention by a certified dietitian (score 4–8) and having a critical need for symptom management and/or nutrient intervention (score ≥9).^74^

Patients with low risk for malnutrition (NRS-2002 <3) will be provided with dietary advice on how to achieve set individual nutritional targets (energy intake according to the Harris-Benedict equation and protein intake of >1 g/kg BW/day). Adjusted bodyweight will be used in case of BMI >25 to prevent overestimation of nutritional requirements.

Patients at high risk for being malnourished (NRS-2002 ≥3, PG-SGA ≥4) will receive dietary counselling and individualised plans to meet their energy and protein requirements. Individual energy needs will be calculated using the Harris-Benedict equation. A protein intake of 1.2–1.5 g/kg BW will be recommended to all malnourished participants (using adjusted body weight when BMI >25, as described above). To facilitate the achievement of these goals, oral nutritional supplements will be prescribed. The aim is to achieve at least 75% of the set energy and protein goal. The adherence in the malnourished group will be checked by performing 24 hours dietary recall 3 days after baseline visit. In case of reaching <75% of set dietary goals, the intervention will be escalated to enteral nutrition. If the goals are not fulfilled during the following 3 days as evaluated with 24 hours dietary recall, the intervention will be escalated to parenteral nutrition.

If scheduled for major abdominal surgery and if indication exists according to Swiss Society for Clinical Nutrition, patients will additionally receive immunonutrition (e.g., high protein oral supplements enriched with immunonutrients such as arginine, omega-3-fatty acids and nucleotides) for five consecutive days before surgery,^75 76^ as per ERAS guidelines. All participants will receive two doses of carbohydrate loading, one dose on the evening before and one up to 2 hours prior to surgery.^76^

In patients with an established or newly diagnosed diabetes and HbA1c >7.5%, glucose levels will be optimised using pre-scribed glucose monitoring and treatment intensification where appropriate. Delivered strategies represent best practice of diabetes care based on the European Association for the Study of Diabetes (EASD)/American Diabetes Association (ADA) position statement.^77^

Independent of the nutritional status, individual deficits in vitamin D, iron, vitamin B_12_ and folate will be addressed. Vitamin D is associated with muscle mass and muscle strength. Additionally, hypovitaminosis D was found to be correlated with increased risk of postoperative complications.^78 79^ It will be supplemented daily according to the blood sample values: 1000 U/day for patients with vitamin D deficiency (25-hydroxy-vitamin D <50 nmol/L) and a one-off substitution of 150 000 U for patients with severe vitamin D deficiency (25-hydroxy-vitamin D <25 nmol/L).^80^

In case of severe renal anaemia (haemoglobin <100 g/L, estimated glomerular filtration rate <45 mL/min/1.73 m^2^ and a reticulocyte index below 2), a one-off erythropoietin application (Mircera, Vifor Pharma, Switzerland) will be provided.

##### Smoking cessation

The smoking cessation programme includes intensive counselling by a graduate psychology student during three phone-call sessions as well as dispensary of nicotine replacement therapy (NRT) perioperatively if desired by the patient. NRT consists of transdermal application for moderate to heavy smokers and individual supplementation with chewing gum or lozenges (Nicotinell, GlaxoSmithKline, Brentford, UK) according to the current guidelines. NRT will either be handed out during the nutritional intervention session or sent via postal mail. The goal is to achieve a reduction in smoking quantity (cigarettes per day) of 80% before surgery.

##### Compliance with the study interventions

Adherence rates to the physical fitness intervention are primarily collected by patient-recorded standardised physical activity log books, by weekly phone calls and complemented by accelerometer data (AX3, Axivity, Newcastle, UK). Successful completion of the exercise programme is defined as adherence to 80% of prescribed exercise sessions.

For patients with an NRS-2002 ≥3 or PG-SGA ≥4, weekly phone calls by the dietician will secure adherence to the study intervention. In addition, the patients will document the intake of prescribed supplements in a log book.

The three phone call smoking cessation counselling sessions for smokers are intended to monitor and further adherence to smoking cessation. Further, exhaled CO is measured at every visit.

Reasons for non-compliance are recorded during all phone calls and strategies to increase compliance are sought in the discussion with the patient.

### Perioperative management

Perioperative management follows the usual care of the corresponding clinic, usually following standardised, multielement ERAS guidelines. Management decisions such as extension of monitoring and admission to postoperative care (postoperative care unit vs intermediate or intensive care unit (ICU)) are purely based on clinical judgement, taking the results of the preoperative testing into account as it is accessible by the clinicians through the electronic healthcare record system.

In case of raised preoperative NTproBNP >200 pg/mL, hsTroponinT will be measured 6 hours after surgery as well as on the first and second postoperative day.^81^

### Postoperative assessment

The postoperative assessment as described above will be repeated at 30 days after surgery when the patient is scheduled for surgical follow-up. The only difference from the preoperative assessment is that micronutrient and iron status are not measured again.

### Blinding procedure

This is an unblinded study due to the nature of the intervention. Nevertheless, the treating anaesthetist, while having access to the results of the preoperative assessment results via EHR, is blinded to group allocation. Primary end point adjudication committee will also be blinded (see below).

### Data collection

All data will be collected by the research team and documented in a research database (RedCap) according to local legislation. Data will be verified by dedicated members of the research team. Original source data is available in the EHR or in patients’ files.

### End point adjudication Committee

In order to standardise the review process and to reduce bias and variability in assessing the primary outcome, the CCI at 30 days will be assessed by an independent and blinded committee of three experts, which are otherwise not involved in the trial. The experts are yet to be nominated.

### Data monitoring

The data will be monitored by an external institution (Clinical Trials Unit, University of Bern) at regular intervals according to a preset monitoring plan to ensure the safety of the trial participants and the integrity of the trial data.

### Primary endpoint

Postoperative complications at 30 days after surgery scored by the CCI, assessed by a blinded, independent expert committee.

### Secondary endpoints

Secondary outcomes are measured before prehabilitation (screening visit) and thereafter (preoperative visit) and at 30 days after surgery (postoperative visit) unless otherwise specified:

#### Cardiovascular and pulmonary

- CPET: Peak VO2, VO2 at AT, peak VE, VE/VCO2 slope, oxygen uptake efficiency slope, forced expiratory volume in 1 s (FEV1), FEV1/forced vital capacity, resting HR, HR reserve, resting systolic and diastolic BP.
- Grip strength.
- Physical Function Performance 10 Test (CS 10).
- CPAx ICUD and CPAX HosD in case of ICU admission.
- MIP.
- NTproBNP.
- Serial hs-Troponin-T.
- Change in preoperative P-POSSUM score.

#### Nutrition and bioimpedance

- NRS 2002.
- PG-SGA
- Bioelectrical impedance analysis: weight, muscle mass, fat mass, percent body fat, extracellular water/intracellular water, phase angle, fat free body mass.
- Days nil per mouth (only at postoperative visit).

#### Anaemia

- Presence of anaemia.
- Transfusion in the first 30 days after surgery.

#### Smoking

- Average number of cigarettes per day, Fagerström-Score.
- Exhaled CO.

#### Patient-reported outcomes

- QoR-15.
- State-Trait Anxiety Inventory.^70^
- DASI.

#### Other outcomes of interest

- Length of hospital stay.
- Days at home at 30 days.
- Predicted costs for visceral interventions.
- CCI at 30 days postoperative assessed by the treating physician (unblinded).
- CCI at 90 days postoperative assessed by the treating physician (unblinded).

#### Safety outcomes

- Days to surgery.
- (Serious) adverse events.

### Sample size

Based on internal data (Bern University Hospital), median CCI is expected to be 30 (IQR 21–37). Assuming an average clinically relevant improvement of 15% in the intervention group compared with the control group, the sample size calculation for a non-parametric Wilcoxon-Mann-Whitney test resulted in a total patient sample of 388 (assuming a significance level alpha of 0.05 and power of 0.80). We expect a dropout rate of 20%. We, therefore, need 466 patients: 233 in each arm. We aim to complete the inclusion within 3 years.

### Statistical analysis

Data will be analysed on an intention-to-treat basis (primary outcome). Primary and secondary outcomes for the intervention and control groups will be compared.

Primary outcome: We expect that CCI will be skewed, so we will describe CCI as the median plus first and third quartiles. To test the hypothesis (H0) that the study arms result in similar CCIs (in other words, prehabilitation does not prevent postoperative complications), we will use a generalised linear model that can account for skewed data and zero inflation. The model will be adjusted for the minimisation factors used at randomisation. If no adequate model can be fit, the non-parametric Mann-Whitney U test will be used.

Secondary outcomes: All secondary outcomes will be described as means plus SD or median plus first and third quartiles for continuous data as appropriate. Categorical parameters will be described as frequencies plus percentage per time point. To account for repeat measurements of patients, we will use generalised linear mixed models to test the hypothesis of both study arms being equal in terms of secondary outcomes over time. Models will be adjusted for minimisation factors as well as baseline values (before randomisation) of the respective outcome.

Data distribution will be assessed by visual inspection of Q-Q plots.

As a secondary analysis, we will perform a PP analysis. Since the time between randomisation and surgery might differ between the two randomisation groups, we will additionally adjust generalised linear models of the primary outcome for this factor in a sensitivity analysis. Further, we will analyse the impact of adherence to the interventions to the primary outcome.

### Subgroup analysis

We will study whether effects differ between subgroups. Subgroup analyses will be performed according to the minimisation parameters (ie, surgery type, anaemia, nutritional deficit, age, frailty score, ischaemic heart disease/heart failure).

## Ethics and dissemination

The trial will be conducted according the current version of the Declaration of Helsinki, International Council for Harmonisation of Technical Requirements for Pharmaceuticals for Human Use (ICH) Guideline for Good Clinical Practice (GCP), and applicable national regulations, that is, the Human Research Act in Switzerland and has been accepted by the responsible Ethics Committee (REC) of the Canton of Berne, Switzerland (Kantonale Ethikkomission Bern 2020-01690). After initial ethics approval, subsequent protocol amendments will be submitted to the REC. Written informed consent will be obtained from all participants before entering the study.

Meetings and project presentations will be performed at the involved surgical clinics to advertise our project and raise awareness in order to motivate recruitment at regular intervals.

Results of the trial are expected to be published in major medical journals and to be presented at international congresses.

Authorship for the project will be based on the Uniform Requirements for Manuscripts of the International Committee of Medical Journal Editors (http://www.icmje.org/%23author). We followed the SPIRIT checklist when writing our report.^82^

Public access to the dataset on patient-level is not planned but possible on reasonable request.

## Supplementary Material

Reviewer comments

Author's manuscript

## References

[1] Weiser TG, Haynes AB, Molina G, et al. Size and distribution of the global volume of surgery in 2012. Bull World Health Organ 2016;94:201–9. 10.2471/BLT.15.159293 26966331 PMC4773932

[2] Weiser TG, Regenbogen SE, Thompson KD, et al. An estimation of the global volume of surgery: a modelling strategy based on available data. Lancet 2008;372:139–44. 10.1016/S0140-6736(08)60878-8 18582931

[3] Pearse RM, Moreno RP, Bauer P, et al. Mortality after surgery in Europe: a 7 day cohort study. Lancet 2012;380:1059–65. 10.1016/S0140-6736(12)61148-9 22998715 PMC3493988

[4] Pearse RM, Harrison DA, James P, et al. Identification and characterisation of the high-risk surgical population in the United Kingdom. Crit Care 2006;10:R81. 10.1186/cc4928 16749940 PMC1550954

[5] Nashef SA, Roques F, Michel P, et al. European system for cardiac operative risk evaluation (EuroSCORE). Eur J Cardiothorac Surg 1999;16:9–13. 10.1016/S1010-7940(99)00134-7 10456395

[6] Brooks MJ, Sutton R, Sarin S. Comparison of surgical risk score, POSSUM and P-POSSUM in higher-risk surgical patients. Br J Surg 2005;92:1288–92. 10.1002/bjs.5058 15981213

[7] Le Manach Y, Collins G, Rodseth R, et al. Preoperative score to predict postoperative mortality (POSPOM): derivation and validation. Anesthesiology 2016;124:570–9. 10.1097/ALN.0000000000000972 26655494

[8] Whiteley MS, Prytherch DR, Higgins B, et al. An evaluation of the POSSUM surgical scoring system. Br J Surg 1996;83:812–5. 10.1002/bjs.1800830628 8696749

[9] Bilimoria KY, Liu Y, Paruch JL, et al. Development and evaluation of the universal ACS NSQIP surgical risk calculator: a decision aid and informed consent tool for patients and surgeons. J Am Coll Surg 2013;217:833–42. 10.1016/j.jamcollsurg.2013.07.385 24055383 PMC3805776

[10] Lee TH, Marcantonio ER, Mangione CM, et al. Derivation and prospective validation of a simple index for prediction of cardiac risk of major noncardiac surgery. Circulation 1999;100:1043–9. 10.1161/01.CIR.100.10.1043 10477528

[11] Bierle DM, Raslau D, Regan DW, et al. Preoperative evaluation before noncardiac surgery. Mayo Clin Proc 2020;95:807–22. 10.1016/j.mayocp.2019.04.029 31753535

[12] Ausania F, Senra P, Meléndez R, et al. Prehabilitation in patients undergoing pancreaticoduodenectomy: a randomized controlled trial. Rev Esp Enferm Dig 2019;111:603–8. 10.17235/reed.2019.6182/2019 31232076

[13] Halvorsen S, Mehilli J, Cassese S, et al. 2022 ESC guidelines on cardiovascular assessment and management of patients undergoing non-cardiac surgery. Eur Heart J 2022;43:3826–924. 10.1093/eurheartj/ehac270 36017553

[14] Wijeysundera DN, Pearse RM, Shulman MA, et al. Assessment of functional capacity before major non-cardiac surgery: an international, prospective cohort study. Lancet 2018;391:2631–40. 10.1016/S0140-6736(18)31131-0 30070222

[15] Duceppe E, Parlow J, MacDonald P, et al. Canadian cardiovascular Society guidelines on perioperative cardiac risk assessment and management for patients who undergo noncardiac surgery. Can J Cardiol 2017;33:17–32. 10.1016/j.cjca.2016.09.008 27865641

[16] Snowden CP, Prentis JM, Anderson HL, et al. Submaximal cardiopulmonary exercise testing predicts complications and hospital length of stay in patients undergoing major elective surgery. Ann Surg 2010;251:535–41. 10.1097/SLA.0b013e3181cf811d 20134313

[17] Wilson RJT, Davies S, Yates D, et al. Impaired functional capacity is associated with all-cause mortality after major elective intra-abdominal surgery. Br J Anaesth 2010;105:297–303. 10.1093/bja/aeq128 20573634

[18] Older P, Hall A, Hader R. Cardiopulmonary exercise testing as a screening test for perioperative management of major surgery in the elderly. Chest 1999;116:355–62. 10.1378/chest.116.2.355 10453862

[19] Sinclair RCF, Batterham AM, Davies S, et al. Validity of the 6 min walk test in prediction of the anaerobic threshold before major non-cardiac surgery. Br J Anaesth 2012;108:30–5. 10.1093/bja/aer322 21980122

[20] Kristenson K, Hylander J, Boros M, et al. Ventilatory efficiency in combination with peak oxygen uptake improves risk stratification in patients undergoing lobectomy. JTCVS Open 2022;11:317–26. 10.1016/j.xjon.2022.06.018 36172418 PMC9510865

[21] Nawoor-Quinn Z, Oliver A, Raobaikady R, et al. The Marsden morbidity index: the derivation and validation of a simple risk index scoring system using cardiopulmonary exercise testing variables to predict morbidity in high-risk patients having major cancer surgery. Perioper Med 2022;11:48. 10.1186/s13741-022-00279-8 PMC9494857 36138428

[22] Csete ME. Basic science of Frailty-Biological mechanisms of age-related sarcopenia. Anesth Analg 2021;132:293–304. 10.1213/ANE.0000000000005096 32769382

[23] Bohannon RW. Hand-grip dynamometry predicts future outcomes in aging adults. J Geriatr Phys Ther 2008;31:3–10. 10.1519/00139143-200831010-00002 18489802

[24] Celis-Morales CA, Welsh P, Lyall DM, et al. Associations of grip strength with cardiovascular, respiratory, and cancer outcomes and all cause mortality: prospective cohort study of half a million UK Biobank participants. BMJ 2018;361:k1651. 10.1136/bmj.k1651 29739772 PMC5939721

[25] Chang J, Nathalie J, Nguyenhuy M, et al. Slow gait speed is associated with worse postoperative outcomes in cardiac surgery: a systematic review and meta-analysis. J Card Surg 2022;37:197–204. 10.1111/jocs.16089 34665474

[26] Burden ST, Bibby N, Donald K, et al. Nutritional screening in a cancer prehabilitation programme: a cohort study. J Human Nutrition Diet 2022;40. 10.1111/jhn.13057 35775402

[27] Cruz-Jentoft AJ, Bahat G, Bauer J, et al. Sarcopenia: revised European consensus on definition and diagnosis. Age Ageing 2019;48:16–31. 10.1093/ageing/afy169 30312372 PMC6322506

[28] Kondrup J, Allison SP, Elia M, et al. ESPEN guidelines for nutrition screening 2002. Clin Nutr 2003;22:415–21. 10.1016/S0261-5614(03)00098-0 12880610

[29] Alvarez-Nebreda ML, Bentov N, Urman RD, et al. Recommendations for preoperative management of frailty from the Society for perioperative assessment and quality improvement (SPAQI). J Clin Anesth 2018;47:33–42. 10.1016/j.jclinane.2018.02.011 29550619

[30] Previsdomini M, Colombo J, Cerutti B, et al. Dissemination of patient blood management practices in Swiss intensive care units: a cross-sectional survey. Swiss Med Wkly 2022;152:w30184. 10.4414/smw.2022.w30184 35752954

[31] Gloor S, Misirlic M, Frei-Lanter C, et al. Prehabilitation in patients undergoing colorectal surgery fails to confer reduction in overall morbidity: results of a single-center, blinded, randomized controlled trial. Langenbecks Arch Surg 2022;407:897–907. 10.1007/s00423-022-02449-0 35084526

[32] Carli F, Bousquet-Dion G, Awasthi R, et al. Effect of multimodal prehabilitation vs postoperative rehabilitation on 30-day postoperative complications for frail patients undergoing resection of colorectal cancer: a randomized clinical trial. JAMA Surg 2020;155:233–42. 10.1001/jamasurg.2019.5474 31968063 PMC6990653

[33] Perry R, Herbert G, Atkinson C, et al. Pre-Admission interventions (prehabilitation) to improve outcome after major elective surgery: a systematic review and meta-analysis. BMJ Open 2021;11:e050806. 10.1136/bmjopen-2021-050806 PMC8487197 34593498

[34] Yau DKW, Underwood MJ, Joynt GM, et al. Effect of preparative rehabilitation on recovery after cardiac surgery: a systematic review. Ann Phys Rehabil Med 2021;64:101391. 10.1016/j.rehab.2020.03.014 32446762

[35] Koh FH, Loh CH, Tan WJ, et al. Structured presurgery prehabilitation for aged patients undergoing elective surgery significantly improves surgical outcomes and reduces cost: a nonrandomized sequential comparative prospective cohort study. Nutr Clin Pract 2022;37:645–53. 10.1002/ncp.10787 34861063 PMC9299996

[36] Nielsen PR, Andreasen J, Asmussen M, et al. Costs and quality of life for prehabilitation and early rehabilitation after surgery of the lumbar spine. BMC Health Serv Res 2008;8:209. 10.1186/1472-6963-8-209 18842157 PMC2586633

[37] Ploussard G, Almeras C, Beauval J-B, et al. A combination of enhanced recovery after surgery and prehabilitation pathways improves perioperative outcomes and costs for robotic radical prostatectomy. Cancer 2020;126:4148–55. 10.1002/cncr.33061 32639601

[38] Barberan-Garcia A, Ubre M, Pascual-Argente N, et al. Post-Discharge impact and cost-consequence analysis of prehabilitation in high-risk patients undergoing major abdominal surgery: secondary results from a randomised controlled trial. Br J Anaesth 2019;123:450–6. 10.1016/j.bja.2019.05.032 31248644

[39] Thomas G, Tahir MR, Bongers BC, et al. Prehabilitation before major intra-abdominal cancer surgery: a systematic review of randomised controlled trials. Eur J Anaesthesiol 2019;36:933–45. 10.1097/EJA.0000000000001030 31188152 PMC6855314

[40] Martin D, Besson C, Pache B, et al. Feasibility of a prehabilitation program before major abdominal surgery: a pilot prospective study. J Int Med Res 2021;49:030006052110601. 10.1177/03000605211060196 PMC8649915 34851778

[41] Ricketts WM, Bollard K, Streets E, et al. Feasibility of setting up a pre-operative optimisation 'pre-hab' service for lung cancer surgery in the UK. Perioper Med 2020;9:14. 10.1186/s13741-020-00145-5 PMC7218588 32426114

[42] Hughes MJ, Hackney RJ, Lamb PJ, et al. Prehabilitation before major abdominal surgery: a systematic review and meta-analysis. World J Surg 2019;43:1661–8. 10.1007/s00268-019-04950-y 30788536

[43] O'Doherty AF, West M, Jack S, et al. Preoperative aerobic exercise training in elective intra-cavity surgery: a systematic review. Br J Anaesth 2013;110:679–89. 10.1093/bja/aes514 23393151

[44] Loughney LA, West MA, Kemp GJ, et al. Exercise interventions for people undergoing multimodal cancer treatment that includes surgery. Cochrane Database Syst Rev 2018;12:Cd012280. 10.1002/14651858.CD012280.pub2 30536366 PMC6517034

[45] Minnella EM, Awasthi R, Loiselle S-E, et al. Effect of exercise and nutrition prehabilitation on functional capacity in esophagogastric cancer surgery: a randomized clinical trial. JAMA Surg 2018;153:1081–9. 10.1001/jamasurg.2018.1645 30193337 PMC6583009

[46] Vermillion SA, James A, Dorrell RD, et al. Preoperative exercise therapy for gastrointestinal cancer patients: a systematic review. Syst Rev 2018;7:103. 10.1186/s13643-018-0771-0 30041694 PMC6058356

[47] Agarwal E, Ferguson M, Banks M, et al. Malnutrition and poor food intake are associated with prolonged hospital stay, frequent readmissions, and greater in-hospital mortality: results from the nutrition care day survey 2010. Clin Nutr 2013;32:737–45. 10.1016/j.clnu.2012.11.021 23260602

[48] Curtis LJ, Bernier P, Jeejeebhoy K, et al. Costs of hospital malnutrition. Clin Nutr 2017;36:1391–6. 10.1016/j.clnu.2016.09.009 27765524

[49] Burden S, Todd C, Hill J, et al. Pre-Operative nutrition support in patients undergoing gastrointestinal surgery. Cochrane Database Syst Rev 2012;11:CD008879. 10.1002/14651858.CD008879.pub2 23152265

[50] Elia M, Normand C, Norman K, et al. A systematic review of the cost and cost effectiveness of using standard oral nutritional supplements in the hospital setting. Clin Nutr 2016;35:370–80. 10.1016/j.clnu.2015.05.010 26123475

[51] Cawood AL, Elia M, Stratton RJ. Systematic review and meta-analysis of the effects of high protein oral nutritional supplements. Ageing Res Rev 2012;11:278–96. 10.1016/j.arr.2011.12.008 22212388

[52] Ligthart-Melis GC, Luiking YC, Kakourou A, et al. Frailty, Sarcopenia, and Malnutrition Frequently (Co-)occur in Hospitalized Older Adults: A Systematic Review and Meta-analysis. J Am Med Dir Assoc 2020;21:1216–28. 10.1016/j.jamda.2020.03.006 32327302

[53] Gillis C, Davies SJ, Carli F, et al. Current landscape of nutrition within prehabilitation oncology research: a scoping review. Front Nutr 2021;8:644723. 10.3389/fnut.2021.644723 33898499 PMC8062858

[54] Underwood P, Askari R, Hurwitz S, et al. Preoperative A1c and clinical outcomes in patients with diabetes undergoing major noncardiac surgical procedures. Diabetes Care 2014;37:611–6. 10.2337/dc13-1929 24170760

[55] Wong J, An D, Urman RD, et al. Society for perioperative assessment and quality improvement (SPAQI) consensus statement on perioperative smoking cessation. Anesth Analg 2020;131:955–68. 10.1213/ANE.0000000000004508 31764157

[56] Madjdpour C, Heindl V, Spahn DR. Risks, benefits, alternatives and indications of allogenic blood transfusions. Minerva Anestesiol 2006;72:283–98. 16675937

[57] Mehra T, Seifert B, Bravo-Reiter S, et al. Implementation of a patient blood management monitoring and feedback program significantly reduces transfusions and costs. Transfusion 2015;55:2807–15. 10.1111/trf.13260 26264557

[58] Levett DZH, Grimmett C. Psychological factors, prehabilitation and surgical outcomes: evidence and future directions. Anaesthesia 2019;74 Suppl 1:36–42. 10.1111/anae.14507 30604423

[59] Grimmett C, Bradbury K, Dalton SO, et al. The role of behavioral science in personalized multimodal prehabilitation in cancer. Front Psychol 2021;12:634223. 10.3389/fpsyg.2021.634223 33664701 PMC7921482

[60] Emery CF, Kiecolt-Glaser JK, Glaser R, et al. Exercise accelerates wound healing among healthy older adults: a preliminary investigation. J Gerontol A Biol Sci Med Sci 2005;60:1432–6. 10.1093/gerona/60.11.1432 16339330

[61] Lemanu DP, Singh PP, MacCormick AD, et al. Effect of preoperative exercise on cardiorespiratory function and recovery after surgery: a systematic review. World J Surg 2013;37:711–20. 10.1007/s00268-012-1886-4 23292047

[62] West MA, Jack S, Grocott MPW. Prehabilitation before surgery: is it for all patients? Best Pract Res Clin Anaesthesiol 2021;35:507–16. 10.1016/j.bpa.2021.01.001 34801213

[63] Jammer I, Wickboldt N, Sander M, et al. Standards for definitions and use of outcome measures for clinical effectiveness research in perioperative medicine: European perioperative clinical outcome (EPCO) definitions: a statement from the ESA-ESICM joint Taskforce on perioperative outcome measures. Eur J Anaesthesiol 2015;32:88–105. 10.1097/EJA.0000000000000118 25058504

[64] Dindo D, Demartines N, Clavien P-A. Classification of surgical complications: a new proposal with evaluation in a cohort of 6336 patients and results of a survey. Ann Surg 2004;240:205–13. 10.1097/01.sla.0000133083.54934.ae 15273542 PMC1360123

[65] Clavien PA, Barkun J, de Oliveira ML, et al. The Clavien-Dindo classification of surgical complications: five-year experience. Ann Surg 2009;250:187–96. 10.1097/SLA.0b013e3181b13ca2 19638912

[66] Slankamenac K, Graf R, Barkun J, et al. The comprehensive complication index: a novel continuous scale to measure surgical morbidity. Ann Surg 2013;258:1–7. 10.1097/SLA.0b013e318296c732 23728278

[67] Slankamenac K, Nederlof N, Pessaux P, et al. The comprehensive complication index: a novel and more sensitive endpoint for assessing outcome and reducing sample size in randomized controlled trials. Ann Surg 2014;260:757–62. discussion 62-3. 10.1097/SLA.0000000000000948 25379846

[68] Furrer MA, Huesler J, Fellmann A, et al. The comprehensive complication index CCI: a proposed modification to optimize short-term complication reporting after cystectomy and urinary diversion. Urol Oncol 2019;37:291.e9–291.e18. 10.1016/j.urolonc.2018.12.013 30638668

[69] Löffel LM, Gross T, Schneider MP, et al. Complication reporting with the Bern comprehensive complication index CCI after open radical prostatectomy: a longitudinal long-term single-center study. Urol Oncol 2020;38:79.e1–79.e8. 10.1016/j.urolonc.2019.09.021 31899103

[70] Staiger RD, Cimino M, Javed A, et al. The comprehensive complication index (CCI®) is a novel cost assessment tool for surgical procedures. Ann Surg 2018;268:784–91. 10.1097/SLA.0000000000002902 30272585

[71] Rockwood K, Song X, MacKnight C, et al. A global clinical measure of fitness and frailty in elderly people. CMAJ 2005;173:489–95. 10.1503/cmaj.050051 16129869 PMC1188185

[72] Aucoin SD, Hao M, Sohi R, et al. Accuracy and feasibility of clinically applied frailty instruments before surgery: a systematic review and meta-analysis. Anesthesiology 2020;133:78–95. 10.1097/ALN.0000000000003257 32243326

[73] Kondrup J, Rasmussen HH, Hamberg O, et al. Nutritional risk screening (NRS 2002): a new method based on an analysis of controlled clinical trials. Clin Nutr 2003;22:321–36. 10.1016/S0261-5614(02)00214-5 12765673

[74] Ottery FD. Definition of standardized nutritional assessment and interventional pathways in oncology. Nutrition 1996;12:S15–19. 10.1016/0899-9007(95)00067-4 8850213

[75] Ballmer P, Meier R, Möltgen C, et al. Richtlinien Der GESKES über home care, künstliche Ernährung zu Hause. Abgerufen am 2013;13:2021.

[76] Weimann A, Braga M, Carli F, et al. ESPEN practical guideline: clinical nutrition in surgery. Clin Nutr 2021;40:4745–61. 10.1016/j.clnu.2021.03.031 34242915

[77] Inzucchi SE, Bergenstal RM, Buse JB, et al. Management of hyperglycaemia in type 2 diabetes, 2015: a patient-centred approach. update to a position statement of the American diabetes association and the European association for the study of diabetes. Diabetologia 2015;58:429–42. 10.1007/s00125-014-3460-0 25583541

[78] Balci B, Kilinc G, Calik B, et al. The association between preoperative 25-OH vitamin D levels and postoperative complications in patients undergoing colorectal cancer surgery. BMC Surg 2021;21:369. 10.1186/s12893-021-01369-y 34666739 PMC8527669

[79] Iglar PJ, Hogan KJ. Vitamin D status and surgical outcomes: a systematic review. Patient Saf Surg 2015;9:14. 10.1186/s13037-015-0060-y 25926889 PMC4413543

[80] American Geriatrics Society Workgroup on Vitamin D Supplementation for Older Adults. Recommendations abstracted from the American geriatrics Society consensus statement on vitamin D for prevention of falls and their consequences. J Am Geriatr Soc 2014;62:147–52. 10.1111/jgs.12631 24350602

[81] Duceppe E, Patel A, Chan MTV, et al. Preoperative N-terminal pro-B-type natriuretic peptide and cardiovascular events after noncardiac surgery: a cohort study. Ann Intern Med 2020;172:96–104. 10.7326/M19-2501 31869834

[82] Chan A-W, Tetzlaff JM, Gøtzsche PC, et al. Spirit 2013 explanation and elaboration: guidance for protocols of clinical trials. BMJ 2013;346:e7586. 10.1136/bmj.e7586 23303884 PMC3541470

