# Supplementary material for: Multimodal prehabilitation for major surgery in elderly patients to lower complications: protocol of a randomised, prospective, multicentre, multidisciplinary trial (PREHABIL Trial)
Source: BMJ Open. 2023 Jan 3;13(1):e070253. doi: 10.1136/bmjopen-2022-070253 (PMC9815025; doi:10.1136/bmjopen-2022-070253)
Supplement: Author's manuscript [file bmjopen-2022-070253.draft_revisions.pdf]

BMJ Open is committed to open peer review. As part of this commitment we make the peer review history of every article we publish publicly available.

When an article is published we post the peer reviewers' comments and the authors' responses online. We also post the versions of the paper that were used during peer review. These are the versions that the peer review comments apply to.

The versions of the paper that follow are the versions that were submitted during the peer review process. They are not the versions of record or the final published versions. They should not be cited or distributed as the published version of this manuscript.

BMJ Open is an open access journal and the full, final, typeset and author-corrected version of record of the manuscript is available on our site with no access controls, subscription charges or pay-per-view fees (http://bmjopen.bmj.com).

If you have any questions on BMJ Open's open peer review process please email info.bmjopen@bmj.com

BMJ Open

Multimodal prehabilitation for major surgery in elderly patients to lower complications: Protocol of a randomised, prospective, multi-centre, multidisciplinary trial (PREHABIL Trial).

Journal:	*BMJ Open*
Manuscript ID	bmjopen-2022-070253
Article Type:	Protocol
Date Submitted by the Author:	21-Nov-2022
Complete List of Authors:	Beilstein, Christian; Inselspital University Hospital Bern, Department of Anaesthesiology and Pain Medicine, University of Bern,<br>Krutkyte, Gabija; Inselspital University Hospital Bern, Division of Diabetes, Endocrinology, Nutritional Medicine and Metabolism, University of Bern<br>Vetsch, Thomas; Inselspital University Hospital Bern, Department of Anaesthesiology and Pain Medicine, Inselspital, Bern University Hospital, University of Bern<br>Eser, Prisca; Inselspital University Hospital Bern Department of Cardiology, Division of Cardiovascular Prevention, Rehabilitation and Sports Cardiology, University Clinic for Cardiology, Inselspital, Bern University Hospital, University of Bern<br>Wilhelm, M; Inselspital University Hospital Bern Department of Cardiology, Division of Cardiovascular Prevention, Rehabilitation and Sports Cardiology, University Clinic for Cardiology, Inselspital, Bern University Hospital, University of Bern<br>Stanga, Zeno; Inselspital University Hospital Bern, Division of Diabetes, Endocrinology, Nutritional Medicine and Metabolism, Bern University Hospital, University of Bern<br>Bally, Lia; Inselspital University Hospital Bern, Department of Diabetes, Endocrinology, Clinical Nutrition and Metabolism<br>Verra, Martin; Inselspital University Hospital Bern, Institute of Physiotherapy, Bern University Hospital, University of Bern<br>Huber, Markus; Inselspital University Hospital Bern, Department of Anaesthesiology and Pain Medicine, Inselspital, Bern University Hospital, University of Bern<br>Wuethrich, Patrick; Inselspital University Hospital Bern, Department of Anaesthesiology and Pain Medicine, Inselspital, Bern University Hospital, University of Bern<br>Engel, Dominique; Inselspital University Hospital Bern, Department of Anaesthesiology and Pain Medicine, Inselspital, Bern University Hospital, University of Bern
Keywords:	SURGERY, Clinical trials < THERAPEUTICS, GERIATRIC MEDICINE

Multimodal prehabilitation for major surgery in elderly patients to lower complications: Protocol of a randomised, prospective, multicentre, multidisciplinary trial (PREHABIL Trial).

Beilstein CM[1], Krutkyte G[1,3], Vetsch T[1], Eser P[2], Wilhelm M[2], Stanga Z[3], Bally L[3], Verra ML[4], Huber M[1] Wüthrich PY[1], Engel D[1*]

1 Department of Anaesthesiology and Pain Medicine, Inselspital, Bern University Hospital, University of Bern, Switzerland
2 Division of Cardiovascular Prevention, Rehabilitation and Sports Cardiology, University Clinic for Cardiology, Inselspital, Bern University Hospital, University of Bern, Switzerland
3 Division of Diabetes, Endocrinology, Nutritional Medicine and Metabolism, Bern University Hospital, University of Bern, Switzerland
4 Institute of Physiotherapy, Bern University Hospital, University of Bern, Switzerland
*Corresponding author: Dominique.engel@insel.ch

For peer review only

Abstract

Introduction: The global volume of surgery is growing and the population ageing, and economic pressure is rising. Major surgery is associated with relevant morbidity and mortality. Postoperative reduction in physiological and functional capacity is especially marked in the elderly, multi-morbid patient with low fitness level, sarcopenia and malnutrition. Interventions aiming to optimise the patient prior to surgery (prehabilitation) may reduce post-operative complications and consequently reduce health costs.

Methods and analysis: This is a multi-centre, multidisciplinary, prospective, 2-arm parallel-group, randomised, controlled trial with blinded outcome assessment. Primary outcome is the comprehensive complications index (CCI) at 30 days. Within 3 years, we aim to include 2x233 patients with a proven fitness deficit undergoing major surgery to be randomised using a using computer-generated random numbers and a minimisation technique. The study intervention consists of a structured, multimodal, multidisciplinary prehabilitation program over 2 – 4 weeks addressing deficits in physical fitness and nutrition, diabetes control as well as correction of anaemia and smoking cessation versus standard of care.

Ethics and dissemination: The PREHABIL trial has been approved by the responsible ethics committee (KEK BE, project ID 2020-01690). All participants provide written informant consent prior to participation. Participant recruitment began in February 2022 (10 & 8 patients analysed at time of submission), with anticipated completion in 2025. Publication of the results in peer reviewed scientific journals are expected in late 2025.

Trial registration number: ClinicalTrials.gov Identifier: NCT04461301

Funding: Swiss National Science Foundation grant number FNSNF 33IC30_201298 / 1 (June 24 2021)

Protocol version: V.1, 01.11.2020

Strengths and limitations of this study:

- This study focuses on frail, elderly patients undergoing major surgery with a proven low functional capacity using cardiopulmonary exercise testing (CPET)
- It implements a structured, multidisciplinary, multimodal prehabilitation intervention addressing low functional capacity, malnutrition, anaemia and smoking.
- The primary endpoint is the comprehensive complication index at 30 days
- Patient reported outcomes such as Quality of Recovery are implemented as secondary outcomes.
- The study is not blinded due to the nature of the intervention, however primary outcome is assessed by a blinded adjudication committee.

For peer review only - http://bmjopen.bmj.com/site/about/guidelines.xhtml

Introduction

Background

The global volume of surgery has grown by about a third, from 234 million procedures in 2008 to more than 312 million in 2012, with most surgeries being performed in developed countries.[1 2] Mortality rates are estimated to be between 1-4% in Europe.[3] A minority of patients at high-risk seem responsible for the majority of complications and mortality.[4] It is therefore of key importance to identify patients at risk preoperatively for several reasons: it allows informed decision-making, it offers the opportunity for preoperative optimisation and it identifies patients with increased intra- and postoperative monitoring needs.

Risk prediction before major surgery

Perioperative risk is largely determined by three domains: urgency of surgery, surgery-specific risk factors such as invasiveness, expected blood loss or fluid shifts, and patient-specific risks such as age, comorbidities and fitness. Whereas the first domain is usually non-modifiable, the latter two are modifiable to some extent, offering the opportunity to adjust the surgical procedure or optimise the patient's condition prior to surgery.

Several pre- or perioperative risk scores and prediction models have been proposed and validated, such as the Portsmouth Physiological and Operative Severity Score for the enumeration of Mortality and morbidity (P-)POSSUM, Surgical Risk Score, the American College of Surgeons National Surgical Quality Improvement Program (NSQIP-ACS) or the Preoperative Score to Predict Postoperative Mortality (POSPOM) for non-cardiac surgery, or the European System for Cardiac Operative Risk (EuroScore) for cardiac surgery.[5-9] In addition, organ specific risk scores such as Revised Cardiac Risk Index for cardiac complications and many others have been published and widely used.[10 11]

Traditionally, patients' reported health state, medical history and clinical assessment were used to classify the patients' individual risk, e. g. using the American Society of Anesthesiologists [12] score. Accordingly, level of fitness was reported by the patients themselves. Based on the 2022 ESC/ESA guidelines for example, further investigations such as (cardiac) stress testing are indicated for patients with low functional capacity (less than 4 metabolic equivalents of task ((MET)) before high-risk elective non-cardiac surgery (NCS) and high likelihood or proven coronary artery disease. Alternatively, before intermediate-risk NCS when ischaemia is of concern in patients with clinical risk factors and poor functional capacity[13].

Patient reported measures of functional capacity remain notoriously unreliable. Better performance seen when using the Duke Activity Status Index (DASI) questionnaire, which correlated well with objective, cardiopulmonary exercise testing (CPET)-derived parameters such as peak oxygen consumption ($VO2_{peak}$) and was able to predict cardiovascular events in a recent trial.[14] While CPET remains the gold standard for global assessment of functional capacity, it is laborious for the patient and costly. Therefore, the Canadian Cardiovascular Society recommends against performing perioperative CPET to enhance cardiac risk estimation.[15] Nevertheless, an anaerobic threshold (AT) level of <11 ml kg^{-1} min^{-1} has been confirmed as a reliable predictor for increased perioperative risk.[16 17] This also applies to the elderly patient.[18] In addition, the AT has the better discriminatory power to identify patients with reduced functional capacity comparted to the 6-minutes walking test (6MWT), which is recommended for departments lacking CPET equipment.[19] Very recent evidence found that an increased VE/VCO2 slope might be an even more sophisticated prognostic marker to predict morbidity in high risk patients having major cancer surgery or patients undergoing lobectomy for lung cancer.[20 21]

For peer review only - http://bmjopen.bmj.com/site/about/guidelines.xhtml

With increasing age, the physiological resilience to external stressors such as surgery diminishes, while the number of comorbidities rises and muscle mass decrease. In addition, elderly people are often malnourished, have a weakened immune system and impaired wound healing. All these factors contribute to a reduction in functional capacity.[22] Despite the inconsistency of the definition of frailty, it can be assessed using a variety of tools such as the clinical frailty scale or more objective Fried frailty criteria including gait speed and handgrip strength, which are useful predictors of all-cause mortality and cardiovascular disease and death in elderly, surgical and non-surgical patients.[23-25] Similarly, standardised screening and assessments exist for the diagnosis of malnutrition and sarcopenia, which demonstrated significant overlap with frailty.[26-28] In summary, the elderly, frail, malnourished and sarcopenic patient is especially at risk for perioperative complications, however, many options for medical, physiological and psychological optimisation exist.[29]

Interventions to reduce perioperative risk

Organ specific, medically mandated interventions (as brought up by the preoperative assessment before treatment of relevant aortic or coronary stenosis) have to be completed prior to major surgery, irrespective of any study participation. However, despite clear guidelines for example on patient blood management, relevant anaemia is not always corrected. In fact, less than half of Swiss hospitals had implemented a patient blood management program in the year 2022.[30]

To empower the patient physically and psychologically to withstand the stress of surgery and to hasten recovery, the focus is widening from the intra- and postoperative to the perioperative period as a continuum. In addition, patients are believed to be more receptive for beneficial changes in behaviour prior to surgery than during the phase of recovery. Whereas rehabilitation is covering the postoperative period, the term prehabilitation has emerged for interventions occurring weeks before surgery.

As of today, prehabilitation is a complex approach with a combination of possible interventions (e.g. exercise, nutrition, psychosocial, medical optimization), and there is no precise definition of the type of intervention, the optimal dose nor duration required. This has led to widely differing interpretations and protocols of interventional trials with unimodal (addressing only one domain such as physical fitness, whereby also mixed-exercise programmes including aerobic, resistance and inspiratory muscles training were implemented) or multimodal (addressing additional domains such as anaemia, malnutrition, smoking behaviour and psychological support) interventions.

Many conflicting results for clinical effectiveness of prehabilitation interventions have been reported.[31-34] The potential for cost reduction seems plausible, but good evidence from randomized controlled trials is lacking so far.[35-38]

Potential reasons for these equivocal results are several fold, with the first and most important probably being inappropriate patient selection. From 8 randomised trials addressing prehabilitation before major intra-abdominal cancer surgery, only 2 limited the study population to "high risk" patients, based on age, ASA or Dukes classification, however, none of them used objectively measured physical fitness as an inclusion criteria.[39] Selecting patients with a relatively low risk for perioperative complications will inevitably lead to a dilution of the possible positive effects and produce negative results regardless of the intervention or surgeries studied. The small or inexistent benefit to “low-risk” patients does not warrant the time- and resource-demanding interventions. In addition, the time required for the intervention has been found to make recruitment of large cohorts difficult.[40 41]

For peer review only - http://bmjopen.bmj.com/site/about/guidelines.xhtml

Secondly, poor results may have been due to implementing unimodal rather than individually tailored multi-modal interventions that cover all domains of patients' health state prior to surgery, such as physical fitness, malnutrition, diabetes control, anaemia, smoking and psychological well-being.

Insufficient exercise capacity is believed to be the most important predictor for postoperative complications[42] and therefore mandates careful assessment with appropriate patient selection. While physical fitness interventions (even high-intensity/interval training) have been proven to be safe and feasible and have led to a measurably increased fitness levels (e.g. 6MWT), they failed to produce outcome benefits, possibly due to inappropriate patient selection, unimodality (e.g. lack of considerations of other factors such as malnutrition) and adherence.[43-46]

Further, up to one third of surgical patients are malnourished, and malnutrition is proven to be associated with increased morbidity, mortality and cost.[47 48] For malnourished patients, oral nutritional supplementation, in particular adequate protein supplementation, has shown to be effective in improving outcomes.[49-51] Malnutrition is often associated with sarcopenia and frailty, making a combined intervention of exercise training and nutritional support promising.[22 45 52] However, so far, nutritional assessment and intervention have been poorly standardised, making conclusions regarding its efficacy and inter-study comparisons difficult.[53]

A further frequent metabolic comorbidity is diabetes affecting approximately 30-40% of people undergoing elective surgery. Surgical outcomes are worse in people with diabetes with an up to three-fold higher risk of postoperative complications including poor healing, wound complications, and renal dysfunction. Although inadequate glucose control was associated with poor surgical outcomes[54], no systematic strategies to improve diabetes management prior to surgery are currently recommended and only few pre-habilitation programs included diabetes optimisation as part of a multi-modal concept.

Anaemia-related perioperative blood transfusions and smoking have both been found to be related to worse outcomes and increased costs.[55-57]

The negative influence of pre-operative anxiety or depression on postoperative outcomes such as quality of life have gained more attention.[58 59] Even without an proper psychosocial intervention, physical exercise can reduce psychological distress in addition to improving cardiovascular function and ultimately improve wound healing.[60]

Another factor contributing to negative results of existing multimodal prehabilitation programs might be attributable to the simple fact that patients are randomized into the intervention group, but are not taking actively part in the program (lack of patient adherence)[61]. Some studies fail to demonstrate that effect by not measuring adherence rates at all[12]

So the necessity of implementing a multimodal intervention seems obvious given the important contribution of anaemia, smoking, nutritional status, physical fitness and psychological well-being. Nevertheless, even for multimodal prehabilitation, the level of evidence remains low and conflicting, with inappropriate patient selection appearing to be the main reason.[32 33]

In conclusion, no trials to date have limited the study population to "high-risk" patients most prone to benefit from prehabilitation. Further, only few studies have implemented a multimodal approach targeting correction of anaemia and malnutrition, smoking cessation and improving physical fitness. There is a need for trials with stringent selection of the patients who will benefit most from preoperative optimisation and

For peer review only - http://bmjopen.bmj.com/site/about/guidelines.xhtml

implementation of personalised comorbidity mitigation and risk factor management offered in the form of individually tailored multimodal prehabilitation.[62]

Outcome measures after major surgery

The most widely accepted postoperative hard outcome measure is mortality at 30 days.[6] Nevertheless, extending the follow-up to at least 90 days or ideally 1 year has been proposed.[63] To grade postoperative complications, the Clavien-Dindo classification has been established and recommended by the ESA-ESICM joint taskforce on perioperative outcome measures.[63-65] There is a huge variety of possible postoperative complications, and powering studies for individual complications is nearly impossible. To overcome this difficulty, the comprehensive complication index (CCI) has been developed.[66] It is a continuous scale to measure surgical morbidity (ranging from 0= no complications to 100 = death) and can be used to reduce sample size in randomised controlled trials as it provides a more sensitive endpoint.[67-69]

Calculation of cost savings

The CCI be used to calculate costs, as postoperative morbidity correlates highly with health care costs. Staiger et al. found a 14% increase to the baseline cost for each 10-point increase in CCI.[70] Therefore, a CCI-reduction can serve as a surrogate marker for cost-savings.

Study hypothesis

We hypothesise that the implementation of a multimodal, multidisciplinary prehabilitation program over two to four weeks, consisting of physical exercise, nutrition, anaemia correction and smoking cessation, will result in lower CCI within 30 days after major surgery in elderly patients.

Feasibility: Preliminary Data

After ethics approval and logistic preparations, we were able to include 18 patients from 03/2022 until 10/2022 (Table 1).

Table 1: Preliminary Results

	**Control Group (n=8)**	**Intervention Group (n=10)**
**Demographics / Comorbidities**		
Age (years)	78.25 (SD 5.4)	78.20 (SD 5.5)
Female Sex	2 (25%)	1 (10%)
ASA Score = III (n)	2 (25%)	2 (20%)
ASA Score ≥ IV (n)	6 (75%)	8 (80%)
Fried Frailty Scale (n)	1.9 (SD 1.1)	2.1 (SD 1.2)
Smoking (y/n)	1 (12.5%)	0 (0%)
Anaemia (y/n)	2 (25%)	4 (40%)
Nutritional Deficit (NRS-2002 ≥3) (y/n)	2 (25%)	1 (10%)

For peer review only - http://bmjopen.bmj.com/site/about/guidelines.xhtml

Ischaemic Heart Disease or Chronic Heart Failure (y/n)	4 (50%)	6 (60%)
Diabetes (y/n)	3 (37.5%)	3 (30%)
Obesity (BMI ≥30 kg/m^2) (y/n)	1 (12.5%)	1 (10%)
**Surgical Procedure**		
Cardiac<br>Orthopaedic<br>Thoracic<br>Urologic<br>Vascular<br>Visceral	2<br>0<br>0<br>3<br>2<br>1	3<br>1<br>1<br>4<br>1<br>0
Use of ECC (y/n)	2 (25%)	3 (30%)
P-POSSUM (baseline)	43.3 (SD 6.5)	45.2 (SD 8.2)
Days to surgery (days)	29.8 (SD 19.7)	28.5 (SD 9.3)
**Laboratory Parameters (baseline)**		
Haemoglobin (g/L)	136.8 (SD 16.3)	126.9 (SD 13.8)
NT-proBNP (pg/mL)	3677.4 (SD 5022.3)	886.8 (SD 881.5)
Albumin (g/L)	33.3 (SD 3.9)	36.0 (SD 2.3)
Prealbumin (g/L)	0.22 (SD 0.04)	0.25 (SD 0.06)
HbA1c (%)	6.0 (SD 0.7)	6.4 (SD 1.4)
Creatinine (mmol/L)	147.9 (SD 51.5)	131.0 (SD 53.4)
**Fitness**		
MIP (cmH20)	51.3 (SD 28.0)	46.9 (SD 16.1)
AT (ml/min/kg)	9.8 (SD 1.0)	9.1 (SD 1.3)
VO2peak (ml/min/kg)	12.7 (SD 1.4)	11.8 (SD 2.8)
**Questionnaires**		
STAI (n)	10.6 (SD 2.4)	13 (SD3.9)
DASI (n)	4.1 (SD 0.8)	4.6 (SD 1.4)

For peer review only

For peer review only - http://bmjopen.bmj.com/site/about/guidelines.xhtml

ASA = American Society of Anesthesiologists, NRS-2002 = Nutritional Risk Screening 2002, AT = Anaerobic Threshold, BMI = Body Mass Index, DASI = Duke Activity Status Index, ECC = Extracorporeal Circulation, MIP = Maximum Inspiratory Pressure, STAI = State-Trait Anxiety Inventory

Methods

Study design

This is a national multi-centre, multidisciplinary, prospective, 2-arm parallel-group, randomised, controlled superiority trial with blinded outcome assessment. We aim to include 466 patients within 3 years. The present protocol complies with the SPIRIT 2013 guidelines, defining standard protocol items for clinical trials. The trial will be conducted according the current version of the Declaration of Helsinki, ICH-GCP in analogy, and applicable national regulations, i.e., the Human Research Act in Switzerland and has been accepted by the responsible Ethics Committee (REC) of the Canton of Berne, Switzerland (KEKBE 2020-01690). After initial ethics approval, subsequent protocol amendments will be submitted to the REC. Written informed consent will be obtained from all participants before entering the study.

Participants and enrolment

Surgeons scheduling the operation will pre-screen the patients for frailty based on the clinical frailty scale.[71] This tool is easy to implement in clinical practice, especially when compared to other, more sophisticated scales.[72] If the patient achieves at least 4 points on the clinical frailty scale (living with very mild frailty) and there is sufficient time for prehabilitation (at least 2 weeks), informed consent will be sought, followed by a screening CPET (baseline visit, see Figure 1).

Inclusion criteria

Participants are eligible if aged 65 years or over, have planned major visceral, urologic, cardiac, vascular, orthopaedic or thoracic surgery, are classified as ASA III or higher by the attending anaesthetist, and have a proven deficit in functional capacity measured during the screening CPET defined as follows:

- VO_2 at AT < 11 ml/kg/min or peak VO_2< 14 ml/kg/min on cycle ergometer
- VO_2 at AT < 13 ml/kg/min or peak VO_2 < 16.5 ml/kg/min on treadmill
- VO_2 at AT < 9 ml/kg/min or peak VO_2 < 11.3 ml/kg/min with arm crank
- and/or VE/VCO_2 slope ≥33 (independent of modality)

Patients with VO_2 above thresholds during the screening CPET will nevertheless be consented, in order to collect their CCI at 30 days. CCI at 30 days will be compared descriptively between patients qualifying and not qualifying for this study for qualitative clinical assessment outside the purpose of this study.

Exclusion criteria

Patients are excluded if scheduled for emergency surgery, if unable to exercise for physiological or psychological reasons, if having cognitive disabilities making adherence to interventions impossible or if requiring dialysis due to logistic reasons.

Preoperative assessments

All patients are assessed twice during the preoperative phase, once at inclusion (baseline) and once the day before surgery (preoperative). Measurements conducted during these visits include CPET, grip strength and maximal inspiratory pressure for the assessment of physical fitness. Bio-electrical impedance analysis [13] measurements and validated nutritional screening and assessment tools (Nutrition Risk Screening 2002 (NRS-2002) and the Subjective Global Assessment (SGA)) will be performed to quantify

For peer review only - http://bmjopen.bmj.com/site/about/guidelines.xhtml

participants' body composition and nutritional status. Blood samples are taken to screen for anaemia (haemoglobin, reticulocyte index, renal function, iron status using transferrin saturation), nutritional and diabetic status (albumin, prealbumin, glucose, HbA1c), micronutrient deficits (erythrocyte folate, holotranscobalamin, 25-hydroxy-vitamin D) and cardiac function (NT-proBNP, hsTroponinT). For smokers, exhaled CO is measured (Smokerlyzer® Bedfont® Scientific Ltd, Kent, UK) and smoking habits evaluated by questionnaire including the Fagerström test. All patients are asked to fill in the short form of the State-Trait Anxiety Inventory [70], the DASI and (on the day prior to operation) the Quality of Recovery (QoR-)15 questionnaire. An activity tracker (Axivity® Accelerometer, Axivity Ltd, Newcastle, United Kingdom) is provided at the screening visit and continuously worn until the preoperative visit, when it is collected from the patient.

Group allocation (minimisation)

After completion of the screening visit (CPET), patients who fulfil the inclusion criteria are randomised using computer-generated random numbers, into either intervention or control group. For group allocation, a probabilistic minimisation procedure is used to achieve balanced groups in regards to parameters known to be relevant for outcome after major surgery. We have included the following parameters in the minimisation algorithm (SecuTrial, Version 6.1.1.10): type of surgery (visceral, urologic, cardiovascular, orthopaedic, thoracic), smoking, presence of anaemia (haemoglobin below 120 g/L in females, < 130 g/L in males), nutritional deficit (NRS ≥3), age group (65-74, 75-84, 85+), ischaemic heart disease, known diabetes, sex, obesity (body mass index >30 kg/m^2), and use of extracorporeal circulation.

Patient and Public involvement

Patients or the public were not involved in the design, or conduct, or reporting, or dissemination plans of our research.

Study interventions

Control group

For the control group, there is a medical work up by the study team of the results obtained. In case of abnormalities found mandating medical interventions prior to surgery based on clinical judgement, these will be initiated either by the research team or the general practitioner (GP) at the patients' discretion. Regardless of existing deficits, the patient's GP will be informed about the patient's study participation and will receive the results of the screening assessment. All study patients wear the accelerometer between screening and preoperative visit.

Intervention group

For patients of the intervention group, we will implement an individually tailored, multimodal prehabilitation program consisting of physical and respiratory training, correction of nutritional deficits and anaemia and smoking cessation counselling based on the identified deficits.

Physical activity

All patients perform a tailored exercise intervention based on their individual physical fitness level. The intervention includes 1 to 2 sessions (60 minutes each) with a licensed physical therapist trained in exercise prescription and 5 years of experience in the field of exercise prescription. The physical therapist will assess the patient's physical abilities and discuss the possibilities of incorporating at least 30 minutes of exercise at least 5 times per week at home. Based on the planned surgical intervention (e.g. hip, back, etc.), the preferences and barriers of each individual patient, the physical therapist composes a tailored personal

For peer review only - http://bmjopen.bmj.com/site/about/guidelines.xhtml

training programme. This physical training program is composed of endurance training (e.g. walking at moderate intensity, climbing stairs), resistance training (e.g. upper limb strengthening using a resistance band, Theraband, Akron, Ohio, USA) and inspiratory muscle training (Threshold IMT, Philips Healthcare, Horgen, Switzerland). The patient receives the personal training programme either via smartphone application or as a hard copy. The non-supervised home-based exercise training sessions will be supported by illustrative materials (smartphone application with pictures and videos or brochure with pictures). Each patient receives weekly phone calls by the physical therapist to record adherence, as a motivational strategy, to discuss any boundaries to adhering to the programme and to progress the exercises if possible. A exercise intervention might look like the following program: Respiratory: IMT, 40% of measures MIP, 60 repetitions daily (30 morning, 30 evening); Endurance: outdoor walking at moderate intensity, 30 minutes 5 days/week; Strength: Squats with bedside support, 3x10 repetitions, as deep as pain free, 5 days/week.

Nutritional support and management of anaemia

We aim to optimise the nutritional status to minimise the surgery-related catabolic stress. Nutritional risk will be assessed at baseline, preoperatively and postoperatively during study visits using the NRS-2002 total score.[73] This validated screening tool attributes scores of different level of nutritional status to patients based on weight loss, body mass index (BMI) and food intake, taking into account disease severity (degree of stress metabolism) and age. NRS-2002 total score is obtained by adding impaired nutritional status score (0-3 points), severity of disease score (0-3 points), and age (1 point if age ≥ 70 years). Patients are classified as being at high nutritional risk or malnourished (score ≥3) or at low risk (score <3), according to the total score obtained (see figure 2).

After screening with NRS-2002, nutritional status will be further quantified according to the PG-SGA score, which defines the need for nutritional intervention: no intervention needed (score 0-1), needing education by a professional (score 2-3), requiring intervention by a certified dietitian (score 4-8) and having a critical need for symptom management and/or nutrient intervention (score ≥9).[74]

Patients with low risk for malnutrition (NRS-2002 < 3) will be provided with dietary advice on how to achieve set individual nutritional targets (energy intake according to the Harris-Benedict equation and protein intake of >1 g/kg BW/day). Adjusted bodyweight will be used in case of BMI > 25 to prevent overestimation of nutritional requirements.

Patients at high risk for being malnourished (NRS-2002 ≥3, PG-SGA ≥4) will receive dietary counselling and individualised plans to meet their energy and protein requirements. Individual energy needs will be calculated using Harris-Benedict equation. A protein intake of 1.2-1.5 g/kg BW will be recommended to all malnourished participants (using adjusted body weight when BMI>25, as described above). To facilitate the achievement of these goals, oral nutritional supplements will be prescribed. The aim is to achieve at least 75% of the set energy and protein goal. The adherence in malnourished group will be checked by performing 24h dietary recall 3 days after baseline visit. In case of reaching <75% of set dietary goals, the intervention will be escalated to enteral nutrition. If the goals are not fulfilled during the following three days as evaluated with 24h dietary recall, the intervention will be escalated to parenteral nutrition.

If scheduled for major abdominal surgery and if indication exists according to Swiss Society for Clinical Nutrition, patients will additionally receive immunonutrition (e.g. high protein oral supplements enriched with immunonutrients such as arginine, omega-3-fatty acids and nucleotides) for five consecutive days before surgery.[75 76] , as per ERAS guidelines. All participants will receive 2 doses of carbohydrate loading, one dose on the evening before and one up to 2 hours prior to surgery.[76]

For peer review only - http://bmjopen.bmj.com/site/about/guidelines.xhtml

In patients with an established or newly diagnosed diabetes and HbA1c>7.5%, glucose levels will be optimised using pre-scribed glucose monitoring and treatment intensification where appropriate. Delivered strategies represent best practice of diabetes care based on the EASD-ADA position statement.[77]

Independent of the nutritional status, individual deficits in vitamin D, iron, vitamin B12 and folate will be addressed. Vitamin D is associated with muscle mass and muscle strength. Additionally, hypovitaminosis D was found to be correlating with increased risk of postoperative complications.[78 79] It will be supplemented daily according to the blood sample values: 1000 U/d for patients with vitamin D deficiency (25-hydroxy-vitamin D <50 nmol/l) and a one-off substitution of 150 000 U for patients with severe vitamin D deficiency (25-hydroxy-vitamin D < 25 nmol/l).[80]

In case of severe renal anaemia (haemoglobin < 100 g/L, eGFR < 45 ml/min/1.73m^2 and a reticulocyte index below 2), a one-off erythropoietin application (Mircera®, Vifor Pharma, Switzerland) will be provided.

Smoking cessation

The smoking cessation program includes intensive counselling by an advanced psychology student during three phone-call sessions as well as dispensary of nicotine replacement therapy (NRT) perioperatively if desired by the patient. NRT consist of transdermal application for moderate to heavy smokers and individual supplementation with chewing gum or lozenges (Nicotinell®, GlaxoSmithKline, Brentford, United Kingdom) according to the current guidelines. NRT will either be handed out during the nutritional intervention session or sent via postal mail. The goal is to achieve a reduction in smoking quantity (cigarettes per day) of 80% before surgery.

Compliance with the study interventions

Adherence rates to the physical fitness intervention are primarily collected by patient-recorded standardised physical activity log books, by via weekly phone calls and completed by accelerometer data (Axivity AX3, Axivity Ltd, Newcastle, UK). Successful completion of the exercise program is defined as adherence to 80% of prescribed exercise sessions.

For patients with a NRS-2002 ≥3 or PG-SGA ≥4, weekly phone calls by the dietician will secure adherence to the study intervention. In addition, the patients will document the intake of prescribed supplements in a log book.

The 3 phone call smoking cessation counselling sessions for smokers are intended to monitor and further adherence to smoking cessation. Further, exhaled CO is measured at every visit.

Reasons for non-compliance are recorded during all phone calls and strategies to increase compliance are sought in the discussion with the patient.

Perioperative management

Perioperative management follows the usual care of the corresponding clinic, usually following standardised, multi-element ERAS guidelines. Management decisions such as extension of monitoring and mission to postoperative care (postoperative care unit versus intermediate or intensive care unit) are purely based on clinical judgement, taking the results of the pre-operative testing into account as it is accessible by the clinicians trough the electronic health care record [57] system.

For peer review only - http://bmjopen.bmj.com/site/about/guidelines.xhtml

In case of raised preoperative NTproBNP >200 pg/ml, hsTroponinT will be measured 6 hours after surgery as well as on the first and second postoperative day.[81]

Postoperative assessment

The postoperative assessment as described above will be repeated at 30 days after surgery when the patient is scheduled for surgical follow up. The only difference from the preoperative assessment is that micronutrient and iron status are not measured again.

Blinding procedure

This is an unblinded study due to the nature of the intervention. Nevertheless, the treating anaesthetist, while having access to the results of the preoperative assessment results via EHR, is blinded to group allocation. Primary end point adjudication committee will also be blinded (see below).

Data collection

All data will be collected by the research team and documented in a research database (RedCap) according to local legislation. Data will be verified by dedicated members of the research team. Original source data is available in the EHR or in patients` files.

End point adjudication committee

In order to standardise the review process and to reduce bias and variability in assessing the primary outcome, the CCI at 30 days will be assessed by an independent and blinded committee of three experts, which are otherwise not involved in the trial. The experts are yet to be nominated.

Data monitoring

The data will be monitored by an external institution (Clinical Trials Unit, University of Bern) at regular intervals according to a pre-set monitoring plan to ensure the safety of the trial participants and the integrity of the trial data.

Primary endpoint

Postoperative complications at 30 days after surgery scored by the Comprehensive Complication Index (CCI), assessed by a blinded, independent expert committee.

Secondary endpoints

Secondary outcomes are measured before prehabilitation (screening visit) and thereafter (preoperative visit) and at 30 days after surgery (postoperative visit) unless otherwise specified:

Cardiovascular & Pulmonary

- Cardio-Pulmonary Exercise Test (CPET): Peak VO2, VO2 at AT, peak VE, VE/VCO2 slope, Oxygen Uptake Efficiency Slope (OUES), FEV1, FEV1/FVC, resting HR, HR reserve, resting systolic and diastolic BP
- Grip strength
- Maximum inspiratory pressure (MIP)
- NTproBNP
- Serial hs-Troponin-T
- Change in preoperative P-POSSUM score

Nutrition & Bio-Impedance

- Nutrition Risk Screening Score 2002

For peer review only - http://bmjopen.bmj.com/site/about/guidelines.xhtml

- Patient Generated Subjective Global Assessment- Bioelectrical impedance analysis: weight, muscle mass, fat mass, percent body fat, extracellular water/intracellular water, phase angle, Fat Free Body Mass (FFM)
- Days nil per mouth (only at postoperative visit)

Anaemia
- Presence of anaemia
- Transfusion in the first 30 days after surgery

Smoking
- Average number of cigarettes per day, Fagerström-Score
- Exhaled CO

Patient Reported Outcomes
- Quality of Recovery Score (QoR-15)
- State-Trait Anxiety Inventory [70]
- Duke Activity Status Index (DASI)

Other outcomes of interest
- Length of hospital stay
- Days at home at 30 days
- Predicted costs for visceral interventions
- CCI at 30 days assessed by the treating physician (unblinded)
- CCI at 90 days assessed by the treating physician (unblinded)

Safety outcomes
- Days to surgery
- (Serious) adverse events

Sample size

Based on internal data (Bern University Hospital), median CCI is expected to be 30 (IQR 21 to 37). Assuming an average clinically relevant improvement of 15% in the intervention group compared to the control group, the sample size calculation for a non-parametric Wilcoxon-Mann-Whitney test resulted in a total patient sample of 388 (assuming a significance level alpha of 0.05 and power of 0.80). We expect a dropout rate of 20%. We therefore need 466 patients: 233 in each arm. We aim to complete the inclusion within 3 years.

Statistical analysis

Data will be analysed on an intention-to-treat basis (primary outcome). Primary and secondary outcomes for the intervention and control groups will be compared. An interim analysis is planned when half of the intended number of subjects have completed the 4 week assessment (i.e. timing of the primary endpoint assessment).

Primary outcome: We expect that CCI will be skewed, so we will describe CCI as the median plus first and third quartiles. To test the hypothesis (H0) that the study arms result in similar CCIs (in other words, prehabilitation does not prevent postoperative complications), we will use a generalized linear model that can account for skewed data and zero inflation. The model will be adjusted for the minimization factors used at randomization. If no adequate model can be fit, the non-parametric Mann-Whitney U test will be used.

For peer review only - http://bmjopen.bmj.com/site/about/guidelines.xhtml

Secondary outcomes: All secondary outcomes will be described as means plus SD or median plus first and third quartiles for continuous data as appropriate. Categorical parameters will be described as frequencies plus percentage per time point. To account for repeat measurements of patients, we will use generalized linear mixed models to test the hypothesis of both study arms being equal in terms of secondary outcomes over time. Models will be adjusted for minimization factors as well as baseline values (before randomization) of the respective outcome.

Data distribution will be assessed by visual inspection of Q-Q plots.

As a secondary analysis, we will perform a PP analysis. Since the time between randomization and surgery might differ between the two randomization groups, we will additionally adjust generalized linear models of the primary outcome for this factor in a sensitivity analysis. Further, we will analyse the impact of adherence to the interventions to the primary outcome.

Subgroup analysis

We will study whether effects differ between subgroups. Subgroup analyses will be performed according to the minimisation parameters (i.e. surgery type, anaemia, nutritional deficit, age, frailty score, ischaemic heart disease).

Dissemination

Meetings and project presentations will be performed at the involved surgical clinics to advertise our project and raise awareness in order to motivate recruitment at regular intervals.

Results of the trial are expected to be published in major medical journals and to be presented at international congresses.

Authorship for the project will be based on the Uniform Requirements for Manuscripts of the International Committee of Medical Journal Editors (http://www.icmje.org/#author). We followed the SPIRIT checklist when writing our report.[82]

Public access to the dataset on patient-level is not planned but possible on reasonable request.

Discussion and Conclusion

There is supportive evidence of the safety and feasibility of preoperative interventions, but evidence for clinical effectiveness in regard to morbidity, mortality and patients reported outcome measures is lacking. The PREHABIL trial is aiming to reduce this gap by implementing strict selection of frail patients with low functional capacity.

This study uses a holistic approach to individually address patients' deficits by structured, multimodal, multidisciplinary interventions: physical fitness deficit (strength, endurance and inspiratory training), malnutrition (oral nutritional supplements, protein supplementation, carbohydrate loading, vitamin substitution, optimisation of diabetes), anaemia (classification and therapy) and smoking (cessation counselling and nicotine replacement therapy).

The CCI as primary outcome is highly relevant for patients and health care providers, as complications correlate closely with impaired recovery and increased costs. In addition, patient reported outcome measures are included as secondary outcomes.

For peer review only - http://bmjopen.bmj.com/site/about/guidelines.xhtml

This study has some limitations:–it is resource intensive and might not be reproducible in different economic settings. It is impossible to blind participants due to the nature of the intervention. While the primary outcome will be assessed by a blinded adjudication committee, secondary outcomes like CCI at 90 days will not be blinded.

In summary, we examine the effect of a holistic, structured, multimodal preoperative intervention in frail elderly patients undergoing major surgery on postoperative complications. By reducing complications and therefore health care cost, the intervention may be cost saving in this specific group of patients and guide future research.

Authors' contributions: all authors equally contributed to the protocol. All authors have read and approved the final manuscript.

Funding: This work is supported by the Swiss National Science Foundation grant number FNSNF 33IC30_201298 / 1 (June 24 2021), www.snf.ch

Competing interests: None of the authors declares to have a conflict of interest.

For peer review only

For peer review only - http://bmjopen.bmj.com/site/about/guidelines.xhtml

References

1. Weiser TG, Haynes AB, Molina G, et al. Size and distribution of the global volume of surgery in 2012. *Bull World Health Organ* 2016;94(3):201-09f. doi: 10.2471/blt.15.159293 [published Online First: 2016/03/12]

2. Weiser TG, Regenbogen SE, Thompson KD, et al. An estimation of the global volume of surgery: a modelling strategy based on available data. *Lancet* 2008;372(9633):139-44. doi: 10.1016/s0140-6736(08)60878-8 [published Online First: 2008/06/28]

3. Pearse RM, Moreno RP, Bauer P, et al. Mortality after surgery in Europe: a 7 day cohort study. *Lancet* 2012;380(9847):1059-65. doi: 10.1016/s0140-6736(12)61148-9 [published Online First: 2012/09/25]

4. Pearse RM, Harrison DA, James P, et al. Identification and characterisation of the high-risk surgical population in the United Kingdom. *Crit Care* 2006;10(3):R81. doi: 10.1186/cc4928 [published Online First: 2006/06/06]

5. Nashef SA, Roques F, Michel P, et al. European system for cardiac operative risk evaluation (EuroSCORE). *Eur J Cardiothorac Surg* 1999;16(1):9-13. doi: 10.1016/s1010-7940(99)00134-7 [published Online First: 1999/08/24]

6. Brooks MJ, Sutton R, Sarin S. Comparison of Surgical Risk Score, POSSUM and p-POSSUM in higher-risk surgical patients. *Br J Surg* 2005;92(10):1288-92. doi: 10.1002/bjs.5058 [published Online First: 2005/06/28]

7. Le Manach Y, Collins G, Rodseth R, et al. Preoperative Score to Predict Postoperative Mortality (POSPOM): Derivation and Validation. *Anesthesiology* 2016;124(3):570-9. doi: 10.1097/aln.0000000000000972 [published Online First: 2015/12/15]

8. Whiteley MS, Prytherch DR, Higgins B, et al. An evaluation of the POSSUM surgical scoring system. *Br J Surg* 1996;83(6):812-5. doi: 10.1002/bjs.1800830628 [published Online First: 1996/06/01]

9. Bilimoria KY, Liu Y, Paruch JL, et al. Development and Evaluation of the Universal ACS NSQIP Surgical Risk Calculator: A Decision Aid and Informed Consent Tool for Patients and Surgeons. *J Am Coll Surg* 2013;217(5):833-42e3. doi: 10.1016/j.jamcollsurg.2013.07.385

10. Lee TH, Marcantonio ER, Mangione CM, et al. Derivation and prospective validation of a simple index for prediction of cardiac risk of major noncardiac surgery. *Circulation* 1999;100(10):1043-9. doi: 10.1161/01.cir.100.10.1043 [published Online First: 1999/09/08]

11. Bierle DM, Raslau D, Regan DW, et al. Preoperative Evaluation Before Noncardiac Surgery. *Mayo Clin Proc* 2020;95(4):807-22. doi: 10.1016/j.mayocp.2019.04.029 [published Online First: 2019/11/23]

12. Ausania F, Senra P, Melendez R, et al. Prehabilitation in patients undergoing pancreaticoduodenectomy: a randomized controlled trial. *Revista Espanola de Enfermedades Digestivas*;111(8):603-08.

13. Halvorsen S, Mehilli J, Cassese S, et al. 2022 ESC Guidelines on cardiovascular assessment and management of patients undergoing non-cardiac surgery: Developed by the task force for cardiovascular assessment and management of patients undergoing non-cardiac surgery of the European Society of Cardiology (ESC) Endorsed by the European Society of Anaesthesiology and Intensive Care (ESAIC). *European Heart Journal* 2022;43(39):3826-924. doi: 10.1093/eurheartj/ehac270

14. Wijeysundera DN, Pearse RM, Shulman MA, et al. Assessment of functional capacity before major non-cardiac surgery: an international, prospective cohort study. *Lancet* 2018;391(10140):2631-40. doi: 10.1016/s0140-6736(18)31131-0 [published Online First: 2018/08/03]

15. Duceppe E, Parlow J, MacDonald P, et al. Canadian Cardiovascular Society Guidelines on Perioperative Cardiac Risk Assessment and Management for Patients Who Undergo Noncardiac Surgery. *Can J Cardiol* 2017;33(1):17-32. doi: 10.1016/j.cjca.2016.09.008 [published Online First: 2016/11/21]

For peer review only - http://bmjopen.bmj.com/site/about/guidelines.xhtml

16. Snowden CP, Prentis JM, Anderson HL, et al. Submaximal cardiopulmonary exercise testing predicts complications and hospital length of stay in patients undergoing major elective surgery. *Ann Surg* 2010;251(3):535-41. doi: 10.1097/SLA.0b013e3181cf811d [published Online First: 2010/02/06]
17. Wilson RJ, Davies S, Yates D, et al. Impaired functional capacity is associated with all-cause mortality after major elective intra-abdominal surgery. *Br J Anaesth* 2010;105(3):297-303. doi: 10.1093/bja/aeq128 [published Online First: 2010/06/25]
18. Older P, Hall A, Hader R. Cardiopulmonary exercise testing as a screening test for perioperative management of major surgery in the elderly. *Chest* 1999;116(2):355-62. doi: 10.1378/chest.116.2.355 [published Online First: 1999/08/24]
19. Sinclair RC, Batterham AM, Davies S, et al. Validity of the 6 min walk test in prediction of the anaerobic threshold before major non-cardiac surgery. *Br J Anaesth* 2012;108(1):30-5. doi: 10.1093/bja/aer322 [published Online First: 2011/10/08]
20. Kristenson K, Hylander J, Boros M, et al. Ventilatory efficiency in combination with peak oxygen uptake improves risk stratification in patients undergoing lobectomy. *JTCVS Open* 2022;11:317-26. doi: 10.1016/j.xjon.2022.06.018 [published Online First: 2022/09/30]
21. Nawoor-Quinn Z, Oliver A, Raobaikady R, et al. The Marsden Morbidity Index: the derivation and validation of a simple risk index scoring system using cardiopulmonary exercise testing variables to predict morbidity in high-risk patients having major cancer surgery. *Perioperative Medicine* 2022;11(1):48. doi: 10.1186/s13741-022-00279-8
22. Csete ME. Basic Science of Frailty-Biological Mechanisms of Age-Related Sarcopenia. *Anesth Analg* 2021;132(2):293-304. doi: 10.1213/ane.0000000000005096 [published Online First: 2020/08/10]
23. Bohannon RW. Hand-grip dynamometry predicts future outcomes in aging adults. *J Geriatr Phys Ther* 2008;31(1):3-10. doi: 10.1519/00139143-200831010-00002 [published Online First: 2008/05/21]
24. Celis-Morales CA, Welsh P, Lyall DM, et al. Associations of grip strength with cardiovascular, respiratory, and cancer outcomes and all cause mortality: prospective cohort study of half a million UK Biobank participants. *BMJ* 2018;361:k1651. doi: 10.1136/bmj.k1651 [published Online First: 2018/05/10]
25. Chang J, Nathalie J, Nguyenhuy M, et al. Slow gait speed is associated with worse postoperative outcomes in cardiac surgery: A systematic review and meta-analysis. *J Card Surg* 2022;37(1):197-204. doi: 10.1111/jocs.16089 [published Online First: 2021/10/20]
26. Burden ST, Bibby N, Donald K, et al. Nutritional screening in a cancer prehabilitation programme: A cohort study. *J Hum Nutr Diet* 2022 doi: 10.1111/jhn.13057 [published Online First: 2022/07/02]
27. Cruz-Jentoft AJ, Bahat G, Bauer J, et al. Sarcopenia: revised European consensus on definition and diagnosis. *Age Ageing* 2019;48(1):16-31. doi: 10.1093/ageing/afy169 [published Online First: 2018/10/13]
28. Kondrup J, Allison SP, Elia M, et al. ESPEN guidelines for nutrition screening 2002. *Clin Nutr* 2003;22(4):415-21. doi: 10.1016/s0261-5614(03)00098-0 [published Online First: 2003/07/26]
29. Alvarez-Nebreda ML, Bentov N, Urman RD, et al. Recommendations for Preoperative Management of Frailty from the Society for Perioperative Assessment and Quality Improvement (SPAQI). *J Clin Anesth* 2018;47:33-42. doi: 10.1016/j.jclinane.2018.02.011 [published Online First: 2018/03/20]
30. Previsdomini M, Colombo J, Cerutti B, et al. Dissemination of patient blood management practices in Swiss intensive care units: a cross-sectional survey. *Swiss Med Wkly* 2022;152:w30184. doi: 10.4414/smw.2022.w30184 [published Online First: 2022/06/27]
31. Gloor S, Misirlic M, Frei-Lanter C, et al. Prehabilitation in patients undergoing colorectal surgery fails to confer reduction in overall morbidity: results of a single-center, blinded, randomized controlled trial. *Langenbecks Arch Surg* 2022 doi: 10.1007/s00423-022-02449-0 [published Online First: 2022/01/28]
32. Carli F, Bousquet-Dion G, Awasthi R, et al. Effect of Multimodal Prehabilitation vs Postoperative Rehabilitation on 30-Day Postoperative Complications for Frail Patients Undergoing Resection of

For peer review only - http://bmjopen.bmj.com/site/about/guidelines.xhtml

Colorectal Cancer: A Randomized Clinical Trial. *JAMA Surg* 2020;155(3):233-42. doi: 10.1001/jamasurg.2019.5474 [published Online First: 2020/01/23]

33. Perry R, Herbert G, Atkinson C, et al. Pre-admission interventions (prehabilitation) to improve outcome after major elective surgery: a systematic review and meta-analysis. *BMJ Open* 2021;11(9):e050806. doi: 10.1136/bmjopen-2021-050806 [published Online First: 2021/10/02]
34. Yau DKW, Underwood MJ, Joynt GM, et al. Effect of preparative rehabilitation on recovery after cardiac surgery: A systematic review. *Ann Phys Rehabil Med* 2021;64(2):101391. doi: 10.1016/j.rehab.2020.03.014 [published Online First: 2020/05/25]
35. Koh FH, Loh CH, Tan WJ, et al. Structured presurgery prehabilitation for aged patients undergoing elective surgery significantly improves surgical outcomes and reduces cost: A nonrandomized sequential comparative prospective cohort study. *Nutr Clin Pract* 2022;37(3):645-53. doi: 10.1002/ncp.10787 [published Online First: 2021/12/04]
36. Nielsen PR, Andreasen J, Asmussen M, et al. Costs and quality of life for prehabilitation and early rehabilitation after surgery of the lumbar spine. *BMC Health Serv Res* 2008;8:209. doi: 10.1186/1472-6963-8-209 [published Online First: 2008/10/10]
37. Ploussard G, Almeras C, Beauval JB, et al. A combination of enhanced recovery after surgery and prehabilitation pathways improves perioperative outcomes and costs for robotic radical prostatectomy. *Cancer* 2020;126(18):4148-55. doi: 10.1002/cncr.33061 [published Online First: 2020/07/09]
38. Barberan-Garcia A, Ubre M, Pascual-Argente N, et al. Post-discharge impact and cost-consequence analysis of prehabilitation in high-risk patients undergoing major abdominal surgery: secondary results from a randomised controlled trial. *Br J Anaesth* 2019;123(4):450-56. doi: 10.1016/j.bja.2019.05.032 [published Online First: 2019/06/30]
39. Thomas G, Tahir MR, Bongers BC, et al. Prehabilitation before major intra-abdominal cancer surgery: A systematic review of randomised controlled trials. *Eur J Anaesthesiol* 2019;36(12):933-45. doi: 10.1097/eja.0000000000001030 [published Online First: 2019/06/13]
40. Martin D, Besson C, Pache B, et al. Feasibility of a prehabilitation program before major abdominal surgery: a pilot prospective study. *J Int Med Res* 2021;49(11):3000605211060196. doi: 10.1177/03000605211060196 [published Online First: 2021/12/02]
41. Ricketts WM, Bollard K, Streets E, et al. Feasibility of setting up a pre-operative optimisation 'pre-hab' service for lung cancer surgery in the UK. *Perioperative Medicine* 2020;9(1):14. doi: 10.1186/s13741-020-00145-5
42. Hughes MJ, Hackney RJ, Lamb PJ, et al. Prehabilitation Before Major Abdominal Surgery: A Systematic Review and Meta-analysis. *World J Surg* 2019;43(7):1661-68. doi: 10.1007/s00268-019-04950-y [published Online First: 2019/02/23]
43. O'Doherty AF, West M, Jack S, et al. Preoperative aerobic exercise training in elective intra-cavity surgery: a systematic review. *Br J Anaesth* 2013;110(5):679-89. doi: 10.1093/bja/aes514 [published Online First: 2013/02/09]
44. Loughney LA, West MA, Kemp GJ, et al. Exercise interventions for people undergoing multimodal cancer treatment that includes surgery. *Cochrane Database Syst Rev* 2018;12(12):Cd012280. doi: 10.1002/14651858.CD012280.pub2 [published Online First: 2018/12/12]
45. Minnella EM, Awasthi R, Loiselle S-E, et al. Effect of Exercise and Nutrition Prehabilitation on Functional Capacity in Esophagogastric Cancer Surgery: A Randomized Clinical Trial. *JAMA Surgery* 2018;153(12):1081-89. doi: 10.1001/jamasurg.2018.1645
46. Vermillion SA, James A, Dorrell RD, et al. Preoperative exercise therapy for gastrointestinal cancer patients: a systematic review. *Syst Rev* 2018;7(1):103. doi: 10.1186/s13643-018-0771-0 [published Online First: 2018/07/26]
47. Agarwal E, Ferguson M, Banks M, et al. Malnutrition and poor food intake are associated with prolonged hospital stay, frequent readmissions, and greater in-hospital mortality: Results from the

For peer review only - http://bmjopen.bmj.com/site/about/guidelines.xhtml

Nutrition Care Day Survey 2010. *Clin Nutr* 2013;32(5):737-45. doi: https://doi.org/10.1016/j.clnu.2012.11.021
48. Curtis LJ, Bernier P, Jeejeebhoy K, et al. Costs of hospital malnutrition. *Clin Nutr* 2017;36(5):1391-96. doi: https://doi.org/10.1016/j.clnu.2016.09.009
49. Burden S, Todd C, Hill J, et al. Pre-operative nutrition support in patients undergoing gastrointestinal surgery. *Cochrane database of systematic reviews (Online)* 2012;11:CD008879.
50. Elia M, Normand C, Norman K, et al. A systematic review of the cost and cost effectiveness of using standard oral nutritional supplements in the hospital setting. *Clin Nutr* 2016;35(2):370-80. doi: 10.1016/j.clnu.2015.05.010 [published Online First: 2015/07/01]
51. Cawood AL, Elia M, Stratton RJ. Systematic review and meta-analysis of the effects of high protein oral nutritional supplements. *Ageing Research Reviews* 2012;11(2):278-96. doi: https://doi.org/10.1016/j.arr.2011.12.008
52. Ligthart-Melis GC, Luiking YC, Kakourou A, et al. Frailty, Sarcopenia, and Malnutrition Frequently (Co-)occur in Hospitalized Older Adults: A Systematic Review and Meta-analysis. *J Am Med Dir Assoc* 2020;21(9):1216-28. doi: https://doi.org/10.1016/j.jamda.2020.03.006
53. Gillis C, Davies SJ, Carli F, et al. Current Landscape of Nutrition Within Prehabilitation Oncology Research: A Scoping Review. *Front Nutr* 2021;8:644723. doi: 10.3389/fnut.2021.644723 [published Online First: 2021/04/27]
54. Underwood P, Askari R, Hurwitz S, et al. Preoperative A1C and clinical outcomes in patients with diabetes undergoing major noncardiac surgical procedures. *Diabetes Care* 2014;37(3):611-6. doi: 10.2337/dc13-1929 [published Online First: 2013/10/31]
55. Wong J, An D, Urman RD, et al. Society for Perioperative Assessment and Quality Improvement (SPAQI) Consensus Statement on Perioperative Smoking Cessation. *Anesth Analg* 2020;131(3):955-68. doi: 10.1213/ane.0000000000004508 [published Online First: 2019/11/26]
56. Madjdpour C, Heindl V, Spahn DR. Risks, benefits, alternatives and indications of allogenic blood transfusions. *Minerva Anestesiol* 2006;72(5):283-98. [published Online First: 2006/05/06]
57. Mehra T, Seifert B, Bravo-Reiter S, et al. Implementation of a patient blood management monitoring and feedback program significantly reduces transfusions and costs. *Transfusion* 2015;55(12):2807-15. doi: 10.1111/trf.13260 [published Online First: 2015/08/13]
58. Levett DZH, Grimmett C. Psychological factors, prehabilitation and surgical outcomes: evidence and future directions. *Anaesthesia* 2019;74 Suppl 1:36-42. doi: 10.1111/anae.14507 [published Online First: 2019/01/04]
59. Grimmett C, Bradbury K, Dalton SO, et al. The Role of Behavioral Science in Personalized Multimodal Prehabilitation in Cancer. *Front Psychol* 2021;12:634223. doi: 10.3389/fpsyg.2021.634223 [published Online First: 2021/03/06]
60. Emery CF, Kiecolt-Glaser JK, Glaser R, et al. Exercise accelerates wound healing among healthy older adults: a preliminary investigation. *J Gerontol A Biol Sci Med Sci* 2005;60(11):1432-6. doi: 10.1093/gerona/60.11.1432 [published Online First: 2005/12/13]
61. Lemanu DP, Singh PP, MacCormick AD, et al. Effect of preoperative exercise on cardiorespiratory function and recovery after surgery: a systematic review. *World J Surg* 2013;37(4):711-20. doi: 10.1007/s00268-012-1886-4 [published Online First: 2013/01/08]
62. West MA, Jack S, Grocott MPW. Prehabilitation before surgery: Is it for all patients? *Best Practice & Research Clinical Anaesthesiology* 2021;35(4):507-16. doi: https://doi.org/10.1016/j.bpa.2021.01.001
63. Jammer I, Wickboldt N, Sander M, et al. Standards for definitions and use of outcome measures for clinical effectiveness research in perioperative medicine: European Perioperative Clinical Outcome (EPCO) definitions: a statement from the ESA-ESICM joint taskforce on perioperative outcome measures. *Eur J Anaesthesiol* 2015;32(2):88-105. doi: 10.1097/EJA.0000000000000118 [published Online First: 2014/07/25]

For peer review only - http://bmjopen.bmj.com/site/about/guidelines.xhtml

64. Dindo D, Demartines N, Clavien PA. Classification of surgical complications: a new proposal with evaluation in a cohort of 6336 patients and results of a survey. *Ann Surg* 2004;240(2):205-13. doi: 10.1097/01.sla.0000133083.54934.ae [published Online First: 2004/07/27]
65. Clavien PA, Barkun J, de Oliveira ML, et al. The Clavien-Dindo classification of surgical complications: five-year experience. *Ann Surg* 2009;250(2):187-96. doi: 10.1097/SLA.0b013e3181b13ca2 [published Online First: 2009/07/30]
66. Slankamenac K, Graf R, Barkun J, et al. The comprehensive complication index: a novel continuous scale to measure surgical morbidity. *Ann Surg* 2013;258(1):1-7. doi: 10.1097/SLA.0b013e318296c732 [published Online First: 2013/06/04]
67. Slankamenac K, Nederlof N, Pessaux P, et al. The comprehensive complication index: a novel and more sensitive endpoint for assessing outcome and reducing sample size in randomized controlled trials. *Ann Surg* 2014;260(5):757-62; discussion 62-3. doi: 10.1097/sla.0000000000000948 [published Online First: 2014/11/08]
68. Furrer MA, Huesler J, Fellmann A, et al. The Comprehensive Complication Index CCI: A proposed modification to optimize short-term complication reporting after cystectomy and urinary diversion. *Urologic oncology* 2019;37(4):291 e9-91 e18. doi: 10.1016/j.urolonc.2018.12.013
69. Loffel LM, Gross T, Schneider MP, et al. Complication reporting with the Bern Comprehensive Complication Index CCI after open radical prostatectomy: A longitudinal long-term single-center study. *Urologic oncology* 2020;38(3):79 e1-79 e8. doi: 10.1016/j.urolonc.2019.09.021
70. Staiger RD, Cimino M, Javed A, et al. The Comprehensive Complication Index (CCI®) is a Novel Cost Assessment Tool for Surgical Procedures. *Ann Surg* 2018;268(5):784-91. doi: 10.1097/sla.0000000000002902 [published Online First: 2018/10/03]
71. Rockwood K, Song X, MacKnight C, et al. A global clinical measure of fitness and frailty in elderly people. *CMAJ* 2005;173(5):489-95. doi: 10.1503/cmaj.050051 [published Online First: 2005/09/01]
72. Aucoin SD, Hao M, Sohi R, et al. Accuracy and Feasibility of Clinically Applied Frailty Instruments before Surgery: A Systematic Review and Meta-analysis. *Anesthesiology* 2020;133(1):78-95. doi: 10.1097/aln.0000000000003257 [published Online First: 2020/04/04]
73. Kondrup J, Rasmussen HH, Hamberg O, et al. Nutritional risk screening (NRS 2002): a new method based on an analysis of controlled clinical trials. *Clin Nutr* 2003;22(3):321-36. doi: 10.1016/s0261-5614(02)00214-5 [published Online First: 2003/05/27]
74. Ottery FD. Definition of standardized nutritional assessment and interventional pathways in oncology. *Nutrition* 1996;12(1 Suppl):S15-9. doi: 10.1016/0899-9007(96)90011-8 [published Online First: 1996/01/01]
75. Ballmer P, Meier R, Möltgen C, et al. Richtlinien der GESKES über Home Care, künstliche Ernährung zu Hause. *Abgerufen am* 2013;13:2021.
76. Weimann A, Braga M, Carli F, et al. ESPEN practical guideline: Clinical nutrition in surgery. *Clinical Nutrition* 2021;40(7):4745-61. doi: https://doi.org/10.1016/j.clnu.2021.03.031
77. Inzucchi SE, Bergenstal RM, Buse JB, et al. Management of hyperglycaemia in type 2 diabetes, 2015: a patient-centred approach. Update to a position statement of the American Diabetes Association and the European Association for the Study of Diabetes. *Diabetologia* 2015;58(3):429-42. doi: 10.1007/s00125-014-3460-0 [published Online First: 2015/01/15]
78. Balci B, Kilinc G, Calik B, et al. The association between preoperative 25-OH vitamin D levels and postoperative complications in patients undergoing colorectal cancer surgery. *BMC Surgery* 2021;21(1):369. doi: 10.1186/s12893-021-01369-y
79. Iglar PJ, Hogan KJ. Vitamin D status and surgical outcomes: a systematic review. *Patient Saf Surg* 2015;9:14. doi: 10.1186/s13037-015-0060-y [published Online First: 2015/05/01]
80. Recommendations abstracted from the American Geriatrics Society Consensus Statement on vitamin D for Prevention of Falls and Their Consequences. *J Am Geriatr Soc* 2014;62(1):147-52. doi: 10.1111/jgs.12631 [published Online First: 2013/12/20]

For peer review only - http://bmjopen.bmj.com/site/about/guidelines.xhtml

81. Duceppe E, Patel A, Chan MTV, et al. Preoperative N-Terminal Pro-B-Type Natriuretic Peptide and Cardiovascular Events After Noncardiac Surgery: A Cohort Study. *Ann Intern Med* 2020;172(2):96-104. doi: 10.7326/m19-2501 [published Online First: 2019/12/24]
82. Chan AW, Tetzlaff JM, Gøtzsche PC, et al. SPIRIT 2013 explanation and elaboration: guidance for protocols of clinical trials. *Bmj* 2013;346:e7586. doi: 10.1136/bmj.e7586 [published Online First: 2013/01/11]

For peer review only

Figure 1: Study Flow Chart. CFS = clinical frailty scale, CPET = cardiopulmonary exercise testing

Figure 2: Nutrition workflow. NRS-2002 = Nutrition Risk Screening 2002, PG-SGA = The Scored Patient Generated Subjective Global Assessment, RBC Folate = Erythrocyte Folate, Hb = Haemoglobin, eGFR = estimated Glomerular Filtration Rate, HbA1c = glycated haemoglobin, BW = bodyweight, BMR = Basal Metabolic Rate.

For peer review only

For peer review only - http://bmjopen.bmj.com/site/about/guidelines.xhtml

Figure 1: Study Flow Chart

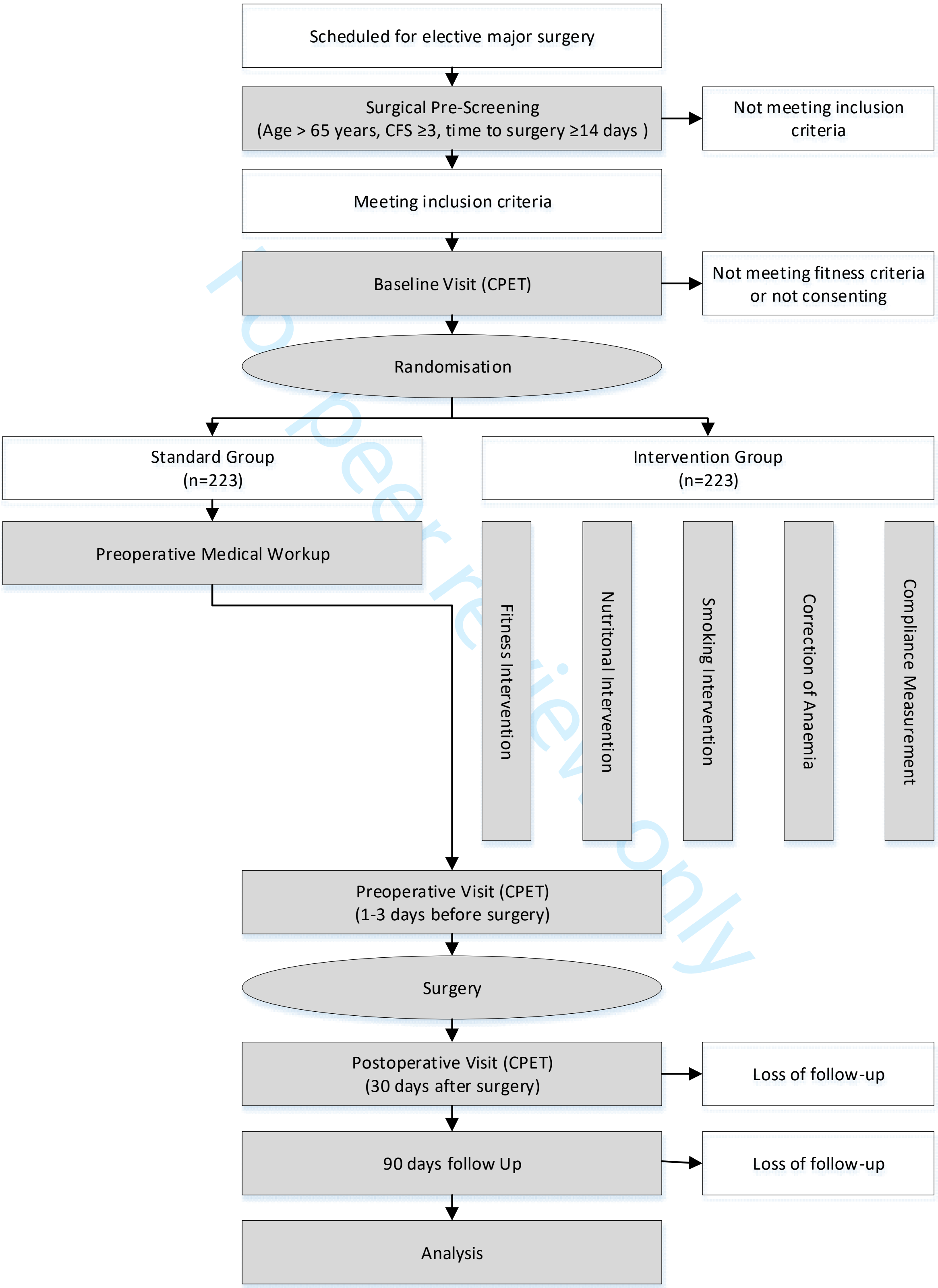

Figure 2: Nutrition workflow.

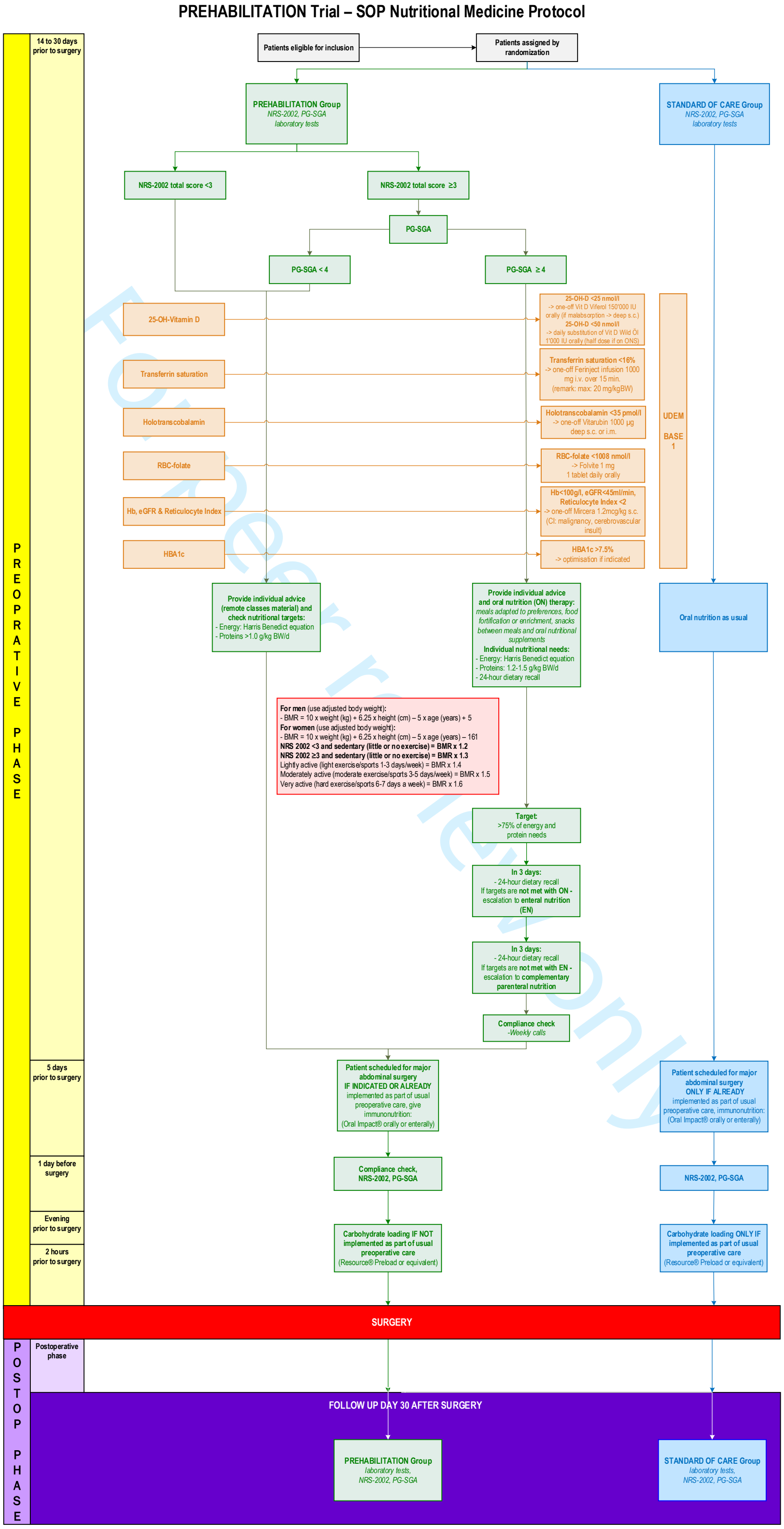

For peer review only

Reporting checklist for protocol of a clinical trial.

Based on the SPIRIT guidelines.

Instructions to authors

Complete this checklist by entering the page numbers from your manuscript where readers will find each of the items listed below.

Your article may not currently address all the items on the checklist. Please modify your text to include the missing information. If you are certain that an item does not apply, please write "n/a" and provide a short explanation.

Upload your completed checklist as an extra file when you submit to a journal.

In your methods section, say that you used the SPIRITreporting guidelines, and cite them as:

Chan A-W, Tetzlaff JM, Gøtzsche PC, Altman DG, Mann H, Berlin J, Dickersin K, Hróbjartsson A, Schulz KF, Parulekar WR, Krleža-Jerić K, Laupacis A, Moher D. SPIRIT 2013 Explanation and Elaboration: Guidance for protocols of clinical trials. BMJ. 2013;346:e7586

		Reporting Item	Page Number
**Administrative information**			
Title	#1	Descriptive title identifying the study design, population, interventions, and, if applicable, trial acronym	1
Trial registration	#2a	Trial identifier and registry name. If not yet registered, name of intended registry	2
Trial registration: data set	#2b	All items from the World Health Organization Trial Registration Data Set	2
Protocol version	#3	Date and version identifier	2
Funding	#4	Sources and types of financial, material, and other support	2
Roles and responsibilities: contributorship	#5a	Names, affiliations, and roles of protocol contributors	1, 14

For peer review only

Roles and responsibilities: sponsor contact information	#5b	Name and contact information for the trial sponsor	14
Roles and responsibilities: sponsor and funder	#5c	Role of study sponsor and funders, if any, in study design; collection, management, analysis, and interpretation of data; writing of the report; and the decision to submit the report for publication, including whether they will have ultimate authority over any of these activities	14
Roles and responsibilities: committees	#5d	Composition, roles, and responsibilities of the coordinating centre, steering committee, endpoint adjudication committee, data management team, and other individuals or groups overseeing the trial, if applicable (see Item 21a for data monitoring committee)	n/a, IICT
**Introduction**			
Background and rationale	#6a	Description of research question and justification for undertaking the trial, including summary of relevant studies (published and unpublished) examining benefits and harms for each intervention	3
Background and rationale: choice of comparators	#6b	Explanation for choice of comparators	3
Objectives	#7	Specific objectives or hypotheses	6
Trial design	#8	Description of trial design including type of trial (eg, parallel group, crossover, factorial, single group), allocation ratio, and framework (eg, superiority, equivalence, non-inferiority, exploratory)	7
**Methods: Participants, interventions, and outcomes**			
Study setting	#9	Description of study settings (eg, community clinic, academic hospital) and list of countries where data will be collected. Reference to where list of study sites can be obtained	7

For peer review only

Eligibility criteria	#10	Inclusion and exclusion criteria for participants. If applicable, eligibility criteria for study centres and individuals who will perform the interventions (eg, surgeons, psychotherapists)	8
Interventions: description	#11a	Interventions for each group with sufficient detail to allow replication, including how and when they will be administered	9
Interventions: modifications	#11b	Criteria for discontinuing or modifying allocated interventions for a given trial participant (eg, drug dose change in response to harms, participant request, or improving / worsening disease)	n/a, not intended
Interventions: adherance	#11c	Strategies to improve adherence to intervention protocols, and any procedures for monitoring adherence (eg, drug tablet return; laboratory tests)	11
Interventions: concomitant care	#11d	Relevant concomitant care and interventions that are permitted or prohibited during the trial	9
Outcomes	#12	Primary, secondary, and other outcomes, including the specific measurement variable (eg, systolic blood pressure), analysis metric (eg, change from baseline, final value, time to event), method of aggregation (eg, median, proportion), and time point for each outcome. Explanation of the clinical relevance of chosen efficacy and harm outcomes is strongly recommended	12
Participant timeline	#13	Time schedule of enrolment, interventions (including any run-ins and washouts), assessments, and visits for participants. A schematic diagram is highly recommended (see Figure)	20
Sample size	#14	Estimated number of participants needed to achieve study objectives and how it was determined, including clinical and statistical assumptions supporting any sample size calculations	13
Recruitment	#15	Strategies for achieving adequate participant enrolment to reach target sample size	13
**Methods: Assignment of interventions (for controlled trials)**			
Allocation: sequence generation	#16a	Method of generating the allocation sequence (eg, computer-generated random numbers), and list of any factors for stratification. To reduce predictability of a random sequence, details of any planned restriction (eg, blocking) should be	9

For peer review only

		provided in a separate document that is unavailable to those who enrol participants or assign interventions	
Allocation concealment mechanism	#16b	Mechanism of implementing the allocation sequence (eg, central telephone; sequentially numbered, opaque, sealed envelopes), describing any steps to conceal the sequence until interventions are assigned	9
Allocation: implementation	#16c	Who will generate the allocation sequence, who will enrol participants, and who will assign participants to interventions	9
Blinding (masking)	#17a	Who will be blinded after assignment to interventions (eg, trial participants, care providers, outcome assessors, data analysts), and how	11
Blinding (masking): emergency unblinding	#17b	If blinded, circumstances under which unblinding is permissible, and procedure for revealing a participant's allocated intervention during the trial	n/a, not blinded
**Methods: Data collection, management, and analysis**			
Data collection plan	#18a	Plans for assessment and collection of outcome, baseline, and other trial data, including any related processes to promote data quality (eg, duplicate measurements, training of assessors) and a description of study instruments (eg, questionnaires, laboratory tests) along with their reliability and validity, if known. Reference to where data collection forms can be found, if not in the protocol	11
Data collection plan: retention	#18b	Plans to promote participant retention and complete follow-up, including list of any outcome data to be collected for participants who discontinue or deviate from intervention protocols	11
Data management	#19	Plans for data entry, coding, security, and storage, including any related processes to promote data quality (eg, double data entry; range checks for data values). Reference to where details of data management procedures can be found, if not in the protocol	11
Statistics: outcomes	#20a	Statistical methods for analysing primary and secondary	13

For peer review only

		outcomes. Reference to where other details of the statistical analysis plan can be found, if not in the protocol	
Statistics: additional analyses	#20b	Methods for any additional analyses (eg, subgroup and adjusted analyses)	13
Statistics: analysis population and missing data	#20c	Definition of analysis population relating to protocol non-adherence (eg, as randomised analysis), and any statistical methods to handle missing data (eg, multiple imputation)	13
**Methods: Monitoring**			
Data monitoring: formal committee	#21a	Composition of data monitoring committee (DMC); summary of its role and reporting structure; statement of whether it is independent from the sponsor and competing interests; and reference to where further details about its charter can be found, if not in the protocol. Alternatively, an explanation of why a DMC is not needed	12
Data monitoring: interim analysis	#21b	Description of any interim analyses and stopping guidelines, including who will have access to these interim results and make the final decision to terminate the trial	n/a, not intended
Harms	#22	Plans for collecting, assessing, reporting, and managing solicited and spontaneously reported adverse events and other unintended effects of trial interventions or trial conduct	13
Auditing	#23	Frequency and procedures for auditing trial conduct, if any, and whether the process will be independent from investigators and the sponsor	12
**Ethics and dissemination**			
Research ethics approval	#24	Plans for seeking research ethics committee / institutional review board (REC / IRB) approval	8
Protocol amendments	#25	Plans for communicating important protocol modifications (eg, changes to eligibility criteria, outcomes, analyses) to relevant parties (eg, investigators, REC / IRBs, trial participants, trial registries, journals, regulators)	8
Consent or assent	#26a	Who will obtain informed consent or assent from potential trial participants or authorised surrogates, and how (see Item 32)	9

For peer review only

Consent or assent: ancillary studies	#26b	Additional consent provisions for collection and use of participant data and biological specimens in ancillary studies, if applicable	n/a, no provisions intended
Confidentiality	#27	How personal information about potential and enrolled participants will be collected, shared, and maintained in order to protect confidentiality before, during, and after the trial	11
Declaration of interests	#28	Financial and other competing interests for principal investigators for the overall trial and each study site	14
Data access	#29	Statement of who will have access to the final trial dataset, and disclosure of contractual agreements that limit such access for investigators	11
Ancillary and post trial care	#30	Provisions, if any, for ancillary and post-trial care, and for compensation to those who suffer harm from trial participation	na
Dissemination policy: trial results	#31a	Plans for investigators and sponsor to communicate trial results to participants, healthcare professionals, the public, and other relevant groups (eg, via publication, reporting in results databases, or other data sharing arrangements), including any publication restrictions	13
Dissemination policy: authorship	#31b	Authorship eligibility guidelines and any intended use of professional writers	14
Dissemination policy: reproducible research	#31c	Plans, if any, for granting public access to the full protocol, participant-level dataset, and statistical code	14
**Appendices**			
Informed consent materials	#32	Model consent form and other related documentation given to participants and authorised surrogates	23
Biological specimens	#33	Plans for collection, laboratory evaluation, and storage of biological specimens for genetic or molecular analysis in the current trial and for future use in ancillary studies, if applicable	32

Notes:

- 11b: n/a, not intended
- 17b: n/a, not blinded
- 21b: n/a, not intended

For peer review only

- 26b: n/a, no provisions intended The SPIRIT Explanation and Elaboration paper is distributed under the terms of the Creative Commons Attribution License CC-BY-NC. This checklist was completed on 14. November 2022 using https://www.goodreports.org/, a tool made by the EQUATOR Network in collaboration with Penelope.ai

For peer review only

BMJ Open

Multimodal prehabilitation for major surgery in elderly patients to lower complications: Protocol of a randomised, prospective, multi-centre, multidisciplinary trial (PREHABIL Trial).

Journal:	*BMJ Open*
Manuscript ID	bmjopen-2022-070253.R1
Article Type:	Protocol
Date Submitted by the Author:	08-Dec-2022

Complete List of Authors:	Beilstein, Christian; Inselspital University Hospital Bern, Department of Anaesthesiology and Pain Medicine, Inselspital, Bern University Hospital, University of Bern<br>Krutkyte, Gabija; Inselspital University Hospital Bern, Department of Anaesthesiology and Pain Medicine, Inselspital, Bern University Hospital, University of Bern; Inselspital University Hospital Bern, Division of Diabetes, Endocrinology, Nutritional Medicine and Metabolism, University of Bern<br>Vetsch, Thomas; Inselspital University Hospital Bern, Department of Anaesthesiology and Pain Medicine, Inselspital, Bern University Hospital, University of Bern; Inselspital University Hospital Bern Department of Cardiology, Division of Cardiovascular Prevention, Rehabilitation and Sports Cardiology, University Clinic for Cardiology, Inselspital, Bern University Hospital, University of Bern<br>Eser, Prisca; Inselspital University Hospital Bern Department of Cardiology, Division of Cardiovascular Prevention, Rehabilitation and Sports Cardiology, University Clinic for Cardiology, Inselspital, Bern University Hospital, University of Bern<br>Wilhelm, M; Inselspital University Hospital Bern Department of Cardiology, Division of Cardiovascular Prevention, Rehabilitation and Sports Cardiology, University Clinic for Cardiology, Inselspital, Bern University Hospital, University of Bern<br>Stanga, Zeno; Inselspital University Hospital Bern, Division of Diabetes, Endocrinology, Nutritional Medicine and Metabolism, Bern University Hospital, University of Bern<br>Bally, Lia; Inselspital University Hospital Bern, Department of Diabetes, Endocrinology, Clinical Nutrition and Metabolism<br>Verra, Martin; Inselspital University Hospital Bern, Institute of Physiotherapy, Bern University Hospital, University of Bern<br>Huber, Markus; Inselspital University Hospital Bern, Department of Anaesthesiology and Pain Medicine, Inselspital, Bern University Hospital, University of Bern<br>Wuethrich, Patrick; Inselspital University Hospital Bern, Department of Anaesthesiology and Pain Medicine, Inselspital, Bern University Hospital, University of Bern<br>Engel, Dominique; Inselspital University Hospital Bern, Department of Anaesthesiology and Pain Medicine, Inselspital, Bern University Hospital, University of Bern

**Primary Subject Heading**:	Anaesthesia
Secondary Subject Heading:	Sports and exercise medicine, Geriatric medicine, Surgery
Keywords:	SURGERY, Clinical trials < THERAPEUTICS, GERIATRIC MEDICINE

SCHOLARONE™
Manuscripts

Multimodal prehabilitation for major surgery in elderly patients to lower complications: Protocol of a randomised, prospective, multi-centre, multidisciplinary trial (PREHABIL Trial).

Beilstein CM[1], Krutkyte G[1,3], Vetsch T[1], Eser P[2], Wilhelm M[2], Stanga Z[3], Bally L[3], Verra ML[4], Huber M[1] Wuethrich PY[1], Engel D[1*]

1 Department of Anaesthesiology and Pain Medicine, Inselspital, Bern University Hospital, University of Bern, Switzerland
2 Division of Cardiovascular Prevention, Rehabilitation and Sports Cardiology, University Clinic for Cardiology, Inselspital, Bern University Hospital, University of Bern, Switzerland
3 Division of Diabetes, Endocrinology, Nutritional Medicine and Metabolism, Bern University Hospital, University of Bern, Switzerland
4 Institute of Physiotherapy, Bern University Hospital, University of Bern, Switzerland
*Corresponding author: Dominique.engel@insel.ch

For peer review only

For peer review only - http://bmjopen.bmj.com/site/about/guidelines.xhtml

Abstract

Introduction: The global volume of surgery is growing and the population ageing, and economic pressure is rising. Major surgery is associated with relevant morbidity and mortality. Postoperative reduction in physiological and functional capacity is especially marked in the elderly, multi-morbid patient with low fitness level, sarcopenia and malnutrition. Interventions aiming to optimise the patient prior to surgery (prehabilitation) may reduce post-operative complications and consequently reduce health costs.

Methods and analysis: This is a multi-centre, multidisciplinary, prospective, 2-arm parallel-group, randomised, controlled trial with blinded outcome assessment. Primary outcome is the comprehensive complications index (CCI) at 30 days. Within 3 years, we aim to include 2x233 patients with a proven fitness deficit undergoing major surgery to be randomised using a using computer-generated random numbers and a minimisation technique. The study intervention consists of a structured, multimodal, multidisciplinary prehabilitation program over 2 – 4 weeks addressing deficits in physical fitness and nutrition, diabetes control as well as correction of anaemia and smoking cessation versus standard of care.

Ethics and dissemination: The PREHABIL trial has been approved by the responsible ethics committee (Kantonale Ethikkomission Bern , project ID 2020-01690). All participants provide written informant consent prior to participation. Participant recruitment began in February 2022 (10 & 8 patients analysed at time of submission), with anticipated completion in 2025. Publication of the results in peer reviewed scientific journals are expected in late 2025.

Trial registration number: ClinicalTrials.gov Identifier: NCT04461301

Funding: Swiss National Science Foundation grant number FNSNF 33IC30_201298 / 1 (June 24 2021)

Protocol version: V.1, 01.11.2020

Strengths and limitations of this study:

- This study focuses on frail, elderly patients undergoing major surgery with a proven low functional capacity using cardiopulmonary exercise testing (CPET)
- It implements a structured, multidisciplinary, multimodal prehabilitation intervention addressing low functional capacity, malnutrition, anaemia and smoking.
- The primary endpoint is the comprehensive complication index at 30 days
- Patient reported outcomes such as Quality of Recovery are implemented as secondary outcomes.
- The study is not blinded due to the nature of the intervention, however primary outcome is assessed by a blinded adjudication committee.

For peer review only - http://bmjopen.bmj.com/site/about/guidelines.xhtml

Introduction

Background

The global volume of surgery has grown by about a third, from 234 million procedures in 2008 to more than 312 million in 2012, with most surgeries being performed in developed countries.[1 2] Mortality rates are estimated to be between 1-4% in Europe.[3] A minority of patients at high-risk seem responsible for the majority of complications and mortality.[4] It is therefore of key importance to identify patients at risk preoperatively for several reasons: it allows informed decision-making, it offers the opportunity for preoperative optimisation and it identifies patients with increased intra- and postoperative monitoring needs.

Risk prediction before major surgery

Perioperative risk is largely determined by three domains: urgency of surgery, surgery-specific risk factors such as invasiveness, expected blood loss or fluid shifts, and patient-specific risks such as age, comorbidities and fitness. Whereas the first domain is usually non-modifiable, the latter two are modifiable to some extent, offering the opportunity to adjust the surgical procedure or optimise the patient's condition prior to surgery.

Several pre- or perioperative risk scores and prediction models have been proposed and validated, such as the Portsmouth Physiological and Operative Severity Score for the enumeration of Mortality and morbidity (P-)POSSUM, Surgical Risk Score, the American College of Surgeons National Surgical Quality Improvement Program (NSQIP-ACS) or the Preoperative Score to Predict Postoperative Mortality (POSPOM) for non-cardiac surgery, or the European System for Cardiac Operative Risk (EuroScore) for cardiac surgery.[5-9] In addition, organ specific risk scores such as Revised Cardiac Risk Index for cardiac complications and many others have been published and widely used.[10 11]

Traditionally, patients' reported health state, medical history and clinical assessment were used to classify the patients' individual risk, e. g. using the American Society of Anesthesiologists [12] score. Accordingly, level of fitness was reported by the patients themselves. Based on the 2022 ESC/ESA guidelines for example, further investigations such as (cardiac) stress testing are indicated for patients with low functional capacity (less than 4 metabolic equivalents of task ((MET)) before high-risk elective non-cardiac surgery (NCS) and high likelihood or proven coronary artery disease. Alternatively, before intermediate-risk NCS when ischaemia is of concern in patients with clinical risk factors and poor functional capacity[13].

Patient reported measures of functional capacity remain notoriously unreliable. Better performance seen when using the Duke Activity Status Index (DASI) questionnaire, which correlated well with objective, cardiopulmonary exercise testing (CPET)-derived parameters such as peak oxygen consumption ($VO2_{peak}$) and was able to predict cardiovascular events in a recent trial.[14] While CPET remains the gold standard for global assessment of functional capacity, it is laborious for the patient and costly. Therefore, the Canadian Cardiovascular Society recommends against performing perioperative CPET to enhance cardiac risk estimation.[15] Nevertheless, an anaerobic threshold (AT) level of <11 ml kg^{-1} min^{-1} has been confirmed as a reliable predictor for increased perioperative risk.[16 17] This also applies to the elderly patient.[18] In addition, the AT has the better discriminatory power to identify patients with reduced functional capacity comparted to the 6-minutes walking test (6MWT), which is recommended for departments lacking CPET equipment.[19] Very recent evidence found that an increased VE/VCO2 slope might be an even more sophisticated prognostic marker to predict morbidity in high risk patients having major cancer surgery or patients undergoing lobectomy for lung cancer.[20 21]

For peer review only - http://bmjopen.bmj.com/site/about/guidelines.xhtml

With increasing age, the physiological resilience to external stressors such as surgery diminishes, while the number of comorbidities rises and muscle mass decrease. In addition, elderly people are often malnourished, have a weakened immune system and impaired wound healing. All these factors contribute to a reduction in functional capacity.[22] Despite the inconsistency of the definition of frailty, it can be assessed using a variety of tools such as the clinical frailty scale or more objective Fried frailty criteria including gait speed and handgrip strength, which are useful predictors of all-cause mortality and cardiovascular disease and death in elderly, surgical and non-surgical patients.[23-25] Similarly, standardised screening and assessments exist for the diagnosis of malnutrition and sarcopenia, which demonstrated significant overlap with frailty.[26-28] In summary, the elderly, frail, malnourished and sarcopenic patient is especially at risk for perioperative complications, however, many options for medical, physiological and psychological optimisation exist.[29]

Interventions to reduce perioperative risk

Organ specific, medically mandated interventions (as brought up by the preoperative assessment before treatment of relevant aortic or coronary stenosis) have to be completed prior to major surgery, irrespective of any study participation. However, despite clear guidelines for example on patient blood management, relevant anaemia is not always corrected. In fact, less than half of Swiss hospitals had implemented a patient blood management program in the year 2022.[30]

To empower the patient physically and psychologically to withstand the stress of surgery and to hasten recovery, the focus is widening from the intra- and postoperative to the perioperative period as a continuum. In addition, patients are believed to be more receptive for beneficial changes in behaviour prior to surgery than during the phase of recovery. Whereas rehabilitation is covering the postoperative period, the term prehabilitation has emerged for interventions occurring weeks before surgery.

As of today, prehabilitation is a complex approach with a combination of possible interventions (e.g. exercise, nutrition, psychosocial, medical optimization), and there is no precise definition of the type of intervention, the optimal dose nor duration required. This has led to widely differing interpretations and protocols of interventional trials with unimodal (addressing only one domain such as physical fitness, whereby also mixed-exercise programmes including aerobic, resistance and inspiratory muscles training were implemented) or multimodal (addressing additional domains such as anaemia, malnutrition, smoking behaviour and psychological support) interventions.

Many conflicting results for clinical effectiveness of prehabilitation interventions have been reported.[31-34] The potential for cost reduction seems plausible, but good evidence from randomized controlled trials is lacking so far.[35-38]

Potential reasons for these equivocal results are several fold, with the first and most important probably being inappropriate patient selection. From 8 randomised trials addressing prehabilitation before major intra-abdominal cancer surgery, only 2 limited the study population to "high risk" patients, based on age, ASA or Dukes classification, however, none of them used objectively measured physical fitness as an inclusion criteria.[39] Selecting patients with a relatively low risk for perioperative complications will inevitably lead to a dilution of the possible positive effects and produce negative results regardless of the intervention or surgeries studied. The small or inexistent benefit to “low-risk” patients does not warrant the time- and resource-demanding interventions. In addition, the time required for the intervention has been found to make recruitment of large cohorts difficult.[40 41]

For peer review only - http://bmjopen.bmj.com/site/about/guidelines.xhtml

Secondly, poor results may have been due to implementing unimodal rather than individually tailored multi-modal interventions that cover all domains of patients' health state prior to surgery, such as physical fitness, malnutrition, diabetes control, anaemia, smoking and psychological well-being.

Insufficient exercise capacity is believed to be the most important predictor for postoperative complications[42] and therefore mandates careful assessment with appropriate patient selection. While physical fitness interventions (even high-intensity/interval training) have been proven to be safe and feasible and have led to a measurably increased fitness levels (e.g. 6MWT), they failed to produce outcome benefits, possibly due to inappropriate patient selection, unimodality (e.g. lack of considerations of other factors such as malnutrition) and adherence.[43-46]

Further, up to one third of surgical patients are malnourished, and malnutrition is proven to be associated with increased morbidity, mortality and cost.[47 48] For malnourished patients, oral nutritional supplementation, in particular adequate protein supplementation, has shown to be effective in improving outcomes.[49-51] Malnutrition is often associated with sarcopenia and frailty, making a combined intervention of exercise training and nutritional support promising.[22 45 52] However, so far, nutritional assessment and intervention have been poorly standardised, making conclusions regarding its efficacy and inter-study comparisons difficult.[53]

A further frequent metabolic comorbidity is diabetes affecting approximately 30-40% of people undergoing elective surgery. Surgical outcomes are worse in people with diabetes with an up to three-fold higher risk of postoperative complications including poor healing, wound complications, and renal dysfunction. Although inadequate glucose control was associated with poor surgical outcomes[54], no systematic strategies to improve diabetes management prior to surgery are currently recommended and only few pre-habilitation programs included diabetes optimisation as part of a multi-modal concept.

Anaemia-related perioperative blood transfusions and smoking have both been found to be related to worse outcomes and increased costs.[55-57]

The negative influence of pre-operative anxiety or depression on postoperative outcomes such as quality of life have gained more attention.[58 59] Even without an proper psychosocial intervention, physical exercise can reduce psychological distress in addition to improving cardiovascular function and ultimately improve wound healing.[60]

Another factor contributing to negative results of existing multimodal prehabilitation programs might be attributable to the simple fact that patients are randomized into the intervention group, but are not taking actively part in the program (lack of patient adherence)[61]. Some studies fail to demonstrate that effect by not measuring adherence rates at all[12]

So the necessity of implementing a multimodal intervention seems obvious given the important contribution of anaemia, smoking, nutritional status, physical fitness and psychological well-being. Nevertheless, even for multimodal prehabilitation, the level of evidence remains low and conflicting, with inappropriate patient selection appearing to be the main reason.[32 33]

In conclusion, no trials to date have limited the study population to "high-risk" patients most prone to benefit from prehabilitation. Further, only few studies have implemented a multimodal approach targeting correction of anaemia and malnutrition, smoking cessation and improving physical fitness. There is a need for trials with stringent selection of the patients who will benefit most from preoperative optimisation and

For peer review only - http://bmjopen.bmj.com/site/about/guidelines.xhtml

implementation of personalised comorbidity mitigation and risk factor management offered in the form of individually tailored multimodal prehabilitation.[62]

Outcome measures after major surgery

The most widely accepted postoperative hard outcome measure is mortality at 30 days.[6] Nevertheless, extending the follow-up to at least 90 days or ideally 1 year has been proposed.[63] To grade postoperative complications, the Clavien-Dindo classification has been established and recommended by the ESA-ESICM joint taskforce on perioperative outcome measures.[63-65] There is a huge variety of possible postoperative complications, and powering studies for individual complications is nearly impossible. To overcome this difficulty, the comprehensive complication index (CCI) has been developed.[66] It is a continuous scale to measure surgical morbidity (ranging from 0= no complications to 100 = death) and can be used to reduce sample size in randomised controlled trials as it provides a more sensitive endpoint.[67-69]

Calculation of cost savings

The CCI be used to calculate costs, as postoperative morbidity correlates highly with health care costs. Staiger et al. found a 14% increase to the baseline cost for each 10-point increase in CCI.[70] Therefore, a CCI-reduction can serve as a surrogate marker for cost-savings.

Study hypothesis

We hypothesise that the implementation of a multimodal, multidisciplinary prehabilitation program over two to four weeks, consisting of physical exercise, nutrition, anaemia correction and smoking cessation, will result in lower CCI within 30 days after major surgery in elderly patients.

Feasibility: Preliminary Data

After ethics approval and logistic preparations, we were able to include 18 patients from 03/2022 until 10/2022 (Table 1).

Table 1: Preliminary Results

	**Control Group (n=8)**	**Intervention Group (n=10)**
**Demographics / Comorbidities**		
Age (years)	78.25 (SD 5.4)	78.20 (SD 5.5)
Female Sex	2 (25%)	1 (10%)
ASA Score = III (n)	2 (25%)	2 (20%)
ASA Score ≥ IV (n)	6 (75%)	8 (80%)
Fried Frailty Scale (n)	1.9 (SD 1.1)	2.1 (SD 1.2)
Smoking (y/n)	1 (12.5%)	0 (0%)
Anaemia (y/n)	2 (25%)	4 (40%)
Nutritional Deficit (NRS-2002 ≥3) (y/n)	2 (25%)	1 (10%)

For peer review only - http://bmjopen.bmj.com/site/about/guidelines.xhtml

Ischaemic Heart Disease or Chronic Heart Failure (y/n)	4 (50%)	6 (60%)
Diabetes (y/n)	3 (37.5%)	3 (30%)
Obesity (BMI ≥30 kg/m^2) (y/n)	1 (12.5%)	1 (10%)
**Surgical Procedure**		
Cardiac<br>Orthopaedic<br>Thoracic<br>Urologic<br>Vascular<br>Visceral	2<br>0<br>0<br>3<br>2<br>1	3<br>1<br>1<br>4<br>1<br>0
Use of ECC (y/n)	2 (25%)	3 (30%)
P-POSSUM (baseline)	43.3 (SD 6.5)	45.2 (SD 8.2)
Days to surgery (days)	29.8 (SD 19.7)	28.5 (SD 9.3)
**Laboratory Parameters (baseline)**		
Haemoglobin (g/L)	136.8 (SD 16.3)	126.9 (SD 13.8)
NT-proBNP (pg/mL)	3677.4 (SD 5022.3)	886.8 (SD 881.5)
Albumin (g/L)	33.3 (SD 3.9)	36.0 (SD 2.3)
Prealbumin (g/L)	0.22 (SD 0.04)	0.25 (SD 0.06)
HbA1c (%)	6.0 (SD 0.7)	6.4 (SD 1.4)
Creatinine (mmol/L)	147.9 (SD 51.5)	131.0 (SD 53.4)
**Fitness**		
MIP (cmH20)	51.3 (SD 28.0)	46.9 (SD 16.1)
AT (ml/min/kg)	9.8 (SD 1.0)	9.1 (SD 1.3)
VO2peak (ml/min/kg)	12.7 (SD 1.4)	11.8 (SD 2.8)
**Questionnaires**		
STAI (n)	10.6 (SD 2.4)	13 (SD3.9)
DASI (n)	4.1 (SD 0.8)	4.6 (SD 1.4)

For peer review only

For peer review only - http://bmjopen.bmj.com/site/about/guidelines.xhtml

ASA = American Society of Anesthesiologists, NRS-2002 = Nutritional Risk Screening 2002, AT = Anaerobic Threshold, BMI = Body Mass Index, DASI = Duke Activity Status Index, ECC = Extracorporeal Circulation, MIP = Maximum Inspiratory Pressure, STAI = State-Trait Anxiety Inventory

Methods

Study design

This is a national multi-centre, multidisciplinary, prospective, 2-arm parallel-group, randomised, controlled superiority trial with blinded outcome assessment. We aim to include 466 patients within 3 years. The present protocol complies with the SPIRIT 2013 guidelines, defining standard protocol items for clinical trials.

Participants and enrolment

Surgeons scheduling the operation will pre-screen the patients for frailty based on the clinical frailty scale.[71] This tool is easy to implement in clinical practice, especially when compared to other, more sophisticated scales.[72] If the patient achieves at least 4 points on the clinical frailty scale (living with very mild frailty) and there is sufficient time for prehabilitation (at least 2 weeks), informed consent will be sought, followed by a screening CPET (baseline visit, see Figure 1).

Inclusion criteria

Participants are eligible if aged 65 years or over, have planned major visceral, urologic, cardiac, vascular, orthopaedic or thoracic surgery, are classified as ASA III or higher by the attending anaesthetist, and have a proven deficit in functional capacity measured during the screening CPET defined as follows:

- VO2 at AT < 11 ml/kg/min or peak VO2< 14 ml/kg/min on cycle ergometer
- VO2 at AT < 13 ml/kg/min or peak VO2 < 16.5 ml/kg/min on treadmill
- VO2 at AT < 9 ml/kg/min or peak VO2 < 11.3 ml/kg/min with arm crank
- and/or VE/VCO2 slope ≥33 (independent of modality)

Patients with VO2 above thresholds during the screening CPET will nevertheless be consented, in order to collect their CCI at 30 days. CCI at 30 days will be compared descriptively between patients qualifying and not qualifying for this study for qualitative clinical assessment outside the purpose of this study.

Exclusion criteria

Patients are excluded if scheduled for emergency surgery, if unable to exercise for physiological or psychological reasons, if having cognitive disabilities making adherence to interventions impossible or if requiring dialysis due to logistic reasons.

Preoperative assessments

All patients are assessed twice during the preoperative phase, once at inclusion (baseline) and once the day before surgery (preoperative). Measurements conducted during these visits include CPET, grip strength and maximal inspiratory pressure for the assessment of physical fitness. Bio-electrical impedance analysis [13] measurements and validated nutritional screening and assessment tools (Nutrition Risk Screening 2002 (NRS-2002) and the Subjective Global Assessment (SGA)) will be performed to quantify participants' body composition and nutritional status. Blood samples are taken to screen for anaemia (haemoglobin, reticulocyte index, renal function, iron status using transferrin saturation), nutritional and diabetic status (albumin, prealbumin, glucose, HbA1c), micronutrient deficits (erythrocyte folate, holotranscobalamin, 25-hydroxy-vitamin D) and cardiac function (NT-proBNP, hsTroponinT). For smokers,

For peer review only - http://bmjopen.bmj.com/site/about/guidelines.xhtml

For peer review only

exhaled CO is measured (Smokerlyzer® Bedfont® Scientific Ltd, Kent, UK) and smoking habits evaluated by questionnaire including the Fagerström test. All patients are asked to fill in the short form of the State-Trait Anxiety Inventory [70], the DASI and (on the day prior to operation) the Quality of Recovery (QoR-)15 questionnaire. An activity tracker (Axivity® Accelerometer, Axivity Ltd, Newcastle, United Kingdom) is provided at the screening visit and continuously worn until the preoperative visit, when it is collected from the patient.

Group allocation (minimisation)

After completion of the screening visit (CPET), patients who fulfil the inclusion criteria are randomised using computer-generated random numbers, into either intervention or control group. For group allocation, a probabilistic minimisation procedure is used to achieve balanced groups in regards to parameters known to be relevant for outcome after major surgery. We have included the following parameters in the minimisation algorithm (SecuTrial, Version 6.1.1.10): type of surgery (visceral, urologic, cardiovascular, orthopaedic, thoracic), smoking, presence of anaemia (haemoglobin below 120 g/L in females, < 130 g/L in males), nutritional deficit (NRS ≥3), age group (65-74, 75-84, 85+), ischaemic heart disease, known diabetes, sex, obesity (body mass index >30 kg/m^2), and use of extracorporeal circulation.

Patient and Public involvement

Patients or the public were not involved in the design, or conduct, or reporting, or dissemination plans of our research.

Study interventions

Control group

For the control group, there is a medical work up by the study team of the results obtained. In case of abnormalities found mandating medical interventions prior to surgery based on clinical judgement, these will be initiated either by the research team or the general practitioner (GP) at the patients' discretion. Regardless of existing deficits, the patient's GP will be informed about the patient's study participation and will receive the results of the screening assessment. All study patients wear the accelerometer between screening and preoperative visit.

Intervention group

For patients of the intervention group, we will implement an individually tailored, multimodal prehabilitation program consisting of physical and respiratory training, correction of nutritional deficits and anaemia and smoking cessation counselling based on the identified deficits.

Physical activity

All patients perform a tailored exercise intervention based on their individual physical fitness level. The intervention includes 1 to 2 sessions (60 minutes each) with a licensed physical therapist trained in exercise prescription and 5 years of experience in the field of exercise prescription. The physical therapist will assess the patient's physical abilities and discuss the possibilities of incorporating at least 30 minutes of exercise at least 5 times per week at home. Based on the planned surgical intervention (e.g. hip, back, etc.), the preferences and barriers of each individual patient, the physical therapist composes a tailored personal training programme. This physical training program is composed of endurance training (e.g. walking at moderate intensity, climbing stairs), resistance training (e.g. upper limb strengthening using a resistance band, Theraband, Akron, Ohio, USA) and inspiratory muscle training (Threshold IMT, Philips Healthcare, Horgen, Switzerland). The patient receives the personal training programme either via smartphone

For peer review only - http://bmjopen.bmj.com/site/about/guidelines.xhtml

application or as a hard copy. The non-supervised home-based exercise training sessions will be supported by illustrative materials (smartphone application with pictures and videos or brochure with pictures). Each patient receives weekly phone calls by the physical therapist to record adherence, as a motivational strategy, to discuss any boundaries to adhering to the programme and to progress the exercises if possible. A exercise intervention might look like the following program: Respiratory: IMT, 40% of measures MIP, 60 repetitions daily (30 morning, 30 evening); Endurance: outdoor walking at moderate intensity, 30 minutes 5 days/week; Strength: Squats with bedside support, 3x10 repetitions, as deep as pain free, 5 days/week.

Nutritional support and management of anaemia

We aim to optimise the nutritional status to minimise the surgery-related catabolic stress. Nutritional risk will be assessed at baseline, preoperatively and postoperatively during study visits using the NRS-2002 total score.[73] This validated screening tool attributes scores of different level of nutritional status to patients based on weight loss, body mass index (BMI) and food intake, taking into account disease severity (degree of stress metabolism) and age. NRS-2002 total score is obtained by adding impaired nutritional status score (0-3 points), severity of disease score (0-3 points), and age (1 point if age ≥ 70 years). Patients are classified as being at high nutritional risk or malnourished (score ≥3) or at low risk (score <3), according to the total score obtained (see figure 2).

After screening with NRS-2002, nutritional status will be further quantified according to the PG-SGA score, which defines the need for nutritional intervention: no intervention needed (score 0-1), needing education by a professional (score 2-3), requiring intervention by a certified dietitian (score 4-8) and having a critical need for symptom management and/or nutrient intervention (score ≥9).[74]

Patients with low risk for malnutrition (NRS-2002 < 3) will be provided with dietary advice on how to achieve set individual nutritional targets (energy intake according to the Harris-Benedict equation and protein intake of >1 g/kg BW/day). Adjusted bodyweight will be used in case of BMI > 25 to prevent overestimation of nutritional requirements.

Patients at high risk for being malnourished (NRS-2002 ≥3, PG-SGA ≥4) will receive dietary counselling and individualised plans to meet their energy and protein requirements. Individual energy needs will be calculated using Harris-Benedict equation. A protein intake of 1.2-1.5 g/kg BW will be recommended to all malnourished participants (using adjusted body weight when BMI>25, as described above). To facilitate the achievement of these goals, oral nutritional supplements will be prescribed. The aim is to achieve at least 75% of the set energy and protein goal. The adherence in malnourished group will be checked by performing 24h dietary recall 3 days after baseline visit. In case of reaching <75% of set dietary goals, the intervention will be escalated to enteral nutrition. If the goals are not fulfilled during the following three days as evaluated with 24h dietary recall, the intervention will be escalated to parenteral nutrition.

If scheduled for major abdominal surgery and if indication exists according to Swiss Society for Clinical Nutrition, patients will additionally receive immunonutrition (e.g. high protein oral supplements enriched with immunonutrients such as arginine, omega-3-fatty acids and nucleotides) for five consecutive days before surgery.[75 76] , as per ERAS guidelines. All participants will receive 2 doses of carbohydrate loading, one dose on the evening before and one up to 2 hours prior to surgery.[76]

In patients with an established or newly diagnosed diabetes and HbA1c>7.5%, glucose levels will be optimised using pre-scribed glucose monitoring and treatment intensification where appropriate. Delivered strategies represent best practice of diabetes care based on the EASD-ADA position statement.[77]

For peer review only - http://bmjopen.bmj.com/site/about/guidelines.xhtml

Independent of the nutritional status, individual deficits in vitamin D, iron, vitamin B12 and folate will be addressed. Vitamin D is associated with muscle mass and muscle strength. Additionally, hypovitaminosis D was found to be correlating with increased risk of postoperative complications.[78 79] It will be supplemented daily according to the blood sample values: 1000 U/d for patients with vitamin D deficiency (25-hydroxy-vitamin D <50 nmol/l) and a one-off substitution of 150 000 U for patients with severe vitamin D deficiency (25-hydroxy-vitamin D < 25 nmol/l).[80]

In case of severe renal anaemia (haemoglobin < 100 g/L, eGFR < 45 ml/min/1.73m^2 and a reticulocyte index below 2), a one-off erythropoietin application (Mircera®, Vifor Pharma, Switzerland) will be provided.

Smoking cessation

The smoking cessation program includes intensive counselling by an advanced psychology student during three phone-call sessions as well as dispensary of nicotine replacement therapy (NRT) perioperatively if desired by the patient. NRT consist of transdermal application for moderate to heavy smokers and individual supplementation with chewing gum or lozenges (Nicotinell®, GlaxoSmithKline, Brentford, United Kingdom) according to the current guidelines. NRT will either be handed out during the nutritional intervention session or sent via postal mail. The goal is to achieve a reduction in smoking quantity (cigarettes per day) of 80% before surgery.

Compliance with the study interventions

Adherence rates to the physical fitness intervention are primarily collected by patient-recorded standardised physical activity log books, by via weekly phone calls and completed by accelerometer data (Axivity AX3, Axivity Ltd, Newcastle, UK). Successful completion of the exercise program is defined as adherence to 80% of prescribed exercise sessions.

For patients with a NRS-2002 ≥3 or PG-SGA ≥4, weekly phone calls by the dietician will secure adherence to the study intervention. In addition, the patients will document the intake of prescribed supplements in a log book.

The 3 phone call smoking cessation counselling sessions for smokers are intended to monitor and further adherence to smoking cessation. Further, exhaled CO is measured at every visit.

Reasons for non-compliance are recorded during all phone calls and strategies to increase compliance are sought in the discussion with the patient.

Perioperative management

Perioperative management follows the usual care of the corresponding clinic, usually following standardised, multi-element ERAS guidelines. Management decisions such as extension of monitoring and mission to postoperative care (postoperative care unit versus intermediate or intensive care unit) are purely based on clinical judgement, taking the results of the pre-operative testing into account as it is accessible by the clinicians trough the electronic health care record [57] system.

In case of raised preoperative NTproBNP >200 pg/ml, hsTroponinT will be measured 6 hours after surgery as well as on the first and second postoperative day.[81]

For peer review only - http://bmjopen.bmj.com/site/about/guidelines.xhtml

Postoperative assessment

The postoperative assessment as described above will be repeated at 30 days after surgery when the patient is scheduled for surgical follow up. The only difference from the preoperative assessment is that micronutrient and iron status are not measured again.

Blinding procedure

This is an unblinded study due to the nature of the intervention. Nevertheless, the treating anaesthetist, while having access to the results of the preoperative assessment results via EHR, is blinded to group allocation. Primary end point adjudication committee will also be blinded (see below).

Data collection

All data will be collected by the research team and documented in a research database (RedCap) according to local legislation. Data will be verified by dedicated members of the research team. Original source data is available in the EHR or in patients` files.

End point adjudication committee

In order to standardise the review process and to reduce bias and variability in assessing the primary outcome, the CCI at 30 days will be assessed by an independent and blinded committee of three experts, which are otherwise not involved in the trial. The experts are yet to be nominated.

Data monitoring

The data will be monitored by an external institution (Clinical Trials Unit, University of Bern) at regular intervals according to a pre-set monitoring plan to ensure the safety of the trial participants and the integrity of the trial data.

Primary endpoint

Postoperative complications at 30 days after surgery scored by the Comprehensive Complication Index (CCI), assessed by a blinded, independent expert committee.

Secondary endpoints

Secondary outcomes are measured before prehabilitation (screening visit) and thereafter (preoperative visit) and at 30 days after surgery (postoperative visit) unless otherwise specified:

Cardiovascular & Pulmonary

- Cardio-Pulmonary Exercise Test (CPET): Peak VO2, VO2 at AT, peak VE, VE/VCO2 slope, Oxygen Uptake Efficiency Slope (OUES), FEV1, FEV1/FVC, resting HR, HR reserve, resting systolic and diastolic BP
- Grip strength
- Physical Function Performance 10 Test (CS-PFP 10)
- CPAx ICUD and CPAX HosD in case of ICU admission
- Maximum inspiratory pressure (MIP)
- NTproBNP
- Serial hs-Troponin-T
- Change in preoperative P-POSSUM score

Nutrition & Bio-Impedance

- Nutrition Risk Screening Score 2002

For peer review only - http://bmjopen.bmj.com/site/about/guidelines.xhtml

- Patient Generated Subjective Global Assessment- Bioelectrical impedance analysis: weight, muscle mass, fat mass, percent body fat, extracellular water/intracellular water, phase angle, Fat Free Body Mass (FFM)
- Days nil per mouth (only at postoperative visit)

Anaemia
- Presence of anaemia
- Transfusion in the first 30 days after surgery

Smoking
- Average number of cigarettes per day, Fagerström-Score
- Exhaled CO

Patient Reported Outcomes
- Quality of Recovery Score (QoR-15)
- State-Trait Anxiety Inventory [70]
- Duke Activity Status Index (DASI)

Other outcomes of interest
- Length of hospital stay
- Days at home at 30 days
- Predicted costs for visceral interventions
- CCI at 30 days assessed by the treating physician (unblinded)
- CCI at 90 days assessed by the treating physician (unblinded)

Safety outcomes
- Days to surgery
- (Serious) adverse events

Sample size

Based on internal data (Bern University Hospital), median CCI is expected to be 30 (IQR 21 to 37). Assuming an average clinically relevant improvement of 15% in the intervention group compared to the control group, the sample size calculation for a non-parametric Wilcoxon-Mann-Whitney test resulted in a total patient sample of 388 (assuming a significance level alpha of 0.05 and power of 0.80). We expect a dropout rate of 20%. We therefore need 466 patients: 233 in each arm. We aim to complete the inclusion within 3 years.

Statistical analysis

Data will be analysed on an intention-to-treat basis (primary outcome). Primary and secondary outcomes for the intervention and control groups will be compared. An interim analysis is planned when half of the intended number of subjects have completed the 4 week assessment (i.e. timing of the primary endpoint assessment).

Primary outcome: We expect that CCI will be skewed, so we will describe CCI as the median plus first and third quartiles. To test the hypothesis (H0) that the study arms result in similar CCIs (in other words, prehabilitation does not prevent postoperative complications), we will use a generalized linear model that can account for skewed data and zero inflation. The model will be adjusted for the minimization factors used at randomization. If no adequate model can be fit, the non-parametric Mann-Whitney U test will be used.

For peer review only - http://bmjopen.bmj.com/site/about/guidelines.xhtml

Secondary outcomes: All secondary outcomes will be described as means plus SD or median plus first and third quartiles for continuous data as appropriate. Categorical parameters will be described as frequencies plus percentage per time point. To account for repeat measurements of patients, we will use generalized linear mixed models to test the hypothesis of both study arms being equal in terms of secondary outcomes over time. Models will be adjusted for minimization factors as well as baseline values (before randomization) of the respective outcome.

Data distribution will be assessed by visual inspection of Q-Q plots.

As a secondary analysis, we will perform a PP analysis. Since the time between randomization and surgery might differ between the two randomization groups, we will additionally adjust generalized linear models of the primary outcome for this factor in a sensitivity analysis. Further, we will analyse the impact of adherence to the interventions to the primary outcome.

Subgroup analysis

We will study whether effects differ between subgroups. Subgroup analyses will be performed according to the minimisation parameters (i.e. surgery type, anaemia, nutritional deficit, age, frailty score, ischaemic heart disease).

Ethics and Dissemination

The trial will be conducted according the current version of the Declaration of Helsinki, ICH-GCP in analogy, and applicable national regulations, i.e., the Human Research Act in Switzerland and has been accepted by the responsible Ethics Committee (REC) of the Canton of Berne, Switzerland (Kantonale Ethikkomission Bern 2020-01690). After initial ethics approval, subsequent protocol amendments will be submitted to the REC. Written informed consent will be obtained from all participants before entering the study.

Meetings and project presentations will be performed at the involved surgical clinics to advertise our project and raise awareness in order to motivate recruitment at regular intervals.

Results of the trial are expected to be published in major medical journals and to be presented at international congresses.

Authorship for the project will be based on the Uniform Requirements for Manuscripts of the International Committee of Medical Journal Editors (http://www.icmje.org/#author). We followed the SPIRIT checklist when writing our report.[82]

Public access to the dataset on patient-level is not planned but possible on reasonable request.

Authors' contributions: The study was initiated by P.Y. Wuethrich and D. Engel. D. Engel is the principal investigator. D. Engel, P.Y. Wuethrich, M. Wilhelm, M. Verra, Z. Stanga, P. Eser and M. Huber designed the study. D. Engel, P.Y. Wuethrich, C. Beilstein, P. Eser, M. Huber, T. Vetsch, G. Krutkyte, M. Verra and L. Bally were involved in the conception of the study and initial draft of the protocol. Logistic organization of the study was done by C. Beilstein, D. Engel, P.Y. Wuethrich, T. Vetsch, G. Krutkyte, P. Eser, M. Wilhelm, M. Verra and L. Bally. All authors were involved in the draft of the article and have critically reviewed and approved the final protocol. P.Y. Wuethrich, D. Engel and C. Beilstein take senior responsibility of all processes.

For peer review only - http://bmjopen.bmj.com/site/about/guidelines.xhtml

Funding: This work is supported by the Schweizerischer Nationalfonds zur Förderung der Wissenschaftlichen Forschung (Swiss National Science Foundation grant number FNSNF 33IC30_201298 / 1 (June 24 2021)), www.snf.ch

Competing interests: None of the authors declares to have a conflict of interest.

For peer review only

For peer review only - http://bmjopen.bmj.com/site/about/guidelines.xhtml

References

1. Weiser TG, Haynes AB, Molina G, et al. Size and distribution of the global volume of surgery in 2012. *Bull World Health Organ* 2016;94(3):201-09f. doi: 10.2471/blt.15.159293 [published Online First: 2016/03/12]
2. Weiser TG, Regenbogen SE, Thompson KD, et al. An estimation of the global volume of surgery: a modelling strategy based on available data. *Lancet* 2008;372(9633):139-44. doi: 10.1016/s0140-6736(08)60878-8 [published Online First: 2008/06/28]
3. Pearse RM, Moreno RP, Bauer P, et al. Mortality after surgery in Europe: a 7 day cohort study. *Lancet* 2012;380(9847):1059-65. doi: 10.1016/s0140-6736(12)61148-9 [published Online First: 2012/09/25]
4. Pearse RM, Harrison DA, James P, et al. Identification and characterisation of the high-risk surgical population in the United Kingdom. *Crit Care* 2006;10(3):R81. doi: 10.1186/cc4928 [published Online First: 2006/06/06]
5. Nashef SA, Roques F, Michel P, et al. European system for cardiac operative risk evaluation (EuroSCORE). *Eur J Cardiothorac Surg* 1999;16(1):9-13. doi: 10.1016/s1010-7940(99)00134-7 [published Online First: 1999/08/24]
6. Brooks MJ, Sutton R, Sarin S. Comparison of Surgical Risk Score, POSSUM and p-POSSUM in higher-risk surgical patients. *Br J Surg* 2005;92(10):1288-92. doi: 10.1002/bjs.5058 [published Online First: 2005/06/28]
7. Le Manach Y, Collins G, Rodseth R, et al. Preoperative Score to Predict Postoperative Mortality (POSPOM): Derivation and Validation. *Anesthesiology* 2016;124(3):570-9. doi: 10.1097/aln.0000000000000972 [published Online First: 2015/12/15]
8. Whiteley MS, Prytherch DR, Higgins B, et al. An evaluation of the POSSUM surgical scoring system. *Br J Surg* 1996;83(6):812-5. doi: 10.1002/bjs.1800830628 [published Online First: 1996/06/01]
9. Bilimoria KY, Liu Y, Paruch JL, et al. Development and Evaluation of the Universal ACS NSQIP Surgical Risk Calculator: A Decision Aid and Informed Consent Tool for Patients and Surgeons. *J Am Coll Surg* 2013;217(5):833-42e3. doi: 10.1016/j.jamcollsurg.2013.07.385
10. Lee TH, Marcantonio ER, Mangione CM, et al. Derivation and prospective validation of a simple index for prediction of cardiac risk of major noncardiac surgery. *Circulation* 1999;100(10):1043-9. doi: 10.1161/01.cir.100.10.1043 [published Online First: 1999/09/08]
11. Bierle DM, Raslau D, Regan DW, et al. Preoperative Evaluation Before Noncardiac Surgery. *Mayo Clin Proc* 2020;95(4):807-22. doi: 10.1016/j.mayocp.2019.04.029 [published Online First: 2019/11/23]
12. Ausania F, Senra P, Melendez R, et al. Prehabilitation in patients undergoing pancreaticoduodenectomy: a randomized controlled trial. *Revista Espanola de Enfermedades Digestivas*;111(8):603-08.
13. Halvorsen S, Mehilli J, Cassese S, et al. 2022 ESC Guidelines on cardiovascular assessment and management of patients undergoing non-cardiac surgery: Developed by the task force for cardiovascular assessment and management of patients undergoing non-cardiac surgery of the European Society of Cardiology (ESC) Endorsed by the European Society of Anaesthesiology and Intensive Care (ESAIC). *European Heart Journal* 2022;43(39):3826-924. doi: 10.1093/eurheartj/ehac270
14. Wijeysundera DN, Pearse RM, Shulman MA, et al. Assessment of functional capacity before major non-cardiac surgery: an international, prospective cohort study. *Lancet* 2018;391(10140):2631-40. doi: 10.1016/s0140-6736(18)31131-0 [published Online First: 2018/08/03]
15. Duceppe E, Parlow J, MacDonald P, et al. Canadian Cardiovascular Society Guidelines on Perioperative Cardiac Risk Assessment and Management for Patients Who Undergo Noncardiac Surgery. *Can J Cardiol* 2017;33(1):17-32. doi: 10.1016/j.cjca.2016.09.008 [published Online First: 2016/11/21]

For peer review only - http://bmjopen.bmj.com/site/about/guidelines.xhtml

16. Snowden CP, Prentis JM, Anderson HL, et al. Submaximal cardiopulmonary exercise testing predicts complications and hospital length of stay in patients undergoing major elective surgery. *Ann Surg* 2010;251(3):535-41. doi: 10.1097/SLA.0b013e3181cf811d [published Online First: 2010/02/06]
17. Wilson RJ, Davies S, Yates D, et al. Impaired functional capacity is associated with all-cause mortality after major elective intra-abdominal surgery. *Br J Anaesth* 2010;105(3):297-303. doi: 10.1093/bja/aeq128 [published Online First: 2010/06/25]
18. Older P, Hall A, Hader R. Cardiopulmonary exercise testing as a screening test for perioperative management of major surgery in the elderly. *Chest* 1999;116(2):355-62. doi: 10.1378/chest.116.2.355 [published Online First: 1999/08/24]
19. Sinclair RC, Batterham AM, Davies S, et al. Validity of the 6 min walk test in prediction of the anaerobic threshold before major non-cardiac surgery. *Br J Anaesth* 2012;108(1):30-5. doi: 10.1093/bja/aer322 [published Online First: 2011/10/08]
20. Kristenson K, Hylander J, Boros M, et al. Ventilatory efficiency in combination with peak oxygen uptake improves risk stratification in patients undergoing lobectomy. *JTCVS Open* 2022;11:317-26. doi: 10.1016/j.xjon.2022.06.018 [published Online First: 2022/09/30]
21. Nawoor-Quinn Z, Oliver A, Raobaikady R, et al. The Marsden Morbidity Index: the derivation and validation of a simple risk index scoring system using cardiopulmonary exercise testing variables to predict morbidity in high-risk patients having major cancer surgery. *Perioperative Medicine* 2022;11(1):48. doi: 10.1186/s13741-022-00279-8
22. Csete ME. Basic Science of Frailty-Biological Mechanisms of Age-Related Sarcopenia. *Anesth Analg* 2021;132(2):293-304. doi: 10.1213/ane.0000000000005096 [published Online First: 2020/08/10]
23. Bohannon RW. Hand-grip dynamometry predicts future outcomes in aging adults. *J Geriatr Phys Ther* 2008;31(1):3-10. doi: 10.1519/00139143-200831010-00002 [published Online First: 2008/05/21]
24. Celis-Morales CA, Welsh P, Lyall DM, et al. Associations of grip strength with cardiovascular, respiratory, and cancer outcomes and all cause mortality: prospective cohort study of half a million UK Biobank participants. *BMJ* 2018;361:k1651. doi: 10.1136/bmj.k1651 [published Online First: 2018/05/10]
25. Chang J, Nathalie J, Nguyenhuy M, et al. Slow gait speed is associated with worse postoperative outcomes in cardiac surgery: A systematic review and meta-analysis. *J Card Surg* 2022;37(1):197-204. doi: 10.1111/jocs.16089 [published Online First: 2021/10/20]
26. Burden ST, Bibby N, Donald K, et al. Nutritional screening in a cancer prehabilitation programme: A cohort study. *J Hum Nutr Diet* 2022 doi: 10.1111/jhn.13057 [published Online First: 2022/07/02]
27. Cruz-Jentoft AJ, Bahat G, Bauer J, et al. Sarcopenia: revised European consensus on definition and diagnosis. *Age Ageing* 2019;48(1):16-31. doi: 10.1093/ageing/afy169 [published Online First: 2018/10/13]
28. Kondrup J, Allison SP, Elia M, et al. ESPEN guidelines for nutrition screening 2002. *Clin Nutr* 2003;22(4):415-21. doi: 10.1016/s0261-5614(03)00098-0 [published Online First: 2003/07/26]
29. Alvarez-Nebreda ML, Bentov N, Urman RD, et al. Recommendations for Preoperative Management of Frailty from the Society for Perioperative Assessment and Quality Improvement (SPAQI). *J Clin Anesth* 2018;47:33-42. doi: 10.1016/j.jclinane.2018.02.011 [published Online First: 2018/03/20]
30. Previsdomini M, Colombo J, Cerutti B, et al. Dissemination of patient blood management practices in Swiss intensive care units: a cross-sectional survey. *Swiss Med Wkly* 2022;152:w30184. doi: 10.4414/smw.2022.w30184 [published Online First: 2022/06/27]
31. Gloor S, Misirlic M, Frei-Lanter C, et al. Prehabilitation in patients undergoing colorectal surgery fails to confer reduction in overall morbidity: results of a single-center, blinded, randomized controlled trial. *Langenbecks Arch Surg* 2022 doi: 10.1007/s00423-022-02449-0 [published Online First: 2022/01/28]
32. Carli F, Bousquet-Dion G, Awasthi R, et al. Effect of Multimodal Prehabilitation vs Postoperative Rehabilitation on 30-Day Postoperative Complications for Frail Patients Undergoing Resection of

For peer review only - http://bmjopen.bmj.com/site/about/guidelines.xhtml

Colorectal Cancer: A Randomized Clinical Trial. *JAMA Surg* 2020;155(3):233-42. doi: 10.1001/jamasurg.2019.5474 [published Online First: 2020/01/23]
33. Perry R, Herbert G, Atkinson C, et al. Pre-admission interventions (prehabilitation) to improve outcome after major elective surgery: a systematic review and meta-analysis. *BMJ Open* 2021;11(9):e050806. doi: 10.1136/bmjopen-2021-050806 [published Online First: 2021/10/02]
34. Yau DKW, Underwood MJ, Joynt GM, et al. Effect of preparative rehabilitation on recovery after cardiac surgery: A systematic review. *Ann Phys Rehabil Med* 2021;64(2):101391. doi: 10.1016/j.rehab.2020.03.014 [published Online First: 2020/05/25]
35. Koh FH, Loh CH, Tan WJ, et al. Structured presurgery prehabilitation for aged patients undergoing elective surgery significantly improves surgical outcomes and reduces cost: A nonrandomized sequential comparative prospective cohort study. *Nutr Clin Pract* 2022;37(3):645-53. doi: 10.1002/ncp.10787 [published Online First: 2021/12/04]
36. Nielsen PR, Andreasen J, Asmussen M, et al. Costs and quality of life for prehabilitation and early rehabilitation after surgery of the lumbar spine. *BMC Health Serv Res* 2008;8:209. doi: 10.1186/1472-6963-8-209 [published Online First: 2008/10/10]
37. Ploussard G, Almeras C, Beauval JB, et al. A combination of enhanced recovery after surgery and prehabilitation pathways improves perioperative outcomes and costs for robotic radical prostatectomy. *Cancer* 2020;126(18):4148-55. doi: 10.1002/cncr.33061 [published Online First: 2020/07/09]
38. Barberan-Garcia A, Ubre M, Pascual-Argente N, et al. Post-discharge impact and cost-consequence analysis of prehabilitation in high-risk patients undergoing major abdominal surgery: secondary results from a randomised controlled trial. *Br J Anaesth* 2019;123(4):450-56. doi: 10.1016/j.bja.2019.05.032 [published Online First: 2019/06/30]
39. Thomas G, Tahir MR, Bongers BC, et al. Prehabilitation before major intra-abdominal cancer surgery: A systematic review of randomised controlled trials. *Eur J Anaesthesiol* 2019;36(12):933-45. doi: 10.1097/eja.0000000000001030 [published Online First: 2019/06/13]
40. Martin D, Besson C, Pache B, et al. Feasibility of a prehabilitation program before major abdominal surgery: a pilot prospective study. *J Int Med Res* 2021;49(11):3000605211060196. doi: 10.1177/03000605211060196 [published Online First: 2021/12/02]
41. Ricketts WM, Bollard K, Streets E, et al. Feasibility of setting up a pre-operative optimisation 'pre-hab' service for lung cancer surgery in the UK. *Perioperative Medicine* 2020;9(1):14. doi: 10.1186/s13741-020-00145-5
42. Hughes MJ, Hackney RJ, Lamb PJ, et al. Prehabilitation Before Major Abdominal Surgery: A Systematic Review and Meta-analysis. *World J Surg* 2019;43(7):1661-68. doi: 10.1007/s00268-019-04950-y [published Online First: 2019/02/23]
43. O'Doherty AF, West M, Jack S, et al. Preoperative aerobic exercise training in elective intra-cavity surgery: a systematic review. *Br J Anaesth* 2013;110(5):679-89. doi: 10.1093/bja/aes514 [published Online First: 2013/02/09]
44. Loughney LA, West MA, Kemp GJ, et al. Exercise interventions for people undergoing multimodal cancer treatment that includes surgery. *Cochrane Database Syst Rev* 2018;12(12):Cd012280. doi: 10.1002/14651858.CD012280.pub2 [published Online First: 2018/12/12]
45. Minnella EM, Awasthi R, Loiselle S-E, et al. Effect of Exercise and Nutrition Prehabilitation on Functional Capacity in Esophagogastric Cancer Surgery: A Randomized Clinical Trial. *JAMA Surgery* 2018;153(12):1081-89. doi: 10.1001/jamasurg.2018.1645
46. Vermillion SA, James A, Dorrell RD, et al. Preoperative exercise therapy for gastrointestinal cancer patients: a systematic review. *Syst Rev* 2018;7(1):103. doi: 10.1186/s13643-018-0771-0 [published Online First: 2018/07/26]
47. Agarwal E, Ferguson M, Banks M, et al. Malnutrition and poor food intake are associated with prolonged hospital stay, frequent readmissions, and greater in-hospital mortality: Results from the

For peer review only - http://bmjopen.bmj.com/site/about/guidelines.xhtml

Nutrition Care Day Survey 2010. *Clin Nutr* 2013;32(5):737-45. doi: https://doi.org/10.1016/j.clnu.2012.11.021
48. Curtis LJ, Bernier P, Jeejeebhoy K, et al. Costs of hospital malnutrition. *Clin Nutr* 2017;36(5):1391-96. doi: https://doi.org/10.1016/j.clnu.2016.09.009
49. Burden S, Todd C, Hill J, et al. Pre-operative nutrition support in patients undergoing gastrointestinal surgery. *Cochrane database of systematic reviews (Online)* 2012;11:CD008879.
50. Elia M, Normand C, Norman K, et al. A systematic review of the cost and cost effectiveness of using standard oral nutritional supplements in the hospital setting. *Clin Nutr* 2016;35(2):370-80. doi: 10.1016/j.clnu.2015.05.010 [published Online First: 2015/07/01]
51. Cawood AL, Elia M, Stratton RJ. Systematic review and meta-analysis of the effects of high protein oral nutritional supplements. *Ageing Research Reviews* 2012;11(2):278-96. doi: https://doi.org/10.1016/j.arr.2011.12.008
52. Ligthart-Melis GC, Luiking YC, Kakourou A, et al. Frailty, Sarcopenia, and Malnutrition Frequently (Co-)occur in Hospitalized Older Adults: A Systematic Review and Meta-analysis. *J Am Med Dir Assoc* 2020;21(9):1216-28. doi: https://doi.org/10.1016/j.jamda.2020.03.006
53. Gillis C, Davies SJ, Carli F, et al. Current Landscape of Nutrition Within Prehabilitation Oncology Research: A Scoping Review. *Front Nutr* 2021;8:644723. doi: 10.3389/fnut.2021.644723 [published Online First: 2021/04/27]
54. Underwood P, Askari R, Hurwitz S, et al. Preoperative A1C and clinical outcomes in patients with diabetes undergoing major noncardiac surgical procedures. *Diabetes Care* 2014;37(3):611-6. doi: 10.2337/dc13-1929 [published Online First: 2013/10/31]
55. Wong J, An D, Urman RD, et al. Society for Perioperative Assessment and Quality Improvement (SPAQI) Consensus Statement on Perioperative Smoking Cessation. *Anesth Analg* 2020;131(3):955-68. doi: 10.1213/ane.0000000000004508 [published Online First: 2019/11/26]
56. Madjdpour C, Heindl V, Spahn DR. Risks, benefits, alternatives and indications of allogenic blood transfusions. *Minerva Anestesiol* 2006;72(5):283-98. [published Online First: 2006/05/06]
57. Mehra T, Seifert B, Bravo-Reiter S, et al. Implementation of a patient blood management monitoring and feedback program significantly reduces transfusions and costs. *Transfusion* 2015;55(12):2807-15. doi: 10.1111/trf.13260 [published Online First: 2015/08/13]
58. Levett DZH, Grimmett C. Psychological factors, prehabilitation and surgical outcomes: evidence and future directions. *Anaesthesia* 2019;74 Suppl 1:36-42. doi: 10.1111/anae.14507 [published Online First: 2019/01/04]
59. Grimmett C, Bradbury K, Dalton SO, et al. The Role of Behavioral Science in Personalized Multimodal Prehabilitation in Cancer. *Front Psychol* 2021;12:634223. doi: 10.3389/fpsyg.2021.634223 [published Online First: 2021/03/06]
60. Emery CF, Kiecolt-Glaser JK, Glaser R, et al. Exercise accelerates wound healing among healthy older adults: a preliminary investigation. *J Gerontol A Biol Sci Med Sci* 2005;60(11):1432-6. doi: 10.1093/gerona/60.11.1432 [published Online First: 2005/12/13]
61. Lemanu DP, Singh PP, MacCormick AD, et al. Effect of preoperative exercise on cardiorespiratory function and recovery after surgery: a systematic review. *World J Surg* 2013;37(4):711-20. doi: 10.1007/s00268-012-1886-4 [published Online First: 2013/01/08]
62. West MA, Jack S, Grocott MPW. Prehabilitation before surgery: Is it for all patients? *Best Practice & Research Clinical Anaesthesiology* 2021;35(4):507-16. doi: https://doi.org/10.1016/j.bpa.2021.01.001
63. Jammer I, Wickboldt N, Sander M, et al. Standards for definitions and use of outcome measures for clinical effectiveness research in perioperative medicine: European Perioperative Clinical Outcome (EPCO) definitions: a statement from the ESA-ESICM joint taskforce on perioperative outcome measures. *Eur J Anaesthesiol* 2015;32(2):88-105. doi: 10.1097/EJA.0000000000000118 [published Online First: 2014/07/25]

For peer review only - http://bmjopen.bmj.com/site/about/guidelines.xhtml

64. Dindo D, Demartines N, Clavien PA. Classification of surgical complications: a new proposal with evaluation in a cohort of 6336 patients and results of a survey. *Ann Surg* 2004;240(2):205-13. doi: 10.1097/01.sla.0000133083.54934.ae [published Online First: 2004/07/27]
65. Clavien PA, Barkun J, de Oliveira ML, et al. The Clavien-Dindo classification of surgical complications: five-year experience. *Ann Surg* 2009;250(2):187-96. doi: 10.1097/SLA.0b013e3181b13ca2 [published Online First: 2009/07/30]
66. Slankamenac K, Graf R, Barkun J, et al. The comprehensive complication index: a novel continuous scale to measure surgical morbidity. *Ann Surg* 2013;258(1):1-7. doi: 10.1097/SLA.0b013e318296c732 [published Online First: 2013/06/04]
67. Slankamenac K, Nederlof N, Pessaux P, et al. The comprehensive complication index: a novel and more sensitive endpoint for assessing outcome and reducing sample size in randomized controlled trials. *Ann Surg* 2014;260(5):757-62; discussion 62-3. doi: 10.1097/sla.0000000000000948 [published Online First: 2014/11/08]
68. Furrer MA, Huesler J, Fellmann A, et al. The Comprehensive Complication Index CCI: A proposed modification to optimize short-term complication reporting after cystectomy and urinary diversion. *Urologic oncology* 2019;37(4):291 e9-91 e18. doi: 10.1016/j.urolonc.2018.12.013
69. Loffel LM, Gross T, Schneider MP, et al. Complication reporting with the Bern Comprehensive Complication Index CCI after open radical prostatectomy: A longitudinal long-term single-center study. *Urologic oncology* 2020;38(3):79 e1-79 e8. doi: 10.1016/j.urolonc.2019.09.021
70. Staiger RD, Cimino M, Javed A, et al. The Comprehensive Complication Index (CCI®) is a Novel Cost Assessment Tool for Surgical Procedures. *Ann Surg* 2018;268(5):784-91. doi: 10.1097/sla.0000000000002902 [published Online First: 2018/10/03]
71. Rockwood K, Song X, MacKnight C, et al. A global clinical measure of fitness and frailty in elderly people. *CMAJ* 2005;173(5):489-95. doi: 10.1503/cmaj.050051 [published Online First: 2005/09/01]
72. Aucoin SD, Hao M, Sohi R, et al. Accuracy and Feasibility of Clinically Applied Frailty Instruments before Surgery: A Systematic Review and Meta-analysis. *Anesthesiology* 2020;133(1):78-95. doi: 10.1097/aln.0000000000003257 [published Online First: 2020/04/04]
73. Kondrup J, Rasmussen HH, Hamberg O, et al. Nutritional risk screening (NRS 2002): a new method based on an analysis of controlled clinical trials. *Clin Nutr* 2003;22(3):321-36. doi: 10.1016/s0261-5614(02)00214-5 [published Online First: 2003/05/27]
74. Ottery FD. Definition of standardized nutritional assessment and interventional pathways in oncology. *Nutrition* 1996;12(1 Suppl):S15-9. doi: 10.1016/0899-9007(96)90011-8 [published Online First: 1996/01/01]
75. Ballmer P, Meier R, Möltgen C, et al. Richtlinien der GESKES über Home Care, künstliche Ernährung zu Hause. *Abgerufen am* 2013;13:2021.
76. Weimann A, Braga M, Carli F, et al. ESPEN practical guideline: Clinical nutrition in surgery. *Clinical Nutrition* 2021;40(7):4745-61. doi: https://doi.org/10.1016/j.clnu.2021.03.031
77. Inzucchi SE, Bergenstal RM, Buse JB, et al. Management of hyperglycaemia in type 2 diabetes, 2015: a patient-centred approach. Update to a position statement of the American Diabetes Association and the European Association for the Study of Diabetes. *Diabetologia* 2015;58(3):429-42. doi: 10.1007/s00125-014-3460-0 [published Online First: 2015/01/15]
78. Balci B, Kilinc G, Calik B, et al. The association between preoperative 25-OH vitamin D levels and postoperative complications in patients undergoing colorectal cancer surgery. *BMC Surgery* 2021;21(1):369. doi: 10.1186/s12893-021-01369-y
79. Iglar PJ, Hogan KJ. Vitamin D status and surgical outcomes: a systematic review. *Patient Saf Surg* 2015;9:14. doi: 10.1186/s13037-015-0060-y [published Online First: 2015/05/01]
80. Recommendations abstracted from the American Geriatrics Society Consensus Statement on vitamin D for Prevention of Falls and Their Consequences. *J Am Geriatr Soc* 2014;62(1):147-52. doi: 10.1111/jgs.12631 [published Online First: 2013/12/20]

For peer review only - http://bmjopen.bmj.com/site/about/guidelines.xhtml

81. Duceppe E, Patel A, Chan MTV, et al. Preoperative N-Terminal Pro-B-Type Natriuretic Peptide and Cardiovascular Events After Noncardiac Surgery: A Cohort Study. *Ann Intern Med* 2020;172(2):96-104. doi: 10.7326/m19-2501 [published Online First: 2019/12/24]
82. Chan AW, Tetzlaff JM, Gøtzsche PC, et al. SPIRIT 2013 explanation and elaboration: guidance for protocols of clinical trials. *Bmj* 2013;346:e7586. doi: 10.1136/bmj.e7586 [published Online First: 2013/01/11]

For peer review only

For peer review only - http://bmjopen.bmj.com/site/about/guidelines.xhtml

Figure 1: Study Flow Chart. CFS = clinical frailty scale, CPET = cardiopulmonary exercise testing

Figure 2: Nutrition workflow. NRS-2002 = Nutrition Risk Screening 2002, PG-SGA = The Scored Patient Generated Subjective Global Assessment, RBC Folate = Erythrocyte Folate, Hb = Haemoglobin, eGFR = estimated Glomerular Filtration Rate, HbA1c = glycated haemoglobin, BW = bodyweight, BMR = Basal Metabolic Rate.

For peer review only

For peer review only - http://bmjopen.bmj.com/site/about/guidelines.xhtml

Figure 1: Study Flow Chart

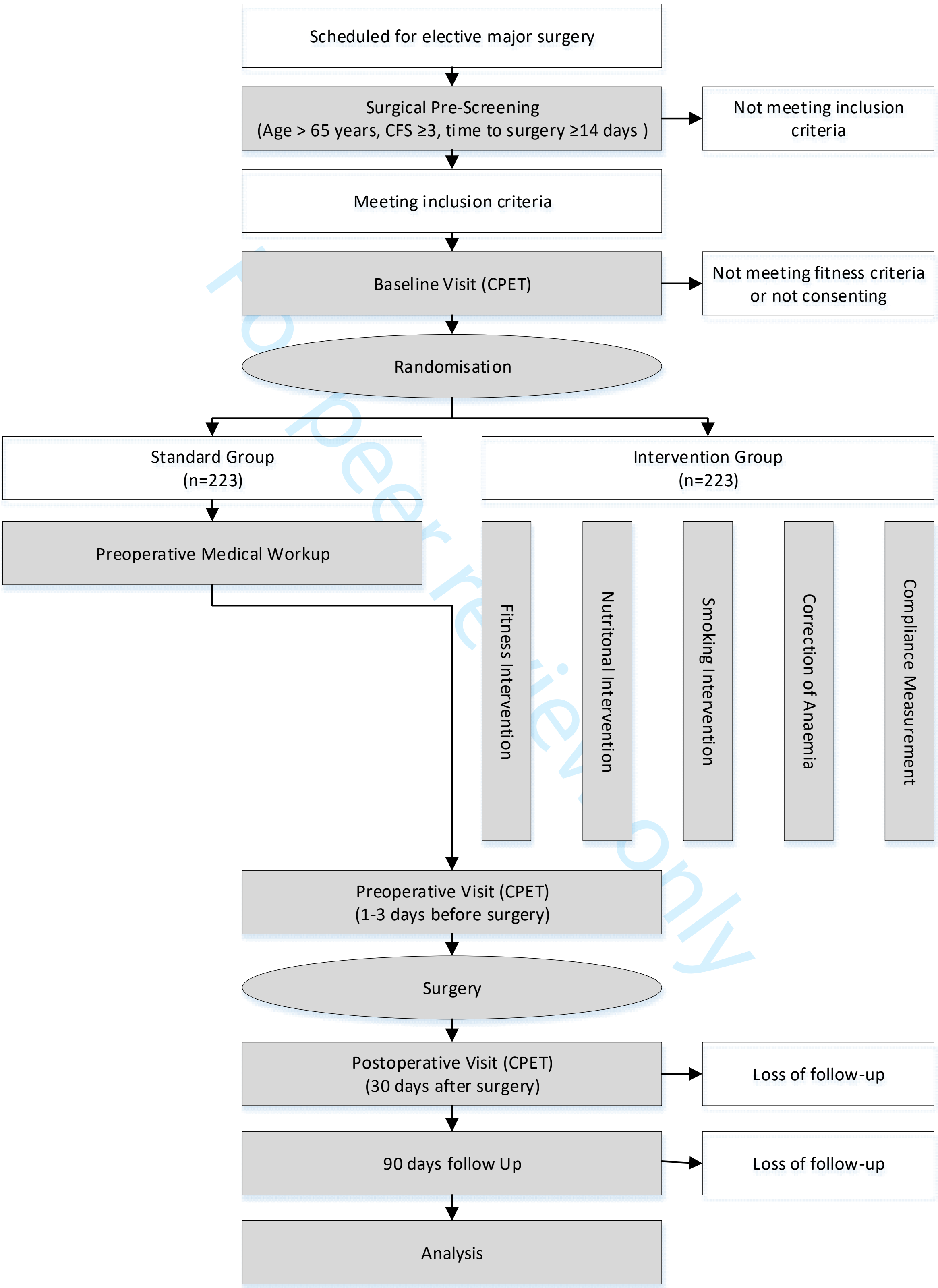

Figure 2: Nutrition workflow.

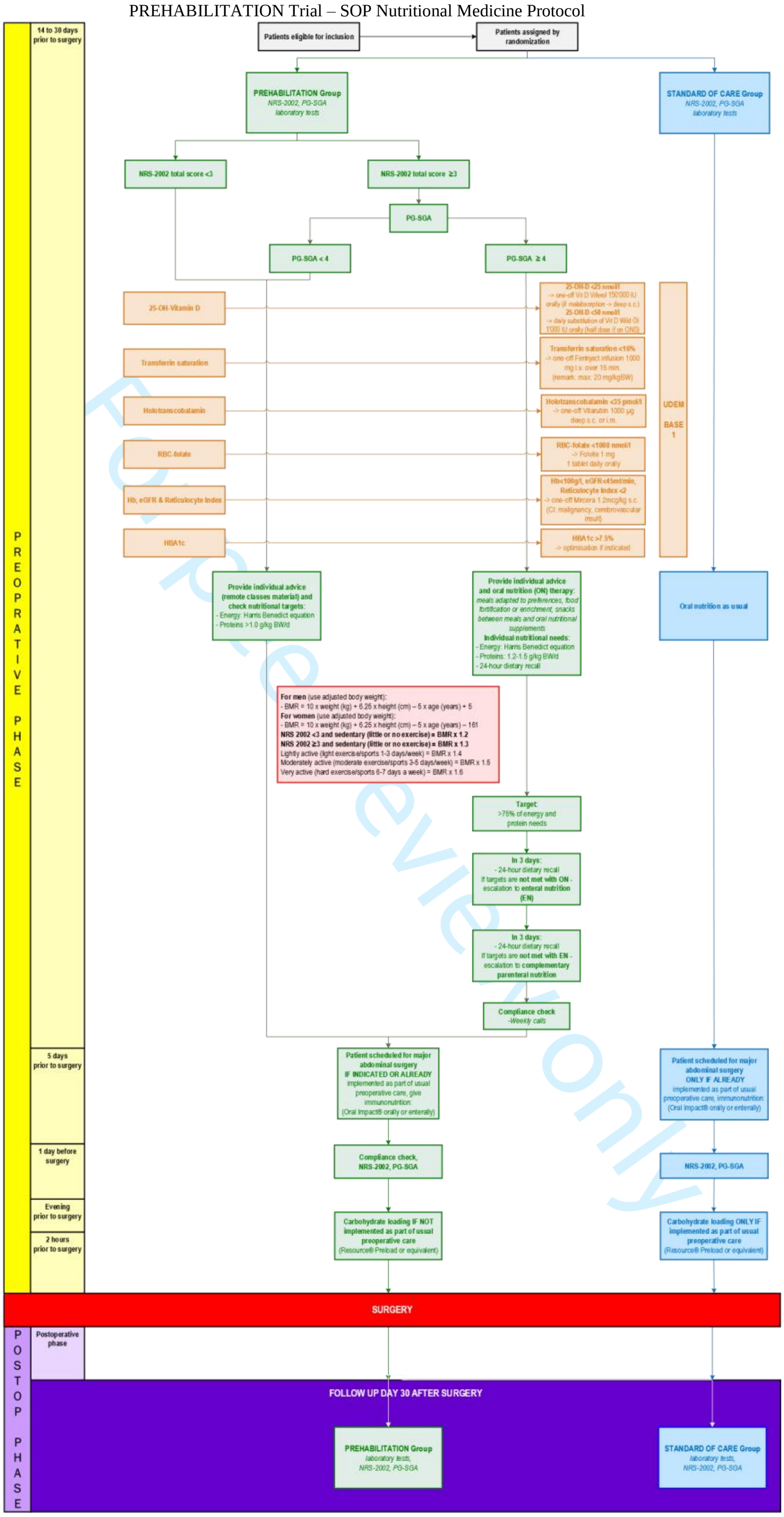

Reporting checklist for protocol of a clinical trial.

Based on the SPIRIT guidelines.

Instructions to authors

Complete this checklist by entering the page numbers from your manuscript where readers will find each of the items listed below.

Your article may not currently address all the items on the checklist. Please modify your text to include the missing information. If you are certain that an item does not apply, please write "n/a" and provide a short explanation.

Upload your completed checklist as an extra file when you submit to a journal.

In your methods section, say that you used the SPIRITreporting guidelines, and cite them as:

Chan A-W, Tetzlaff JM, Gøtzsche PC, Altman DG, Mann H, Berlin J, Dickersin K, Hróbjartsson A, Schulz KF, Parulekar WR, Krleža-Jerić K, Laupacis A, Moher D. SPIRIT 2013 Explanation and Elaboration: Guidance for protocols of clinical trials. BMJ. 2013;346:e7586

		Reporting Item	Page Number
**Administrative information**			
Title	#1	Descriptive title identifying the study design, population, interventions, and, if applicable, trial acronym	1
Trial registration	#2a	Trial identifier and registry name. If not yet registered, name of intended registry	2
Trial registration: data set	#2b	All items from the World Health Organization Trial Registration Data Set	2
Protocol version	#3	Date and version identifier	2
Funding	#4	Sources and types of financial, material, and other support	2
Roles and responsibilities: contributorship	#5a	Names, affiliations, and roles of protocol contributors	1, 14

For peer review only

Roles and responsibilities: sponsor contact information	#5b	Name and contact information for the trial sponsor	14
Roles and responsibilities: sponsor and funder	#5c	Role of study sponsor and funders, if any, in study design; collection, management, analysis, and interpretation of data; writing of the report; and the decision to submit the report for publication, including whether they will have ultimate authority over any of these activities	14
Roles and responsibilities: committees	#5d	Composition, roles, and responsibilities of the coordinating centre, steering committee, endpoint adjudication committee, data management team, and other individuals or groups overseeing the trial, if applicable (see Item 21a for data monitoring committee)	n/a, IICT
**Introduction**			
Background and rationale	#6a	Description of research question and justification for undertaking the trial, including summary of relevant studies (published and unpublished) examining benefits and harms for each intervention	3
Background and rationale: choice of comparators	#6b	Explanation for choice of comparators	3
Objectives	#7	Specific objectives or hypotheses	6
Trial design	#8	Description of trial design including type of trial (eg, parallel group, crossover, factorial, single group), allocation ratio, and framework (eg, superiority, equivalence, non-inferiority, exploratory)	7
**Methods: Participants, interventions, and outcomes**			
Study setting	#9	Description of study settings (eg, community clinic, academic hospital) and list of countries where data will be collected. Reference to where list of study sites can be obtained	7

For peer review only

Eligibility criteria	#10	Inclusion and exclusion criteria for participants. If applicable, eligibility criteria for study centres and individuals who will perform the interventions (eg, surgeons, psychotherapists)	8
Interventions: description	#11a	Interventions for each group with sufficient detail to allow replication, including how and when they will be administered	9
Interventions: modifications	#11b	Criteria for discontinuing or modifying allocated interventions for a given trial participant (eg, drug dose change in response to harms, participant request, or improving / worsening disease)	n/a, not intended
Interventions: adherance	#11c	Strategies to improve adherence to intervention protocols, and any procedures for monitoring adherence (eg, drug tablet return; laboratory tests)	11
Interventions: concomitant care	#11d	Relevant concomitant care and interventions that are permitted or prohibited during the trial	9
Outcomes	#12	Primary, secondary, and other outcomes, including the specific measurement variable (eg, systolic blood pressure), analysis metric (eg, change from baseline, final value, time to event), method of aggregation (eg, median, proportion), and time point for each outcome. Explanation of the clinical relevance of chosen efficacy and harm outcomes is strongly recommended	12
Participant timeline	#13	Time schedule of enrolment, interventions (including any run-ins and washouts), assessments, and visits for participants. A schematic diagram is highly recommended (see Figure)	20
Sample size	#14	Estimated number of participants needed to achieve study objectives and how it was determined, including clinical and statistical assumptions supporting any sample size calculations	13
Recruitment	#15	Strategies for achieving adequate participant enrolment to reach target sample size	13
**Methods: Assignment of interventions (for controlled trials)**			
Allocation: sequence generation	#16a	Method of generating the allocation sequence (eg, computer-generated random numbers), and list of any factors for stratification. To reduce predictability of a random sequence, details of any planned restriction (eg, blocking) should be	9

For peer review only

		provided in a separate document that is unavailable to those who enrol participants or assign interventions	
Allocation concealment mechanism	#16b	Mechanism of implementing the allocation sequence (eg, central telephone; sequentially numbered, opaque, sealed envelopes), describing any steps to conceal the sequence until interventions are assigned	9
Allocation: implementation	#16c	Who will generate the allocation sequence, who will enrol participants, and who will assign participants to interventions	9
Blinding (masking)	#17a	Who will be blinded after assignment to interventions (eg, trial participants, care providers, outcome assessors, data analysts), and how	11
Blinding (masking): emergency unblinding	#17b	If blinded, circumstances under which unblinding is permissible, and procedure for revealing a participant's allocated intervention during the trial	n/a, not blinded
**Methods: Data collection, management, and analysis**			
Data collection plan	#18a	Plans for assessment and collection of outcome, baseline, and other trial data, including any related processes to promote data quality (eg, duplicate measurements, training of assessors) and a description of study instruments (eg, questionnaires, laboratory tests) along with their reliability and validity, if known. Reference to where data collection forms can be found, if not in the protocol	11
Data collection plan: retention	#18b	Plans to promote participant retention and complete follow-up, including list of any outcome data to be collected for participants who discontinue or deviate from intervention protocols	11
Data management	#19	Plans for data entry, coding, security, and storage, including any related processes to promote data quality (eg, double data entry; range checks for data values). Reference to where details of data management procedures can be found, if not in the protocol	11
Statistics: outcomes	#20a	Statistical methods for analysing primary and secondary	13

For peer review only

		outcomes. Reference to where other details of the statistical analysis plan can be found, if not in the protocol	
Statistics: additional analyses	#20b	Methods for any additional analyses (eg, subgroup and adjusted analyses)	13
Statistics: analysis population and missing data	#20c	Definition of analysis population relating to protocol non-adherence (eg, as randomised analysis), and any statistical methods to handle missing data (eg, multiple imputation)	13
**Methods: Monitoring**			
Data monitoring: formal committee	#21a	Composition of data monitoring committee (DMC); summary of its role and reporting structure; statement of whether it is independent from the sponsor and competing interests; and reference to where further details about its charter can be found, if not in the protocol. Alternatively, an explanation of why a DMC is not needed	12
Data monitoring: interim analysis	#21b	Description of any interim analyses and stopping guidelines, including who will have access to these interim results and make the final decision to terminate the trial	n/a, not intended
Harms	#22	Plans for collecting, assessing, reporting, and managing solicited and spontaneously reported adverse events and other unintended effects of trial interventions or trial conduct	13
Auditing	#23	Frequency and procedures for auditing trial conduct, if any, and whether the process will be independent from investigators and the sponsor	12
**Ethics and dissemination**			
Research ethics approval	#24	Plans for seeking research ethics committee / institutional review board (REC / IRB) approval	8
Protocol amendments	#25	Plans for communicating important protocol modifications (eg, changes to eligibility criteria, outcomes, analyses) to relevant parties (eg, investigators, REC / IRBs, trial participants, trial registries, journals, regulators)	8
Consent or assent	#26a	Who will obtain informed consent or assent from potential trial participants or authorised surrogates, and how (see Item 32)	9

For peer review only

Consent or assent: ancillary studies	#26b	Additional consent provisions for collection and use of participant data and biological specimens in ancillary studies, if applicable	n/a, no provisions intended
Confidentiality	#27	How personal information about potential and enrolled participants will be collected, shared, and maintained in order to protect confidentiality before, during, and after the trial	11
Declaration of interests	#28	Financial and other competing interests for principal investigators for the overall trial and each study site	14
Data access	#29	Statement of who will have access to the final trial dataset, and disclosure of contractual agreements that limit such access for investigators	11
Ancillary and post trial care	#30	Provisions, if any, for ancillary and post-trial care, and for compensation to those who suffer harm from trial participation	na
Dissemination policy: trial results	#31a	Plans for investigators and sponsor to communicate trial results to participants, healthcare professionals, the public, and other relevant groups (eg, via publication, reporting in results databases, or other data sharing arrangements), including any publication restrictions	13
Dissemination policy: authorship	#31b	Authorship eligibility guidelines and any intended use of professional writers	14
Dissemination policy: reproducible research	#31c	Plans, if any, for granting public access to the full protocol, participant-level dataset, and statistical code	14
**Appendices**			
Informed consent materials	#32	Model consent form and other related documentation given to participants and authorised surrogates	23
Biological specimens	#33	Plans for collection, laboratory evaluation, and storage of biological specimens for genetic or molecular analysis in the current trial and for future use in ancillary studies, if applicable	32

Notes:

- 11b: n/a, not intended
- 17b: n/a, not blinded
- 21b: n/a, not intended

For peer review only

- 26b: n/a, no provisions intended The SPIRIT Explanation and Elaboration paper is distributed under the terms of the Creative Commons Attribution License CC-BY-NC. This checklist was completed on 14. November 2022 using https://www.goodreports.org/, a tool made by the EQUATOR Network in collaboration with Penelope.ai

For peer review only
