# Supplementary material for: Multimodal prehabilitation for major surgery in elderly patients to lower complications: protocol of a randomised, prospective, multicentre, multidisciplinary trial (PREHABIL Trial)
Source: BMJ Open. 2023 Jan 3;13(1):e070253. doi: 10.1136/bmjopen-2022-070253 (PMC9815025; doi:10.1136/bmjopen-2022-070253)
Supplement: Reviewer comments [file bmjopen-2022-070253.reviewer_comments.pdf]

PEER REVIEW HISTORY

BMJ Open publishes all reviews undertaken for accepted manuscripts. Reviewers are asked to complete a checklist review form (**http://bmjopen.bmj.com/site/about/resources/checklist.pdf**) and are provided with free text boxes to elaborate on their assessment. These free text comments are reproduced below.

ARTICLE DETAILS

**TITLE (PROVISIONAL)**	Multimodal prehabilitation for major surgery in elderly patients to lower complications: Protocol of a randomised, prospective, multi-centre, multidisciplinary trial (PREHABIL Trial).
**AUTHORS**	Beilstein, Christian; Krutkyte, Gabija; Vetsch, Thomas; Eser, Prisca; Wilhelm, M; Stanga, Zeno; Bally, Lia; Verra, Martin; Huber, Markus; Wuethrich, Patrick; Engel, Dominique

This article was not externally reviewed at BMJ Open. Protocols that have been independently assessed prior to submission to BMJ Open are usually fast-tracked to publication on the grounds that further substantial changes will not be possible. This independent assessment will usually be external, independent review for both a substantial grant award from a non-commercial or government funder and ethics approval.
